# Silylene-Stabilized Neutral Dibora-Aromatics with a B=B Bond

**DOI:** 10.1021/jacs.4c06579

**Published:** 2024-07-09

**Authors:** Jun Fan, Jian Xu, Qin Ma, Shenglai Yao, Lili Zhao, Gernot Frenking, Matthias Driess

**Affiliations:** † Department of Chemistry: Metalorganics and Inorganic Materials, Technische Universität Berlin, Strasse des 17. Juni 115, Sekr. C2, Berlin 10623, Germany; ‡ State Key Laboratory of Materials-Oriented Chemical Engineering, School of Chemistry and Molecular Engineering, Nanjing Tech University, Nanjing 211816, China; § Fachbereich Chemie, Philipps-Universität Marburg, Marburg 35032, Germany

## Abstract

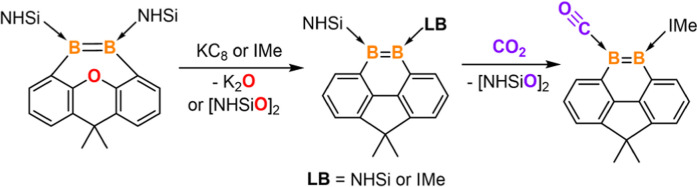

The unprecedented silylene-supported dibenzodiboraoxepin **2** and 9,10-diboraphenanthrene complexes **6** and **8** were synthesized. The (NHSi)_2_B_2_(xanthene) [NHSi = PhC(NtBu)_2_(Me_2_N)Si:] **2** results from debromination of the bis(NHSi)-stabilized bis(dibromoboryl)xanthene **1** with potassium graphite (KC_8_); **2** is capable of activating white phosphorus and ammonia to form the B_2_P_4_ cage compound **3** and H_2_N–B–B–H diborane species **4**, respectively. The thermal rearrangement of **2** affords the 9,10-dihydro-9,10-diboraphenanthrene **5** through a bis(NHSi)-assisted intramolecular reductive C–O–C deoxygenation process. Notably, the 9,10-diboraphenanthrene derivatives **6** and **8** could be generated by deoxygenation of **2** with KC_8_ and 1,3,4,5-tetramethylimidazol-2-ylidene, respectively. The aromaticity of **6** and **8** was confirmed by computational studies. Strikingly, the NHSi ligand in **8** engenders the monodeoxygenation of carbon dioxide in toluene at room temperature to form the CO-stabilized 9,10-diboraphenanthrene derivative **9** via the silaoxadiborinanone intermediate **10**.

## Introduction

**Figure 1:**
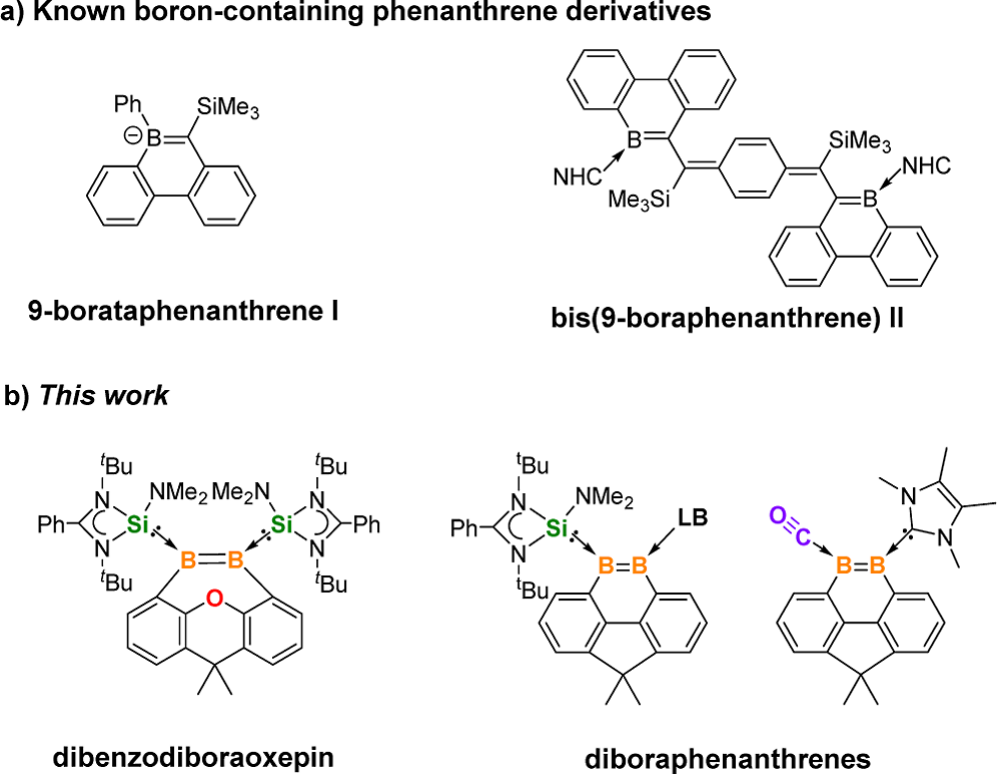
Boron-containing phenanthrene and dibenzoxepin derivatives (NHC = IPr or IMe; IPr = 1,3-diisopropylimidazol-2-ylidene; IMe = 1,3,4,5-tetramethylimidazol-2-ylidene; LB = NHSi or IMe; NHSi = PhC(NtBu)_2_(Me_2_N)Si).

Polycyclic aromatic hydrocarbons (PAHs) are an intriguing class of organic materials, which have been extensively applied in various research fields due to their extraordinary optoelectronic and photophysical properties.^1,2^ Among them, phenanthrene is a nonlinear PAH with three fused benzene rings, which was discovered in 1872. Phenanthrene and their derivatives have been widely utilized in the pharmaceutical and manufacturing industries.^3−5^ In addition, they also serve as key building blocks for the construction of extended π-conjugated materials.^6−8^ The incorporation of electron-deficient boron atoms into the specific positions of pristine PAHs is an efficient way to tune the energy level of their frontier orbitals and modulate their structural, electronic, and reactive properties as well as luminescent behaviors.^9−11^ Boron-containing anthracenes as well as their reactivity toward small molecules have been well documented.^12−18^ In contrast, the boron-containing phenanthrene derivatives are scarce. Till date, only two such examples have been reported. In 2020, Martin and co-workers isolated the first anionic 9-borataphenanthrene species **I** (Figure 1a), which exhibited the reactivity patterns of both boratabenzene and borataalkene.^19^ Recently, the Gilliard group designed and synthesized the neutral carbene-stabilized bis(9-boraphenanthrene) **II** (Figure 1a) and its diradical, which displayed intriguing luminescent behaviors.^20^ To the best of our knowledge, the diboron-containing phenanthrene derivative is hitherto unknown, presumably due to the lack of a suitable synthetic protocol.

Lewis base-stabilized diborenes are isoelectronic with alkenes. Since the isolation of the first *N*-heterocyclic carbene-stabilized parent diborene by Robinson and co-workers in 2007,^21^ a variety of diborenes, including cyclic and acyclic derivatives, supported by different Lewis bases, such as cyclic alkyl amino carbenes (cAACs),^22−24^ phosphines,^25−27^ and silylenes,^28^ have been reported. In addition, these compounds show a noteworthy reactivity in small molecule activation,^29,30^ are competent π-donor ligands toward transition metals,^31^ and are used as catalysts in homogeneous catalysis.^32^ Due to their isoelectronic relationship, using a B=B moiety to replace a C=C unit in the all-carbon PAHs and retaining their aromaticity is a promising strategy to construct diboron-containing PAHs. The expected far more reactive B=B moiety could render the resulting PAHs novel reactivity patterns. Up to now, one example of a dianionic B=B analogue of dibenzo[g,p]chrysene was reported,^33^ but a PAH analogue with a neutral B=B moiety is hitherto unknown.

Herein, we report the synthesis of the first bis(NHSi)-stabilized dibenzodiboraoxepin complex **2** bearing a neutral B=B moiety which serves as a synthon to access the unprecedented symmetric and unsymmetrical 9,10-diboraphenanthrene derivatives **6** and **8** via the deoxygenation of the xanthene backbone with potassium graphite (KC_8_) or 1,3,4,5-tetramethylimidazol-2-ylidene (the *N*-heterocyclic carbene IMe), respectively (Figure 1b). In addition, **2** is capable of reacting with P_4_ and NH_3_ to form the unusual insertion products across the B=B bond, **3** and **4**. Notably, the NHSi ligand in **8** engenders the monodeoxygenation of carbon dioxide (CO_2_) in toluene at room temperature to afford the carbon monoxide-stabilized 9,10-diboraphenanthrene derivative **9** via a silaoxadiborinanone intermediate **10**. Computational investigations confirmed aromaticity and distinct ligand effects of the NHSi, NHC, and CO ligands in **6**, **8**, and **9**.

## Results and Discussion

### Synthesis and Reactivity of Silylene-Stabilized Dibenzodiboraoxepin Complex **2**

Silylenes, the silicon analogues of carbenes, are strong σ-donor ligands and have been shown to act as efficient Lewis bases in stabilizing main-group elements and transition metals in uncommon low oxidation states.^34^ These prompted us to synthesize a NHSi-supported cyclic organodiborene complex with a xanthene scaffold. Due to the presence of a rigid xanthene spacer, we anticipated that the target compound will exhibit unique reactivity and act as a synthon for the construction of diboron-containing PAHs. To begin with, the BBr_2_(Xant)BBr_2_ (Xant = 9,9-dimethyl-xanthene-4,5-diyl) starting material is easily accessible by the σ-metathesis reaction of 4,5-bis(trimethylsilyl)Xant with 2 M equiv of BBr_3._^35^ Its treatment with 2 M equiv of the NHSi [NHSi = PhC(NtBu)_2_(Me_2_N)Si:] in toluene afforded the bis[(NHSi)-dibromoboryl]xanthene complex **1** as a colorless solid in 85% yield (see Supporting Information). The ^11^B{^1^H} NMR signal of **1** at δ −8.4 ppm indicates the presence of four-coordinate boron centers. The molecular structure of **1** obtained by X-ray crystallography shows the presence of two NHSi molecules that are ligated to the B atoms of the Br_2_B groups, hence both Si and B atoms adopt a tetrahedral coordination geometry (Figure S46).

**Scheme 1:**
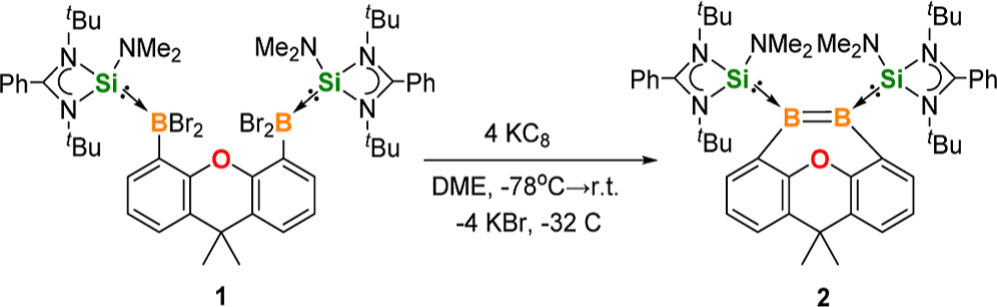
Synthesis of **2** from **1**

**Figure 2:**
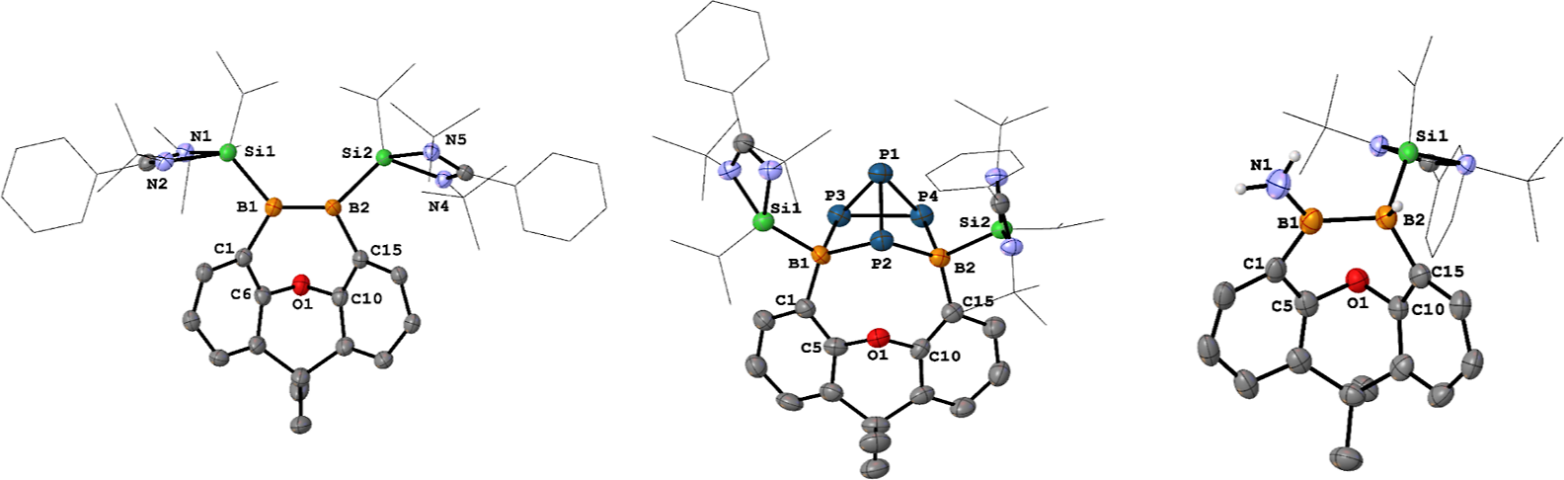
Molecular structures of **2**, **3**, and **4**. Thermal ellipsoids are drawn at the 50% probability level. H atoms and solvent molecules are omitted for clarity. Selected bond lengths (Å) and angles (deg): **2**: B1–B2 1.646(4), Si1–B1 1.967(3), Si2–B2 1.975(3), O1–C6 1.398(3), O1–C10 1.401(3), C1–B1 1.618(4), C15–B2 1.618(3), C1–B1–Si1 109.30(17), C6–O1–C10 99.28(17). **3**: Si1–B1 2.020(6), Si2–B2 2.026(5), P2–B1 2.036(6), P3–B1 1.990(5), P2–B2 2.007(6), P4–B2 1.991(5), B2–P2–B1 103.7(2), Si1–B1–P2 105.1(3). **4**: B1–B2 1.757(5), N1–B1 1.406(4), Si1–B2 1.999(3), N1–B1–B2 120.0(3), N1–B1–C1 115.2(3), B2–B1–C1 124.3(3).

The reduction of **1** with 4 M equiv of KC_8_ in 1,2-dimethoxyethane (DME) at ambient temperature for 12 h furnished the target complex **2** (Scheme 1), which was isolated as a red crystalline solid in 70% yield. The ^11^B{^1^H} NMR spectrum of **2** displays a signal at δ 25.8 ppm, which is downfield shifted in comparison with that of **1** but falls in the range of resonance values of known Lewis bases-stabilized diborenes (δ 5–52 ppm).^21−32^ The resonance in the ^29^Si{^1^H} NMR spectrum of **2** at δ 16.5 ppm is also downfield shifted compared to that of **1**. The molecular structure of **2** established by single-crystal X-ray diffraction (XRD) analysis consists of a seven-membered C_4_B_2_O dibenzodiboraoxepin-like ring with a highly distorted xanthene backbone and two phenyl ring planes with a dihedral angle of 100.9° (Figure 2, left). The two NHSi ligands in **2** are ligated to two trigonal planar coordinate boron centers in a *cis*-position, and the Si1, Si2, B1, B2, C1, and C15 atoms are coplanar positioned. Owing to the high ring strain exerted by the xanthene skeleton, the B1–B2 moiety exhibits an elongated B=B distance of 1.646(4) Å. The value surpasses those of Br(NHSi)B=B(NHSi)Br (1.56(3) Å)^28^ and cyclic bis(cAAC)-diborene complex (1.633(7) Å)^23^ and is even slightly longer than those of the IMe-diborene radical cation [IMe(Dur)B–B(Dur)IMe]^+^ (1.636(4) Å) and phosphine-diborene radical cation [PEt_3_(Dur)B–B(Dur)PEt_3_]^+^ (1.631(6) Å) (Dur = 2,3,5,6-tetramethylphenyl), respectively.^36^ It is worth noting that there is no attractive interaction between two Si^II^ centers or Si^II^ and B^III^ centers in the xanthene-based bis(NHSi)^37^ or NHSi-borane frustrated Lewis pair,^38^ while the two smaller boron centers in **2** form a B=B bond. The Si–B lengths of **2** (Si1–B1:1.967(3); Si2–B2:1.975(3) Å) are shorter than those of **1** (Si–B 2.046(4) and 2.032(4) Å) due to the formation of the B=B bond, while the C–O distances in **2** (O1–C6 1.398(3); O1–C10 1.401(3) Å) are slightly elongated as compared to those of **1** (O1–C6 1.379(4); O1–C10 1.377(4) Å).

**Scheme 2:**
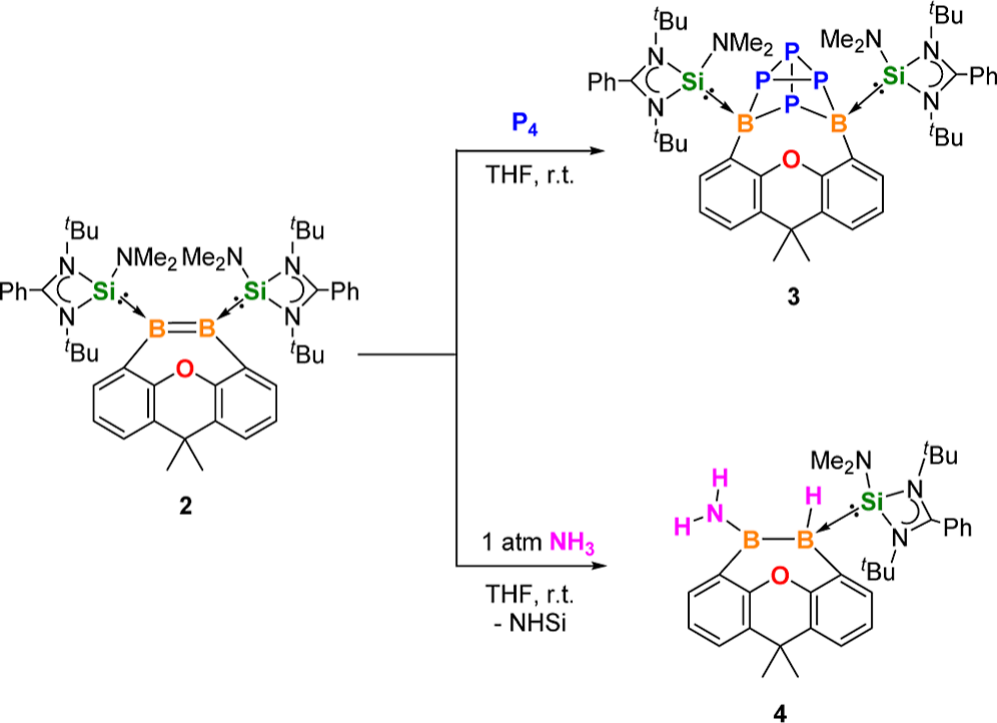
P_4_ and NH_3_ Activation by **2**

While a NHSi-supported dibromodiborene Br(NHSi)B=B(NHSi)Br has been reported previously, its reactivity remains unexplored.^28^ The B=B bond in **2** shows a remarkable reactivity toward inert bonds in small molecules, such as P_4_ and NH_3_. The reaction of **2** with 1 M equiv of P_4_ in tetrahydrofuran (THF) at ambient temperature results in a color change of the solution from red to yellow within 5 min. After workup, **3** was isolated as yellow crystals in 95% yield (Scheme 2). The ^11^B{^1^H} NMR spectrum of **3** exhibits one broad signal at δ −15.6 ppm, which is upfield shifted as compared to that of **2** (δ 25.8 ppm), indicating the formation of four-coordinate boron centers. The ^31^P{^1^H} NMR spectrum of **3** shows three broadened resonances at δ 144.9, −85.8, and −179.8 ppm. The molecular structure of **3** has been elucidated by X-ray crystallography (Figure 2, middle) and is in keeping with the information deduced from the multinuclear NMR data. Apparently, the addition of P_4_ engenders the B=B bond scission in **2** and concomitant cleavage of two adjacent P–P bonds of the P_4_ cage, resulting in the formation of two tetrahedral boron centers and three chemically inequivalent phosphorus atoms. This P_4_ activation mode is quite different from those of P_4_ activation mediated by polarized B=B bond species.^23,39^

In contrast to a N–H bond activation with primary amines using bis(SI^Dep^) diboryne (SI^Dep^ = 1,3-bis(2,6-diethylphenyl)imidazolin-2-ylidene) complex,^40^ such N–H bond activation of NH_3_ using a diborene system is currently unknown. We now learned that the elongated B=B bond in **2** engenders facile N–H bond activation of NH_3_ (1 atm) in THF at room temperature to form selectively the HB-BNH_2_ addition product **4** as a colorless crystalline solid in 65% yield (Scheme 2). Remarkably, during the NH_3_ addition, one NHSi ligand is liberated presumably because of the efficient B=N π-interaction of the H_2_N-boryl group in **4**. The ^11^B NMR spectrum of **4** shows a singlet at δ 52.1 ppm and a doublet at δ −34.8 ppm, corresponding to the three-coordinate *B*NH_2_ and four-coordinate *B*H group, respectively. The molecular structure of **4** established by an XRD analysis is in accordance with the multinuclear NMR data, in which the B1 and B2 centers adopt trigonal planar and tetrahedral coordination geometries, respectively (Figure 2, right). The B1–B2 distance of 1.757(5) Å is in the upper range for a B–B single bond (1.682(16)–1.762(11) Å).^41^

### Synthesis of 9,10-Diboraphenanthrene Derivatives **6** and **8** via Reductive Deoxygenation of the Xanthene Backbone in 2

**Scheme 3:**
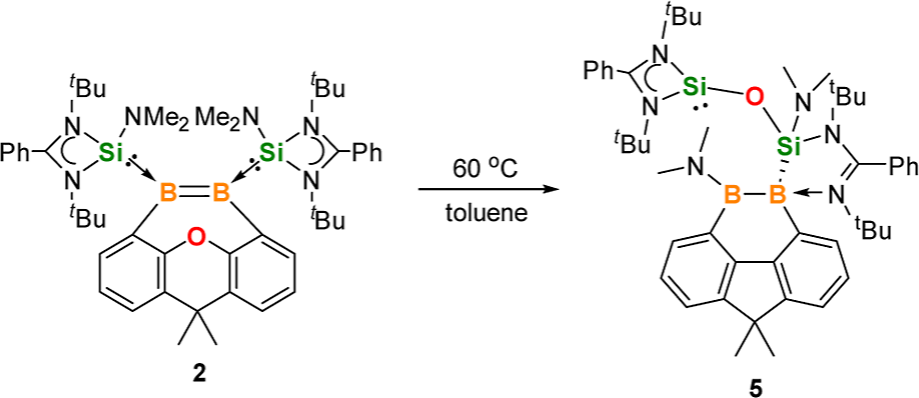
Thermal Rearrangement of **2**

Owing to its high ring strain, we anticipated that **2** is thermolabile if heated in an inert solvent. In fact, heating **2** in toluene at 60 °C for 5 h resulted in the gradual color change of the solution from red to yellow. After workup, compound **5** was isolated as a pale yellow crystalline solid in 69% yield (Scheme 3). The ^11^B{^1^H} NMR spectrum of **5** shows two signals at δ 55.0 and −19.5 ppm, indicating the presence of a sp^2^ B1 and sp^3^-hybridized B2 center, respectively. The ^29^Si{^1^H} NMR spectrum of **5** displays also two resonances at δ −22.3 and 21.5 ppm which are attributed to the three-coordinate silylene- and four-coordinate silane-^29^Si nuclei, respectively.

**Figure 3:**
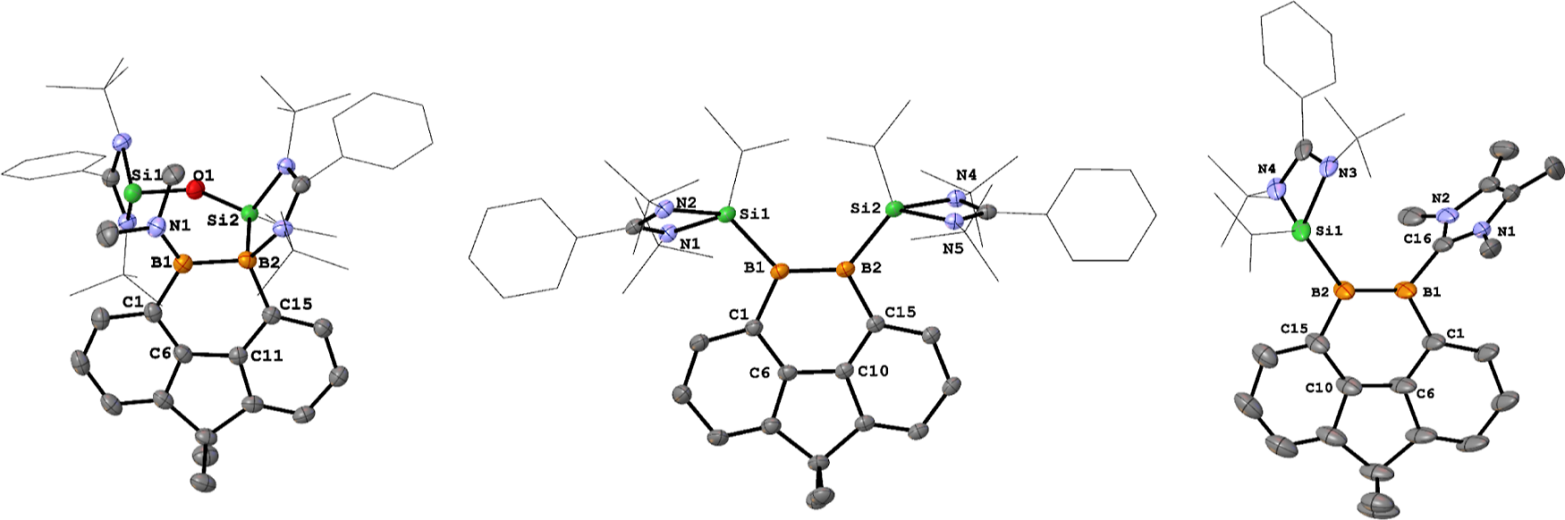
Molecular structures of **5**, **6**, and **8**. Thermal ellipsoids are drawn at the 50% probability level. H atoms and solvent molecules are omitted for clarity. Selected bond lengths (Å) and angles (deg): **5**: B1–B2 1.743(3), N1–B1 1.420(3), Si2–B2 1.994(2), C1–B1–B2 116.26(16), N1–B1–C1 118.95(18), N1–B1–B2 124.78(18). **6**: B1–B2 1.650(4), B1–C1 1.567(4), C1–C6 1.411(4), C6–C10 1.431(4), C10–C15 1.413(4), C15–B2 1.571(4), C1–B1–Si1 112.0(2), C1–B1–B2 116.4(2), B2–B1–Si1 131.4(2), C15–B2–Si2 112.4(2), C15–B2–B1 116.7(2), B1–B2–Si2 130.8(2). **8**: B2–B1 1.623(7), B1–C1 1.566(6), C1–C6 1.411(6), C10–C6 1.419(6), C10–C15 1.410(6), C15–B2 1.565(6), C16–B1–B2 129.5(4), C1–B1–C16 112.2(4), C1–B1–B2 118.1(4).

The molecular structure of **5** (Figure 3, left) uncovered that the xanthene backbone in **2** underwent a bis(NHSi)-assisted reductive deoxygenation to engender a 9,10-dihydro-9,10-diboraphenanthrene scaffold along with the formation of a mixed-valent disiloxane (Si^II^–O–Si^IV^) group^42a^ bonded to one of the B atoms. During the deoxygenation process, one of the dimethylamino groups of a NHSi migrates to one B center, while an imino-N atom of an amidinate group coordinates to the other B center, affording the diborane complex **5** as a rearrangement product. The two boron atoms adopt trigonal planar and tetrahedral coordination geometries, respectively, which is consistent with the different ^11^B NMR chemical shifts. The B1–B2 distance of 1.743(3) Å is similar to that in **4**.

**Scheme 4:**
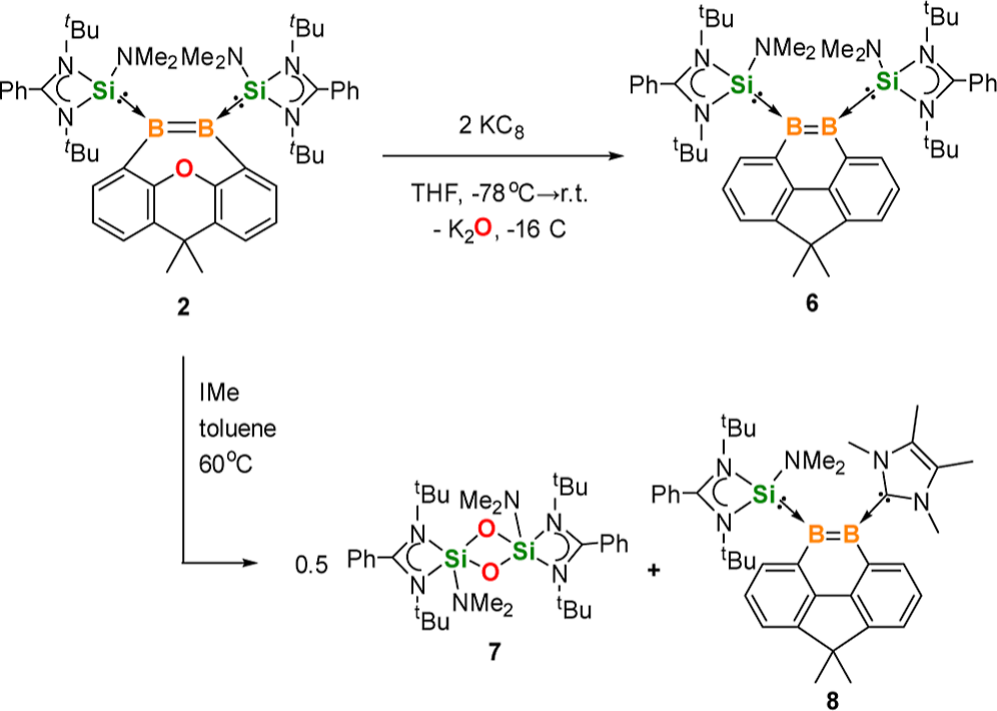
Synthesis of **6** and **8**

The mechanism of this thermal rearrangement is currently unknown but prompted us to test external reducing reagents to achieve a deoxygenation reaction of **2** with the formation of the desired diboraphenanthrene derivatives (Scheme 4). In fact, exposure of **2** to 2 M equiv of KC_8_ in THF at room temperature for 2 h furnished the bis(NHSi)-stabilized 9,10-diboraphenanthrene derivative **6**, which was isolated as the red single crystals from a saturated diethyl ether (Et_2_O) solution in 30% yield. The ^11^B{^1^H} NMR spectrum of **6** shows a signal at δ 29.2 ppm, which is comparable with that of **2** (δ 25.8 ppm). The ^29^Si{^1^H} NMR resonance of **6** at δ 17.7 ppm is also akin to that of **2** (δ 16.5 ppm). A single-crystal XRD analysis of **6** revealed a 9,10-diboraphenanthrene scaffold with a CMe_2_ linker at 4,5-positions, in which two NHSi ligands are coordinated to two trigonal planar boron atoms (Figure 3, middle). Its B1–B2 bond length of 1.650(4) Å is almost identical with that of **2** (1.646(4) Å). The elongated B=B bond in **6** is due to the π-electron delocalization.

When the alternative reducing agent IMe was allowed to react with **2** in toluene at 60 °C for 12 h, the NHSi- and IMe-stabilized unsymmetrical 9,10-diboraphenanthrene derivative **8** was formed along with the known four-membered Si_2_O_2_ ring compound **7** as a coproduct (Scheme 4).^42b^**8** was isolated as a red crystalline solid in 60% yield. Its ^11^B{^1^H} NMR spectrum displays two signals at δ 33.5 and 11.6 ppm, which are similar to those of polarized diborenes.^25,32^ Its molecular structure shows a similar diboraphenanthrene skeleton, while one NHSi ligand in **6** was replaced by IMe (Figure 3, right). The B=B distance of 1.623(7) Å is comparable with that of **6** (1.650(4) Å). To further investigate this deoxygenation reaction, 1 M equiv of NHSi or IPr (IPr = 1,3-diisopropylimidazol-2-ylidene) was reacted with **2** under the same conditions. However, the thermal rearrangement product **5** was observed, which indicated that the sterically less demanding IMe is essential for this reaction.

### Synthesis of the CO-Stabilized 9,10-Diboraphenanthrene Derivative 9 via NHSi-Assisted Monodeoxygenation of CO_2_

**Scheme 5:**
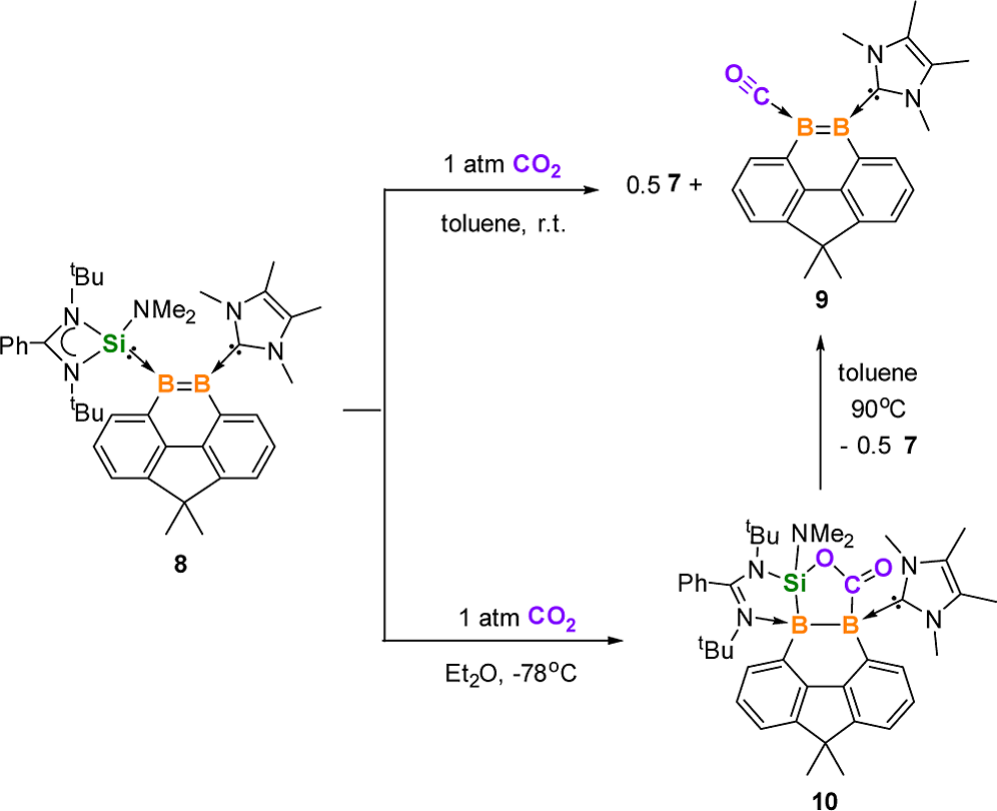
CO_2_ Activation by **8**

CO_2_ activation and transformation into value-added chemicals employing diborene species has gained significant interest. Among these studies, a [2 + 2] cycloaddition of CO_2_ with the B=B bond in diborenes has been systematically studied.^43,44^ So and co-workers reported the hydroboration of CO_2_ with pinacolborane catalyzed by a cyclic diborene complex.^32^ Furthermore, the Braunschweig group showed that cAAC-supported parent diborene could activate CO_2_ to give cAAC-stabilized parent borylene carbonyl and boroxine species.^45^ In our work, we observed that the NHSi ligand in **8** is capable of further mediating the monodeoxygenation of CO_2_ in toluene at room temperature to generate the IMe- and CO-stabilized 9,10-diboraphenanthrene derivative **9** along with the formation of the coproduct **7** (Scheme 5).

**Figure 4:**
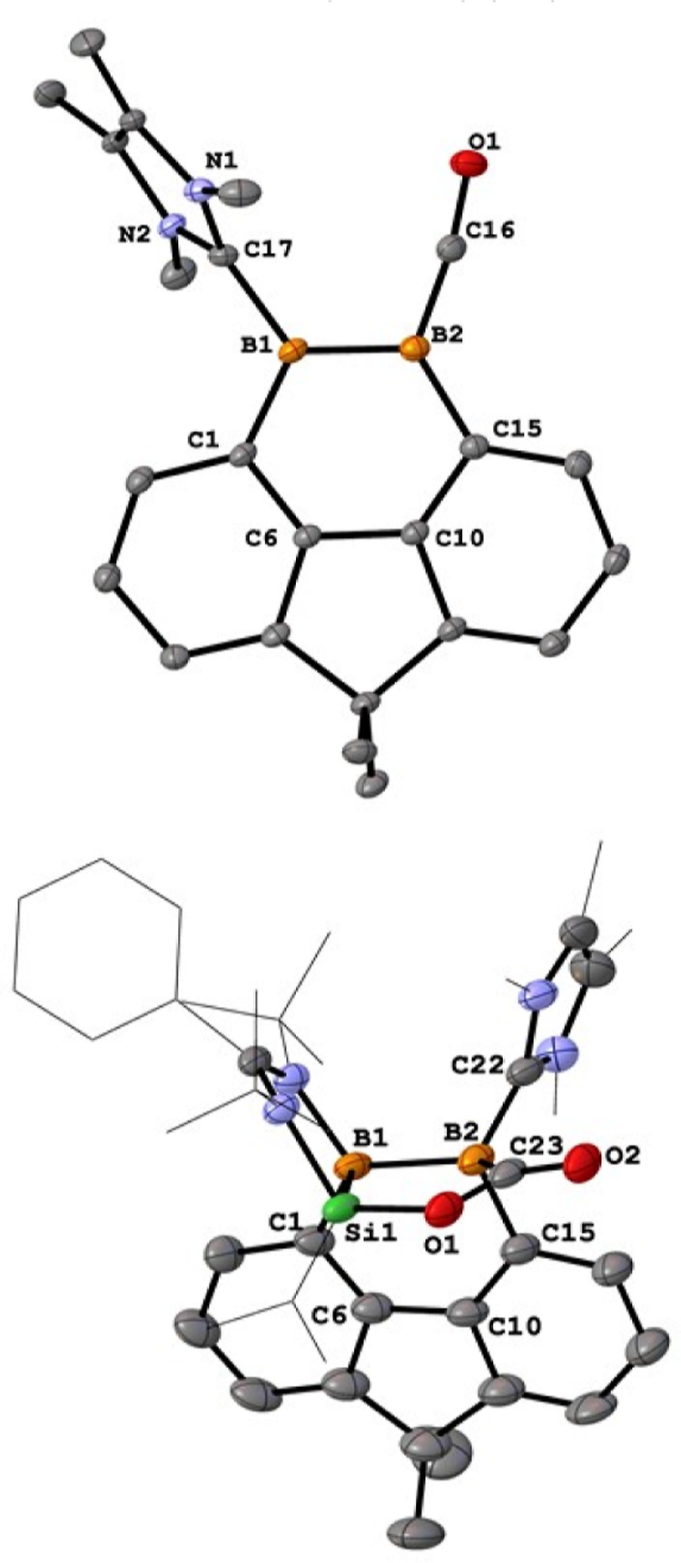
Molecular structures of **9** and **10**. Thermal ellipsoids are drawn at the 50% probability level. H atoms and solvent molecules are omitted for clarity. Selected bond lengths (Å) and angles (deg): **9**: B1–B2 1.629(5), B1–C17 1.592(4), B2–C16 1.441(5), C16–O1 1.173(4), C1–B1–B2 116.4(2), C1–B1–C17 118.6(3), C17–B1–B2 125.0(3), C15–B2–B1 120.1(3), C16–B2- B1 110.6(3), C16–B2–C15 129.2(3), O1–C16–B2 171.5(3). **10**: B1–B2 1.846(5), Si1–B1 1.956(4), Si1–O1 1.681(2), O1–C23 1.377(5), O2–C23 1.229(4), C23–B2 1.634(5), C23–B2–B1 110.0(3), O1–C23–B2 116.0(3), B2–B1–Si1 92.3(2).

The reaction of **8** with CO_2_ (1 atm) in toluene at room temperature resulted in a color change of the solution from red to yellow within 3 min. After workup, the major products **7** and **9** were isolated as colorless and yellow single crystals. Two resonances at δ 63.5 and −11.7 ppm were observed in the ^11^B{^1^H} NMR spectrum of **9**, which are attributed to the IMe- and CO-coordinated B centers, respectively. These results are consistent with its natural population analysis (NPA, Table S21), where the B_NHC_ atom has a positive charge of 0.2*e*, while the B_CO_ atom has a negative charge of −0.2*e*. The large difference of the two ^11^B{^1^H} NMR signals indicates that the B=B bond is highly polarized. The molecular structure of **9** exhibits a similar structure to **8**, with the NHSi ligand being replaced by CO (Figure 4, top). Its B=B distance of 1.629(5) is akin to that of **8** (1.623(7) Å).

When the reaction was conducted in Et_2_O at −78 °C, compound **10** was isolated as yellow crystals in 83% yield as the major product (Scheme 5). The ^11^B{^1^H} NMR spectrum of **10** shows two signals at δ −11.1 and −16.9 ppm, indicating the presence of two four-coordinate boron atoms. Its molecular structure obtained by XRD analysis shows that the silylene and diborene moieties in **8** cooperatively activated CO_2_ to form a five-membered SiB_2_CO ring with an exocyclic C=O bond (Figure 4, bottom). In this process, an imino-N atom of the amidinate group ligates to B1 center to form the silaoxadiborinanone **10**. The B–B bond length of 1.846(5) Å indicates a B–B single bond. Heating the toluene solution of **10** at 90 °C results in its conversion to **9**, which means **10** is an intermediate in the formation of **9**.

Main-group element-carbonyl species have attracted considerable attention in the past decades, mimicking their transition-metal counterparts in reactivity.^31,46^ For low-valent boron chemistry, a handful of boron carbonyl^31,47,48^ and diboron dicarbonyl species^49^ showing a transition-metal-like reactivity have been investigated. In addition, the diboryne carbonyl complex could also be isolated.^50^ The OC → B=B moiety in **9** represents an unprecedented example of diborene carbonyl.

### Density Functional Theory Calculations

We calculated the geometries of **2**, **6**, **8**, and **9** using density functional theory (DFT) at the BP86-D3(BJ)/def2-TZVP level and analyzed the electronic structures with a variety of methods. The optimized structures with the computed bond lengths and angles agree well with the experimental values (Figure S56). The boron atoms of the nonpolar B=B bonds in **2** and **6** which have NHSi ligands carry negative charges of −0.5*e* each, whereas the boron atoms of the polar B=B bonds in **8** and **9** possess significantly different charges (Table S21). The boron atom B2 in **8** which has a NHSi ligand exhibits an even higher negative charge of −0.6*e* and the B1 atom with a NHC ligand is nearly neutral. In contrast, the boron atom B1 in **9** which has also a NHC ligand has a positive charge of 0.2*e*, whereas the B2 atom with a CO ligand has a negative charge of −0.2*e*. Compounds **8** and **9** are particularly interesting because two different ligands are directly competing for the π electronic charge of the B=B bond. Both ligands NHSi and NHC are overall electron donors in **8** where the positive charge of NHC (0.3*e*) is smaller than that of NHSi (0.5*e*). The NHC ligand has a much higher positive charge in **9** of 0.7*e* where the CO ligand has a negative charge of −0.15*e*. The individual contribution of the σ donation and π backdonation is not obvious from the charge distribution, since the partial charges are the result of the total electron interaction. Note that the Si atoms in **2**, **6**, and **8** carry large positive charges of nearly +2. This is reasonable because they are bonded to three nitrogen atoms. The donor–acceptor interaction between boron and the ligands is analyzed in more detail with energy decomposition analysis with the combination of natural orbital for chemical valence (EDA-NOCV) method, whose results are presented and discussed below.

The calculated bond orders (Table S21) indicate a significant multiple bond character of the B1–B2 bond in all molecules in the order **2** > **6** > **8** > **9**. It is interesting to learn that the B2-CO bond in **9** has a significantly higher bond order (1.41) than the B1-NHC bond (1.09). This supports the suggestion that CO is a stronger π acceptor than NHC. Furthermore, the B1-NHC bond order in **8** is larger (1.16) than that of B2-NHSi (0.72), but the latter comparison deals with different bonds (B–C vs B–Si), whereas the same B–C bonds are involved in **9**. Note that the bond orders must not be directly identified with the degree of bond multiplicity. The polar bonds have always a smaller bond order for an electron-pair bond because the orbital overlap becomes smaller.^51^ Polar multiple bonds may have bond orders smaller than 1 due to the bond polarity. There is a frequent misunderstanding of covalent polar bonds caused by the suggestion of Pauling that there is a mixing of covalent and ionic interactions,^52,53^ which stems from the formalism of valence bond theory.^54^

**Figure 5:**
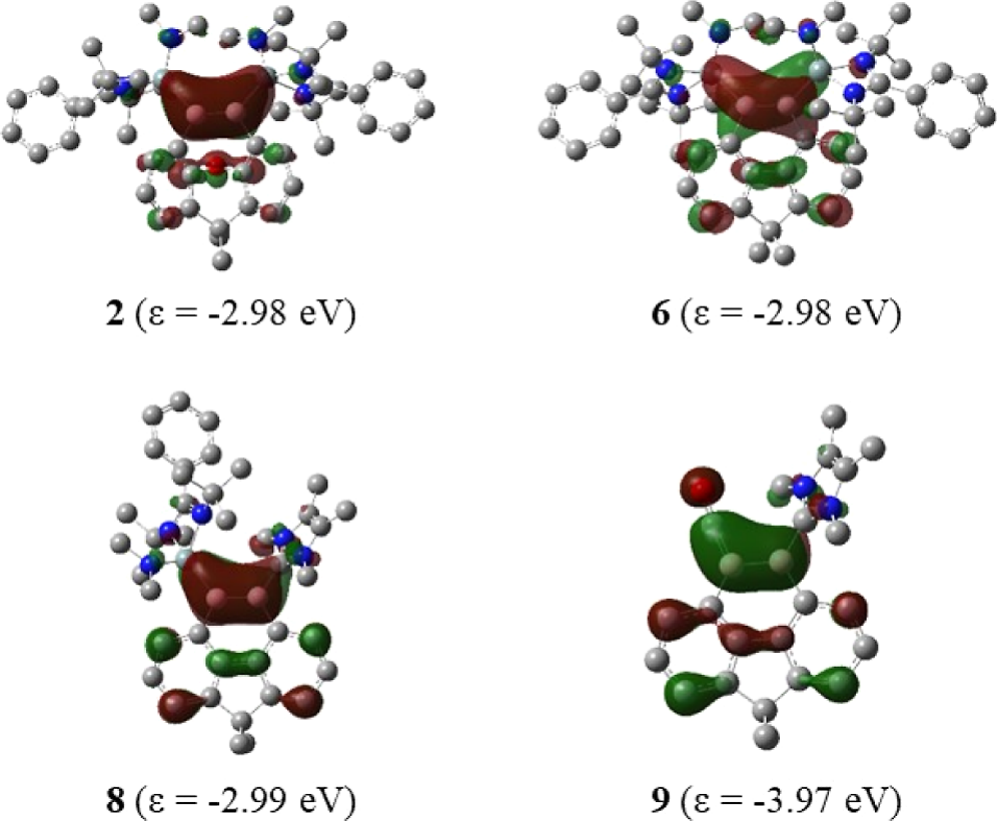
Plots of the HOMO of **2**, **6**, **8**, and **9** and their orbital energies at the BP86-D3(BJ)/def2-TZVP level.

The shape of the highest occupied molecular orbitals (HOMO) of the four molecules (Figure 5) is mainly a B–B π orbital that is somewhat delocalized. The nonpolar B–B π orbitals of **2** and **6**, and the HOMO of **8**, which are slightly polarized toward the NHSi ligand, have nearly the same eigenvalue. However, the B–B π orbital of **9** is much lower in energy, and it is clearly polarized toward the CO ligand and overlapping with the CO carbon atom. The polarity of the π HOMO agrees with the trend of the calculated charges at the boron atoms.

To investigate the aromatic character in the six-membered diboracyclic moieties of **6**, **8**, and **9**, we carried out Nucleus Independent Chemical Shift (NICS) calculations of the three six-membered rings where rings 1 and 2 are all-carbon moieties and ring 3 is the diboracyclic ring (Table 1). For comparison, NICS values for the all-carbon homologue **9-C** were also calculated where the B–L moieties of **9** (L = CO, NHC) are replaced by CH fragments. The calculated NICS (0) values at the center of rings 1 and 2 suggest a significant ring current between −9 and −10 ppm for **6**, **8**, and **9** that is typical for a benzene-like aromatic ring, whereas the NICS (0) values at the center of ring 3 have positive values. However, the NICS (0) values of **9-C** indicate that there is an intrinsic shift of the ring current toward rings 1 and 2 and a concomitant reduction in ring 3 (Table S22). More relevant for the aromaticity are the NICS (1) and NICS (−1) values above and below the ring (Table 1). The calculated values for ring 3 in **9-C** are 8 ppm lower than for rings 1 and 2, but they are still strongly negative with −21 ppm. The data suggest that the aromaticity of ring 3 is lower than in the rings 1 and 2, but it is still quite high. Accordingly, the NICS (1) and NICS (−1) values of ring 3 in **6**, **8**, and **9** are smaller than those of rings 1 and 2, but they are still negative. There are substantial aromatic characters in the diboracyclic rings with the order **6** > **8** > **9**. The smaller NICS (1) and NICS (−1) values for rings 3 of **6**, **8**, and **9** than that of **9-C** indicate that the incorporation of the B=B bond into the phenanthrene skeleton perturbs its π-electron delocalization, leading to the reduced aromaticity.

**Table 1:**
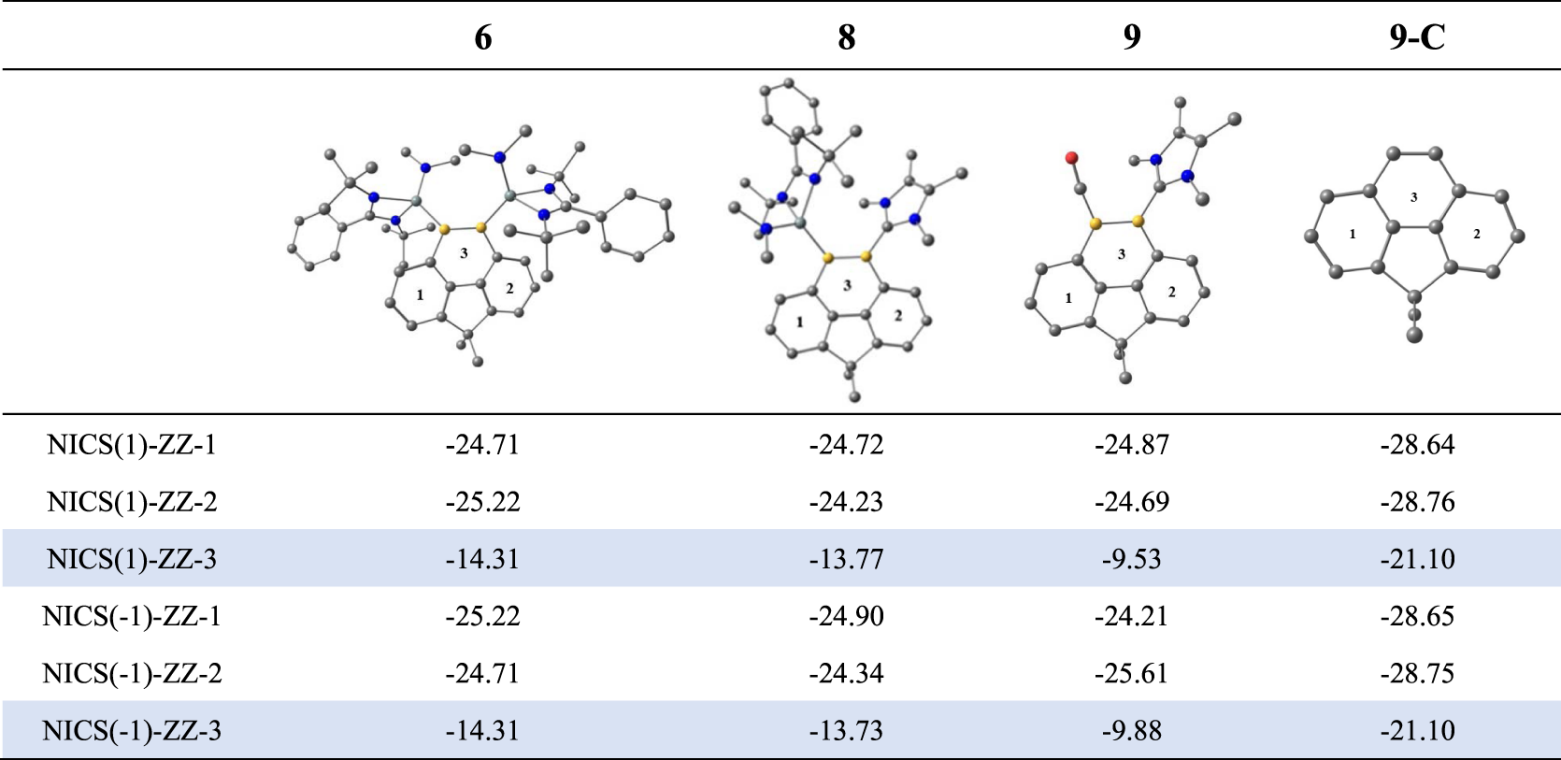
Calculated NICS(1)-ZZ and NICS(−1)-ZZ Values of **6**, **8**, and **9** and the All-Carbon Phenanthrene Analogue **9-C**

The different ligands (NHSi, NHC, and CO) show distinct substituent effects on the B=B bonds in **2**, **6**, **8**, and **9**. The competition between the three ligands for the π electrons of the B=B bond gives valuable information about the σ-donor/π-acceptor strength of the ligands in these compounds. The ligands NHSi, NHC, and CO are on equivalent positions, and the bonding analysis makes it possible to directly compare the donor–acceptor interactions with boron atoms when they are competing with each other. The numerical results of the EDA-NOCV calculations of **2**, **6**, **8**, and **9** are calculated (Table 2), where the interaction between the ligands NHSi, NHC, CO and the remaining diboracyclic moiety (“Rest”) are taken as fragments. The total interaction energies Δ*E*_int_ are quite large between −78.5 and −107.3 kcal/mol, which indicates strong boron-ligand bonds. Roughly half of the attractive contribution comes from the orbital (covalent) term Δ*E*_orb_ which is between 46 and 62% of the total attraction. The most interesting information comes from the breakdown of the orbital term Δ*E*_orb_ into pairwise contributions between the ligands L and the Rest fragment Δ*E*_orb(*n*)_. The strongest orbital interactions come from L → Rest σ donation Δ*E*_orb(1)_ of the lone-pair HOMO of L into the LUMO of Rest. The significantly weaker contribution Δ*E*_orb(2)_ is due to the L ← Rest π backdonation from the HOMO of Rest, which is the π orbital that has its largest extension at the B=B orbital.^55^ The nature of the orbital interactions can nicely be identified by inspection of the associated deformation densities and fragment orbitals (Figures S57–S60). There is a third and still weaker orbital term Δ*E*_orb(3)_, which comes in most cases from in-plane L ← Rest π_∥_ backdonation. Note that the relative strength of Δ*E*_orb(*n*)_ between CO and Rest in **9** differs significantly from the other ligand-Rest interactions. The L → Rest σ donation Δ*E*_orb(1)_ is only 50% of Δ*E*_orb_ when L = CO, whereas it is ∼75% for NHC and NHSi. In contrast, the total L ← Rest π backdonation is 44% of Δ*E*_orb_ when L = CO, whereas it is only ∼15% for NHC and NHSi. The strong π backdonation from the B=B bond to CO in **9** reduces its aromaticity of ring 3, which is in line with the results of NICS calculations. The NICS (1) and NICS (−1) values for ring 3 of **9** are significantly smaller than those of **6** and **8**. The remaining orbital interactions Δ*E*_orb(rest)_ come mainly from orbital relaxation within the fragments.

**Table 2:** EDA-NOCV Results of **2**, **6**, **8**, **9** at the BP86-D3(BJ)/TZ2P Level of Theory^a^

	orbital interaction	**2**	**6**	**8**		**9**	
		Fragments					
		NHSi + rest	NHSi + rest	NHSi + rest	NHC + rest	NHC + rest	CO + rest
Δ*E*_int_		–88.3	–84.3	–87.5	–97.3	–107.3	–78.5
Δ*E*_Pauli_		200.4	271.5	183.3	233.1	205.4	229.3
Δ*E*_disp_^b^		–27.4	–27.7	–26.0	–21.9	–10.4	–4.2
		(9.5%)	(7.8%)	(9.6%)	(6.6%)	(3.3%)	(1.3%)
Δ*E*_elstat_^b^		–117.3	–158.7	–109.2	–157.0	–152.7	–112.8
		(40.6%)	(44.6%)	(40.3%)	(47.5%)	(48.8%)	(36.6%)
Δ*E*_orb_^b^		–144.0	–169.3	–135.6	–151.6	–149.6	–190.8
		(49.9%)	(47.6%)	(50.1%)	(45.9%)	(47.9%)	(62.1%)
Δ*E*_orb(1)_^c^	L → rest σ donation	–110.0	–129.4	–103.2	–109.4	–113.7	–98.5
		(76.4%)	(76.3%)	(76.1%)	(72.2%)	(76.0%)	(51.6%)
Δ*E*_orb(2)_^c^	L ← rest π backdonation	–13.2	–23.1	–13.5	–15.1	–13.1	–50.7
		(9.2%)	(13.6%)	(10.0%)	(10.0%)	(8.8%)	(27.0%)
Δ*E*_orb(3)_^c^	L ← rest π′ backdonation	–6.1	–5.2	–5.7	–8.3	–6.7	–32.5
		(4.2%)	(3.1%)	(4.2%)	(5.5%)	(4.5%)	(17.0%)
Δ*E*_orb(rest)_^c^		–14.7	–12.0	–13.2	–18.8	–16.1	–9.1
		(10.2%)	(7.0%)	(9.7%)	(12.3%)	(10.7%)	(4.4%)

a Energy values are given in kcal/mol.

b The values in parentheses give the percentage contribution to the total attractive interactions Δ*E*_elstat_ + Δ*E*_orb_ + Δ*E*_disp_.

c The values in parentheses give the percentage contribution to the total orbital interactions Δ*E*_orb_.

The EDA-NOCV calculations suggest that the π-acceptor strength of the ligands has the order of CO ≫ NHC > NHSi. The boron-CO interaction in **9** is thus an example of the Dewar–Chatt–Duncanson (DCD) model for metal–ligand bonding, which was introduced by Dewar for the bonding in Zeises salt and generalized for transition-metal complexes by Chatt and Duncanson.^56,57^ The DCD approach is related to the frontier molecular orbital (FMO) model developed by Fukui^58,59^ and the orbital symmetry rules by Woodward and Hoffmann.^60^ The difference between the results of **9** and transition-metal (TM) carbonyl complexes is that the TM → CO π backdonation is usually much stronger and the TM ← CO σ donation is weaker,^61^ except in so-called nonclassical carbonyl complexes.^62^

We wondered whether compounds **2**, **6**, **8**, **9** may correctly be described in terms to dative interactions between closed-shell fragments L and Rest in the electronic singlet state. This holds in particular for **9** where the bonding situation of the CO group might be sketched with different resonance structures where neutral or charged open-shell fragments are bonded through electron-sharing interactions. To this end, we carried out EDA-NOCV calculations using L and Rest with different charges and electronic states (Tables S23–S28). It has been shown before that the best fragments give the lowest absolute values for the orbital interactions Δ*E*_orb_ because they change least during bond formation.^63−65^ The calculations using neutral L + Rest in the electronic singlet state give always the lowest Δ*E*_orb_ values. The chemical bonding is correctly described in terms of dative interactions.^66^

## Conclusions

In conclusion, the first silylene-stabilized dibenzodiboraoxepin **2** has been realized. Its high ring strain and elongated B=B bond facilitates the activation of P_4_ and NH_3_ at room temperature, furnishing compounds **3** and **4**, respectively. Thermal rearrangement of **2** generated a 9,10-dihydro-9,10-diboraphenanthrene derivative **5** through a bis(NHSi)-assisted deoxygenation process of the xanthene backbone. On a similar basis, the 9,10-diboraphenanthrene derivatives **6** and **8** could be synthesized by a selective and mild reductive deoxygenation of **2**. Utilizing the isoelectronic relationship between B=B bonds in diborene and C=C bonds in PAHs, a novel approach to generate diboron-containing PAHs has been established. Strikingly, the NHSi ligand in **8** is capable of further mediating the monodeoxygenation of CO_2_ to form the CO-stabilized 9,10-diboraphenanthrene derivative **9** via the isolable silaoxadiborinanone intermediate **10**. The OC → B=B moiety in **9** represents an unprecedented example of diborene carbonyl complex. Remarkably, the dibora-aromatics **6**, **8**, and **9** possess a high degree of aromaticity and distinct ligand effects (NHSi, NHC, and CO) on the B=B bonds, as confirmed by DFT calculations. Investigations on the redox and luminescent behavior of the 9,10-diboraphenanthrene derivatives are currently in progress.

## Supporting Information Available

Additional experimental details, materials and methods, spectroscopic data, and DFT calculations (PDF)
