# Supplementary material for: Silylene-Stabilized Neutral Dibora-Aromatics with a B=B Bond
Source: J Am Chem Soc. 2024 Jul 9;146(29):20458–67. doi: 10.1021/jacs.4c06579 (PMC11273343; doi:10.1021/jacs.4c06579)
Supplement: Supplementary file 1 — ja4c06579_si_001.pdf [file ja4c06579_si_001.pdf]

Supporting Information

Silylene-Stabilized Neutral Dibora-Aromatics with a B=B Bond

Jun Fan,[‡a] Jian Xu,[‡a] Qin Ma,[‡b] Shenglai Yao,[a] Lili Zhao,[b] Gernot Frenking*[b, c] and Matthias Driess*[a]

[a]Department of Chemistry: Metalorganics and Inorganic Materials, Technische Universität Berlin, Strasse des 17. Juni 115, Sekr. C2, 10623 Berlin (Germany)

[b]State Key Laboratory of Materials-Oriented Chemical Engineering, School of Chemistry and Molecular Engineering, Nanjing Tech University, Nanjing 211816 (China)

[c]Fachbereich Chemie, Philipps-Universität Marburg, 35032 Marburg (Germany)

Contents

A. Experimental Procedures 1

A1. General Considerations 1

A2. Single-Crystal X-ray Structure Determination 1

B. Synthesis and Characterization 3

C. X-ray Crystallographic Data 36

F. Theoretical Calculations 59

A. Experimental Procedures

A1. General Considerations

All experiments were carried out under dry oxygen-free nitrogen using standard Schlenk techniques or MBraun glove box fitted with a gas purification and recirculation unit. Solvents were dried by standard methods and freshly distilled prior to use. BBr_2(Xant)BBr_2 (Xant = 9,9-dimethyl-xanthene-4,5-diyl) and $LSiNMe_2$ (L = PhC(N^tBu)$_2$) were synthesized according to reported procedures.[1] The solution NMR spectra were recorded on Bruker Spectrometers AV 200, 400 and 500 with residual solvent signals as internal reference (^{1}H NMR: Benzene-d_6, 7.16 ppm, THF-d_8, 3.58 and 1.72 ppm, ^{13}C{^{1}H} NMR: Benzene-d_6, 128.06 ppm, THF-d_8, 67.21 and 25.31 ppm, *o*-$C_6D_4Cl_2$, 132.6, 130.29 and 127.5 ppm) and external standards (^{29}Si{^{1}H} NMR: $SiMe_4$, 0.0 ppm; 85% H_3PO_4 for ^{31}P{^{1}H} NMR; ^{11}B{^{1}H} NMR: $BF_3.Et_2O$). The following abbreviations were used to describe peak patterns when appropriate: br = broad, s = singlet, d = doublet, t = triplet, dd = doublet of doublets, m = multiplet. All ^{11}B{^{1}H} and ^{29}Si{^{1}H} NMR spectra were measured by using the borosilicate NMR tube. The broad feature in the ^{11}B{^{1}H} NMR spectra is a result of the probe. The broad feature in the ^{29}Si{^{1}H} NMR spectra is a result of quadrupole effect of adjacent boron atoms. High-resolution ESI-MS were measured on a Thermo Scientific LTQ orbitrap XL. IR spectra were measured with a Nicolet iS5 FT-IR-Spectrometer from the company Thermo. UV/Vis spectra were recorded on an Analytik Jena Specord S600 diode array spectrometer.

A2. Single-Crystal X-ray Structure Determination

Crystals were each mounted on a glass capillary in perfluorinated oil and measured in a cold N_2 flow. The data of all compounds were collected on an Oxford Diffraction SuperNova, Single source at offset, Atlas at 150 K (Cu-Kα radiation, λ = 1.54184 Å). The structures were solved by direct methods and refined on F^2 with the SHELX-2014[2] and Olex2[3] software package. In the molecular structure of compound **1**, one of the *tert*-butyl groups is disordered over two orientations with an approximate occupancy ratio of 0.58:0.42. In the molecular structure of compound **6**, one of the *tert*-butyl groups is disordered over two orientations with an approximate occupancy ratio of 0.72:0.28. In the molecular structure of compound **8**, one of the *tert*-butyl groups is disordered over two orientations with an approximate occupancy ratio of 0.68:0.32. The molecular

structures of compounds **6**, **8** and **9** each contain two independent molecules. In addition, the molecular structures of compounds **3** and **10** each contain three independent molecules. In the crystals of compounds **1**, **3**, **6**, **8**, **9** and **10** strongly disordered solvent molecules DME (**1** and **6**), and C_7H_8 (**3**, **8** and **9**) Et_2O (**10**) were treated using Solvent Masking in Olex2.

CCDC 2330334 (for **1**), 2330335 (for **2**), 2330336 (for **5**), 2330337 (for **4**), 2330338 (for **3**), 2353546 (for **6**), 2353547 (for **7**), 2353548 (for **8**), 2353549 (for **9**) and 2353550 (for **10**) contain the supplementary crystallographic data for this paper. These data can be obtained free of charge from The Cambridge Crystallographic Data Centre via www.ccdc.cam.ac.uk/structures/

B. Synthesis and Characterization

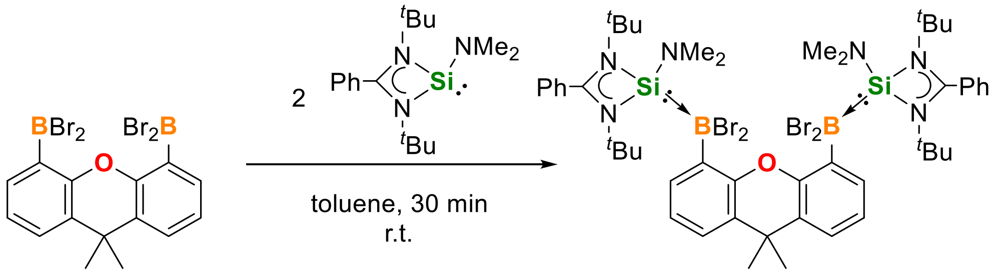

BBr$_2$(Xant)BBr$_2$ **1**

Synthesis of compound 1. To the mixture of **BBr$_2$(Xant)BBr$_2$** (550 mg, 1 mmol) and LSiNMe$_2$ (L = PhC(N^{t}Bu)$_2$) (606 mg, 2 mmol) in a 100 mL Schlenk flask was added 60 mL toluene at room temperature under stirring. After stirring 30 min, a white precipitate formed slowly. The white precipitate was separated by filtration and dried under vacuum to afford compound **1** as a white powder (982 mg, 85% isolated yields). Colorless block crystals suitable for X-ray diffraction analysis were obtained from a concentrated DME solution at 0 °C.

M.p.: 154.8 °C.

^{1}H NMR (500 MHz, Benzene-*d*$_6$) δ/ppm = 7.93 (d, *J* = 8.7 Hz, 2H, Ar-*H*), 7.83 (d, *J* = 7.8 Hz, 2H, Ar-*H*), 7.36 (d, *J* = 6.1 Hz, 2H, Ar-*H*), 7.11 (t, *J* = 7.5 Hz, 2H, Ar-*H*), 6.93 (t, *J* = 7.5 Hz, 2H, Ar-*H*), 6.84 (t, *J* = 7.4 Hz, 2H, Ar-*H*), 6.80 – 6.73 (m, 4H, Ar-*H*), 2.80 (s, 12H N(C*H*$_3$)$_2$), 1.60 (s, 6H, C(C*H*$_3$)$_2$), 1.12 (s, 36H, C(C*H*$_3$)$_3$).

^{13}C{^{1}H} NMR (50 MHz, Benzene-*d*$_6$) δ/ppm = 176.72 (s, N*C*N), 156.70, 134.07, 131.39, 131.01, 130.70, 130.58, 128.58, 128.43, 127.97, 127.79, 124.31, 120.80 (s, Ar-*C*), 54.83 (s, N*C*(CH$_3$)$_3$), 39.37 (s, N(*C*H$_3$)$_2$), 34.80 (s, *C*(CH$_3$)$_2$, 32.54 (s, C(*C*H$_3$)$_2$), 31.60 (s, C(*C*H$_3$)$_2$).

^{11}B{^{1}H} NMR (160 MHz, Benzene-*d*$_6$) δ/ppm = -8.45

^{29}Si{^{1}H} NMR (99 MHz,) δ/ppm = -10.27

HR-MS (ESI): (m/z) calcd for [M-Br]$^+$ ($C_{49}H_{70}B_2N_6O_1Si_2Br_3^+$) 1077.2839; Found: 1077.2852

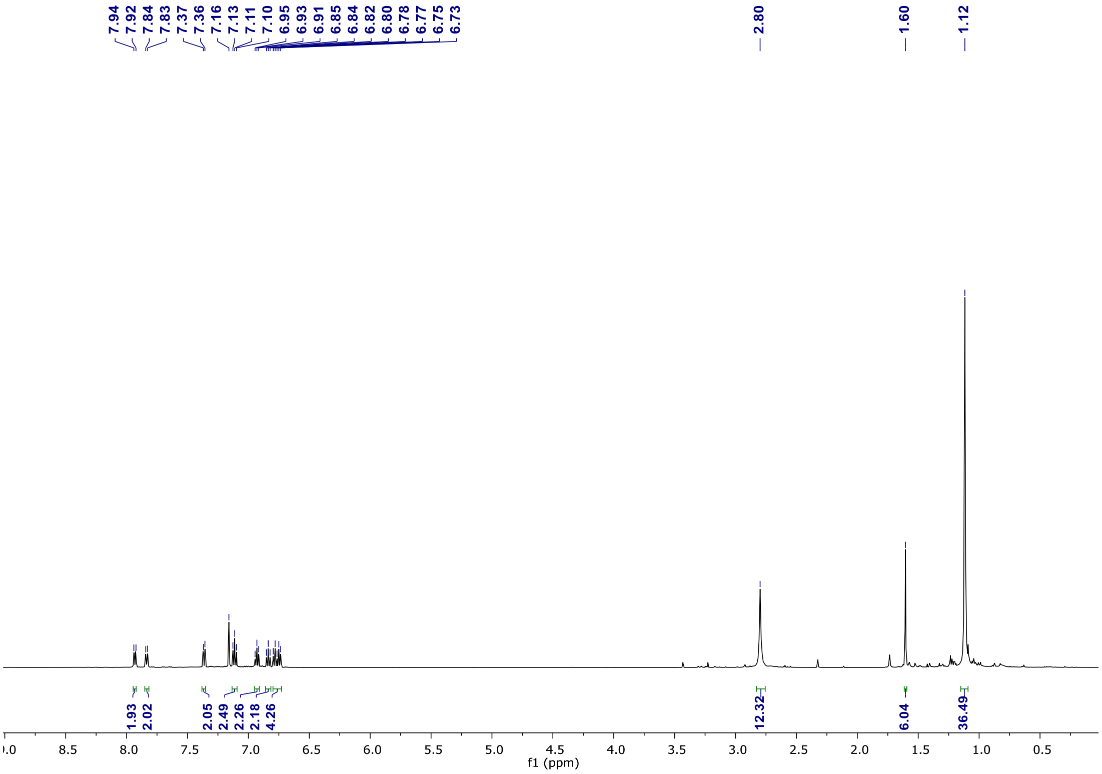

Figure S1. ^{1}H NMR spectrum of **1** in benzene-*d*$_6$.

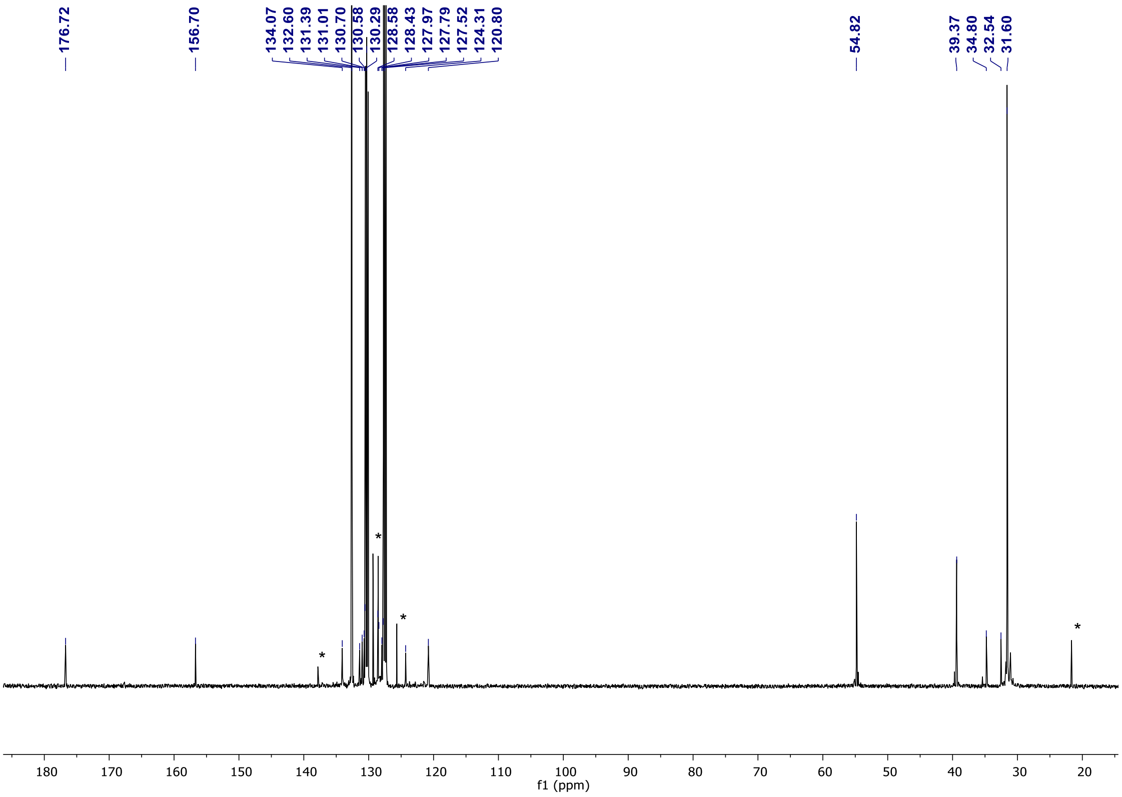

Figure S2. $^{13}C\{^{1}H\}$ NMR spectrum of **1** in *o*-$C_6D_4Cl_2$. * is solvent (Toluene).

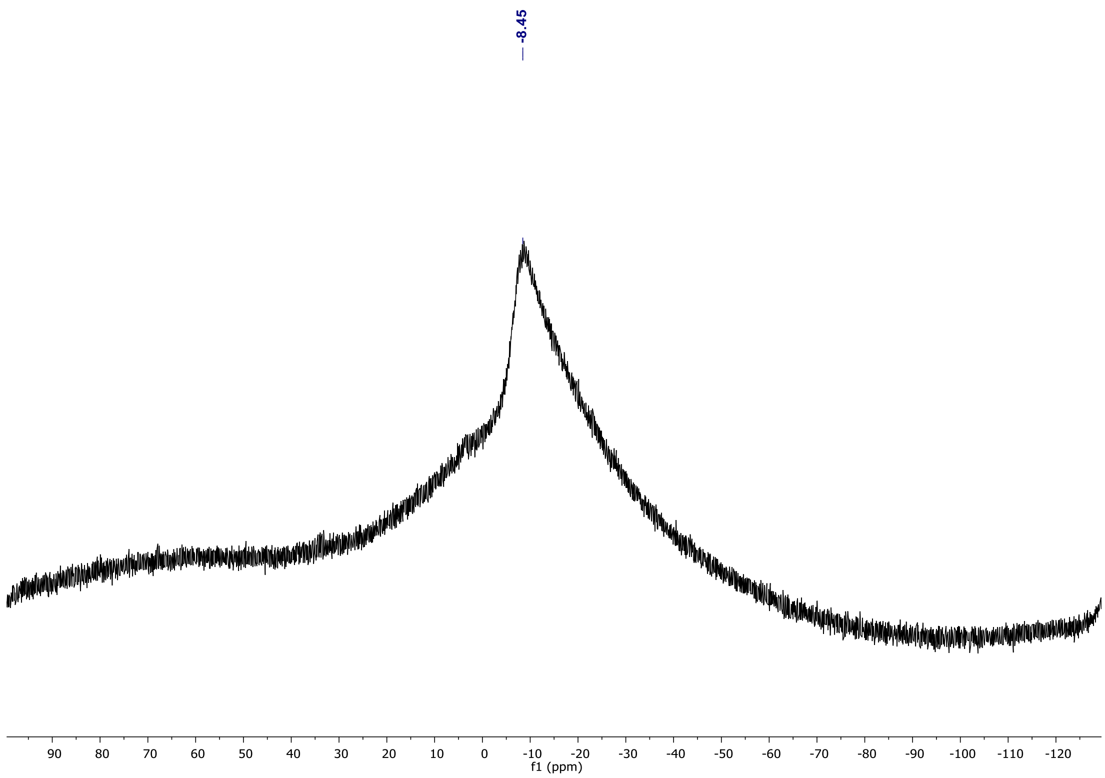

Figure S3. $^{11}B\{^1H\}$ NMR spectrum of **1** in benzene-d_6.

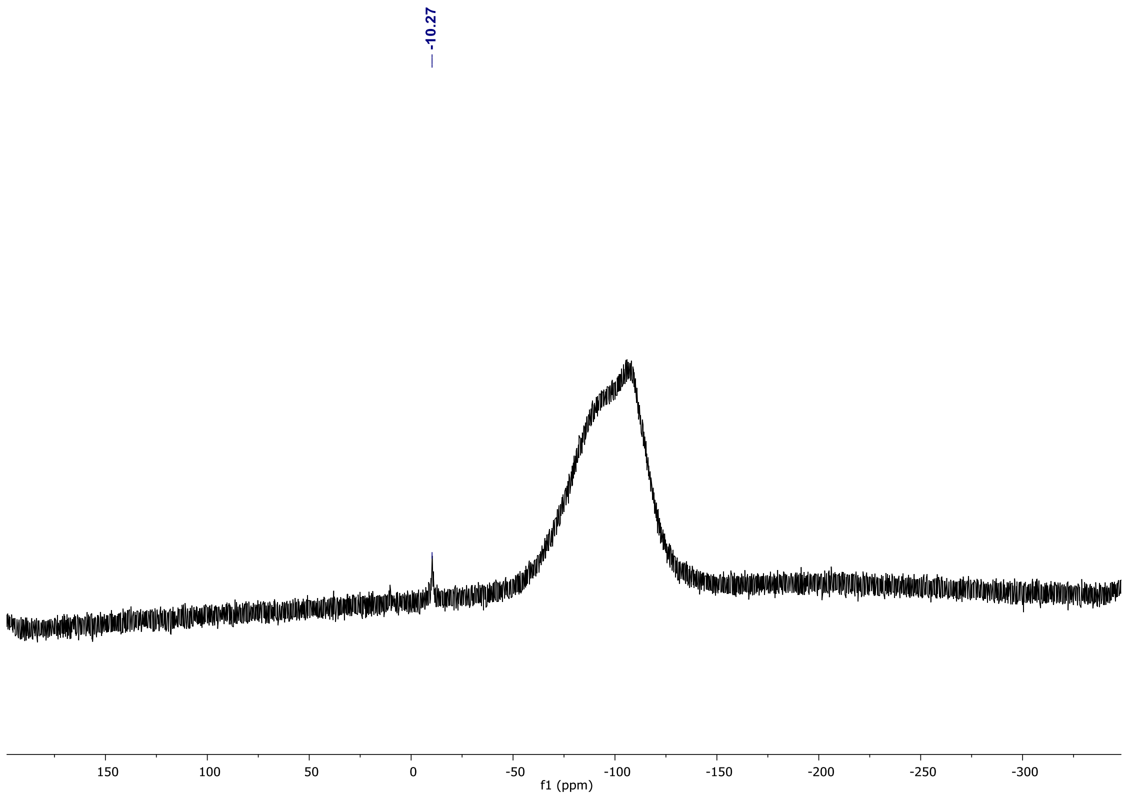

Figure S4. $^{29}Si\{^1H\}$ NMR spectrum of **1** in *o*-$C_6D_4Cl_2$.

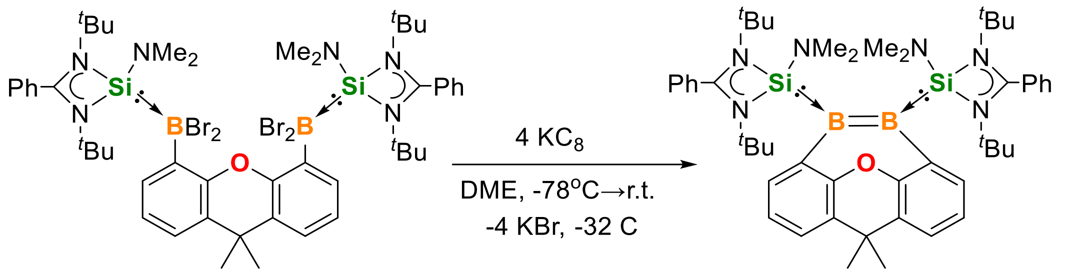

Synthesis of compound 2. To a mixture of **1** (2.31 g, 2 mmol) and KC_8 (1.08 g, 8 mmol) in a 100 mL Schlenk flask was added 50 mL DME at -78 °C under stirring. The color of the mixture changed from colorless to red immediately. The mixture was allowed to warm up to room temperature and stirred for 12 h. The dark red mixture was filtered and the residue was washed with THF (10 mL x 3). The all volatiles were removed and recrystallization from THF at 4 °C to give compound **2** as red crystals (1.17 g, 70% isolated yield). Red crystals suitable for X-ray diffraction analysis were obtained from a toluene solution at 4 °C.

M.p.: 121.4 °C (dec.).

^{1}H NMR (500 MHz, Benzene-d_6) δ/ppm = 7.67 (m, 4H, Ar-*H*), 7.18 (d, *J* = 1.2 Hz, 2H, Ar-*H*), 6.98 – 6.95 (m, 2H, Ar-*H*), 6.91 (m, 6H, Ar-*H*), 6.83 (t, *J* = 7.3 Hz, 2H, Ar-*H*), 3.20 (s, 12H, N(C*H*$_3$)$_2$), 2.63 (s, 3H, C(C*H*$_3$)$_2$), 1.92 (s, 3H, C(C*H*$_3$)$_2$), 1.20 (s, 18H, C(C*H*$_3$)$_3$), 1.10 (s, 18H, C(C*H*$_3$)$_3$).

^{13}C{^{1}H}NMR (101 MHz, THF-d_8) δ/ppm = 174.35 (s, N*C*N), 161.52, 148.01, 133.22, 130.91, 129.67, 129.56, 129.43, 128.79, 128.46, 118.47, 118.32(s, Ar-*C*), 54.28 (s, N*C*(CH$_3$)$_3$), 54.08(s, N*C*(CH$_3$)$_3$), 39.88 (s, N(*C*H$_3$)$_2$), 31.61 (s, *C*(CH$_3$)$_2$, 31.09 (s, *C*(CH$_3$)$_2$, 29.17 (s, C(*C*H$_3$)$_2$), 28.44 (s, C(*C*H$_3$)$_2$).

^{11}B{^{1}H} NMR (160 MHz, THF-d_8) δ/ppm = 25.78.

^{29}Si{^{1}H} NMR (99 MHz, THF-d_8) δ/ppm = 16.48.

HR-MS (ESI): (m/z) calcd for $[M+H]^+$ ($C_{49}H_{71}B_2N_6O_1Si_2^+$): 837.5409; Found: 837.5426

UV-Vis (THF), λ_{max}: 403, 486 nm.

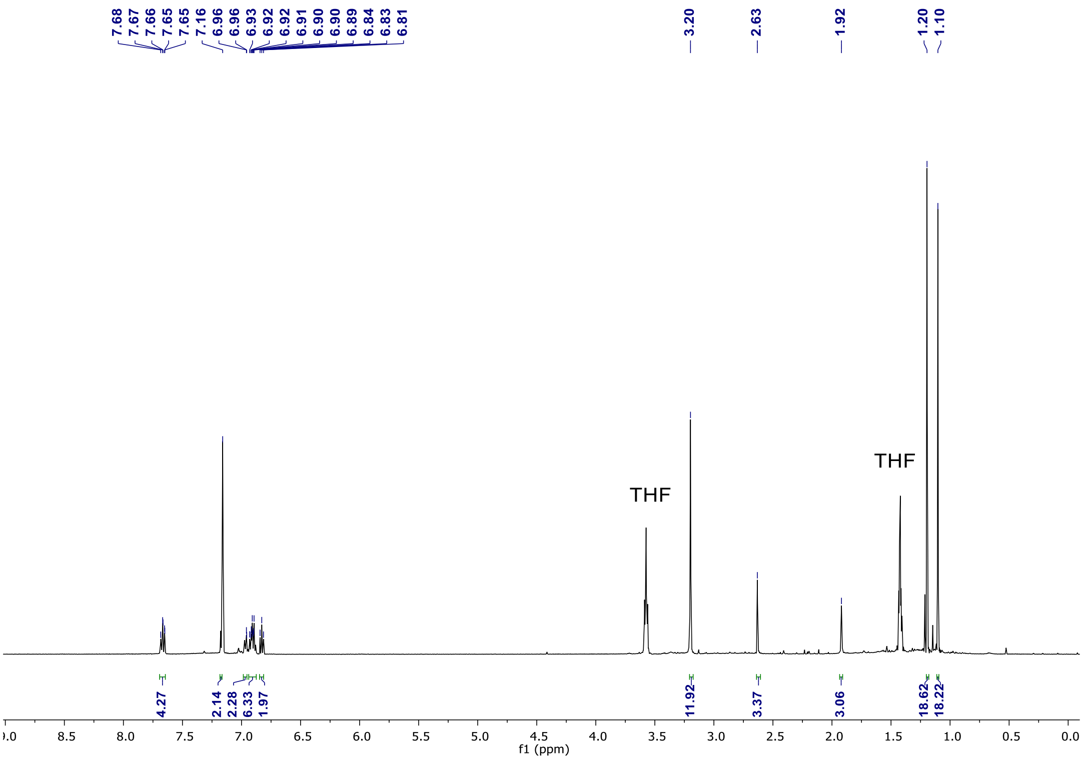

Figure S5. ^{1}H NMR spectrum of **2** in benzene-d_6.

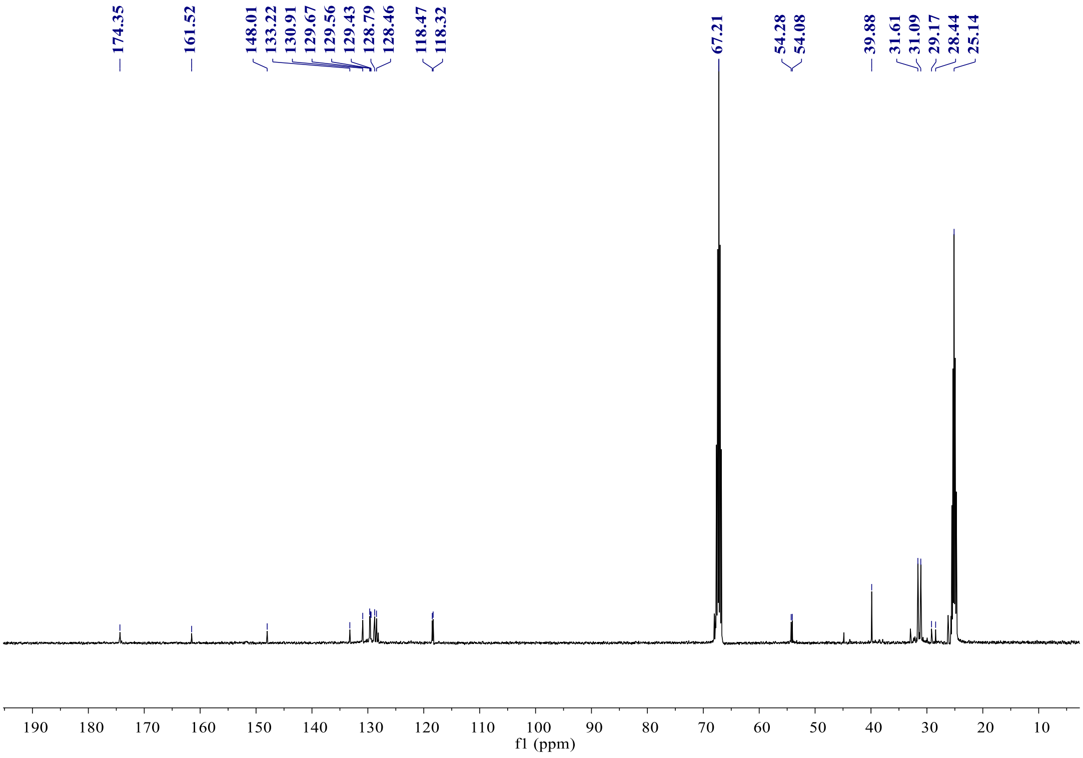

Figure S6. $^{13}C\{^{1}H\}$ NMR spectrum of **2** in THF-d_8.

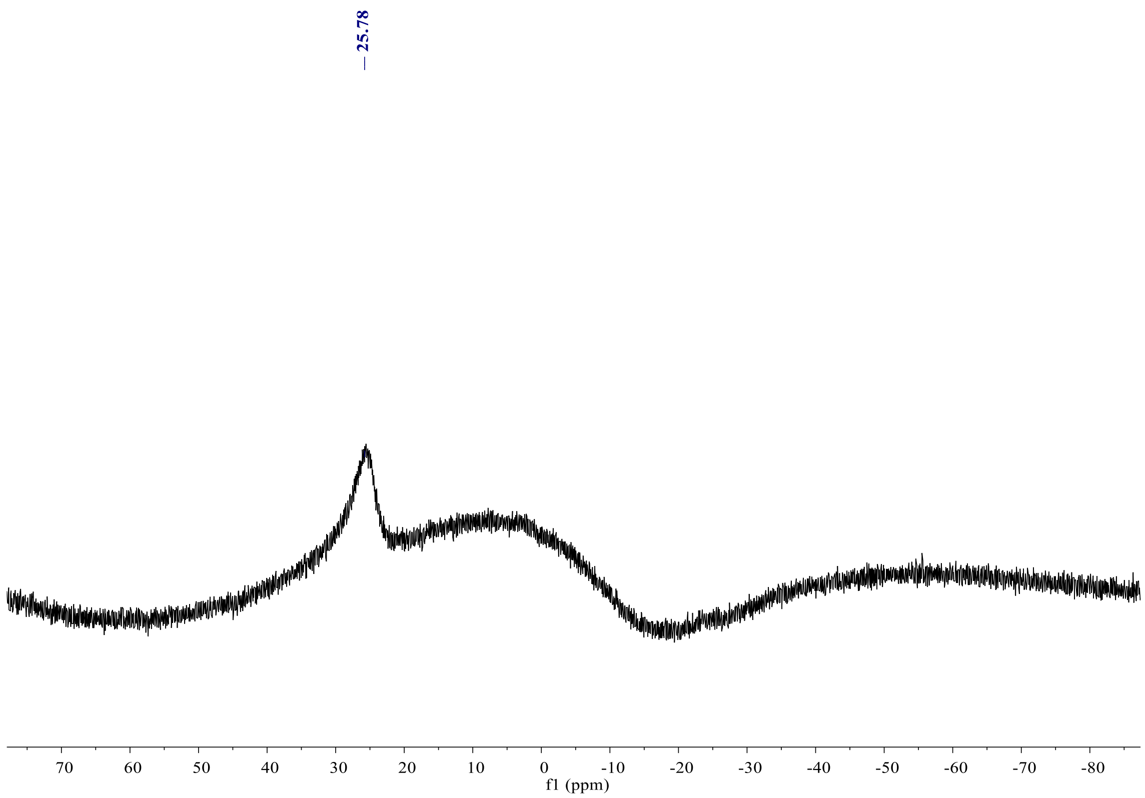

Figure S7. $^{11}B\{^{1}H\}$ NMR spectrum of **2** in benzene-d_6.

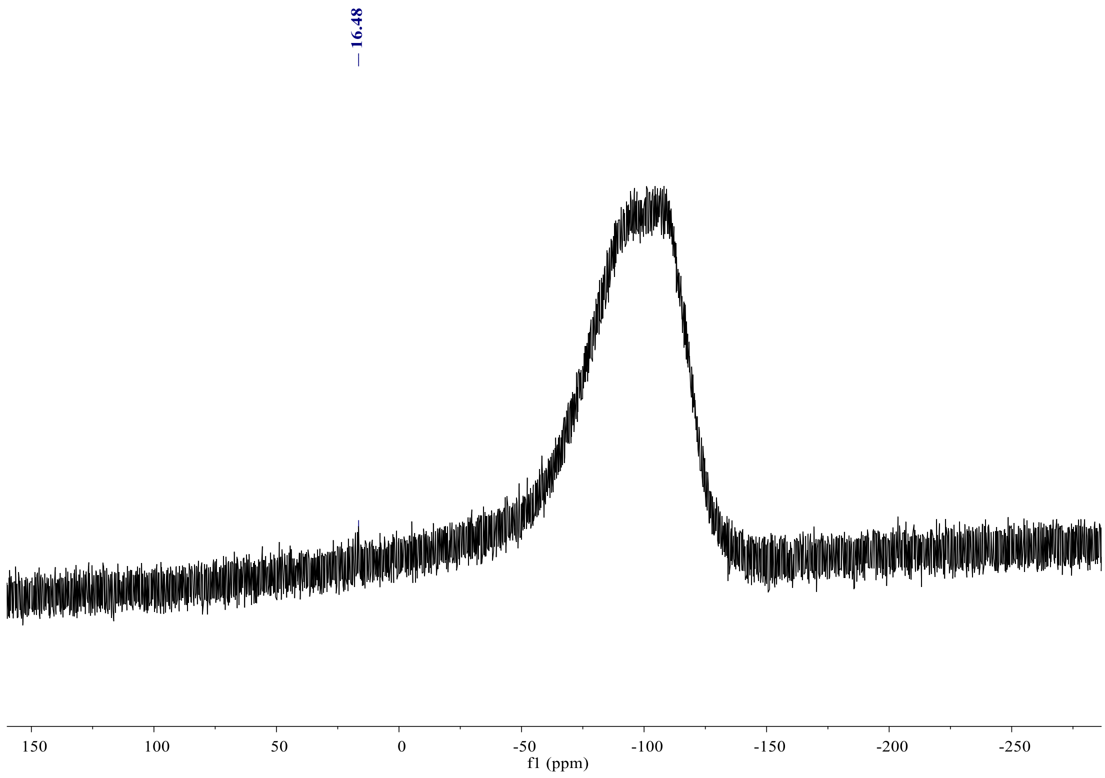

Figure S8. $^{29}Si\{^{1}H\}$ NMR spectrum of **2** in THF-d_8.

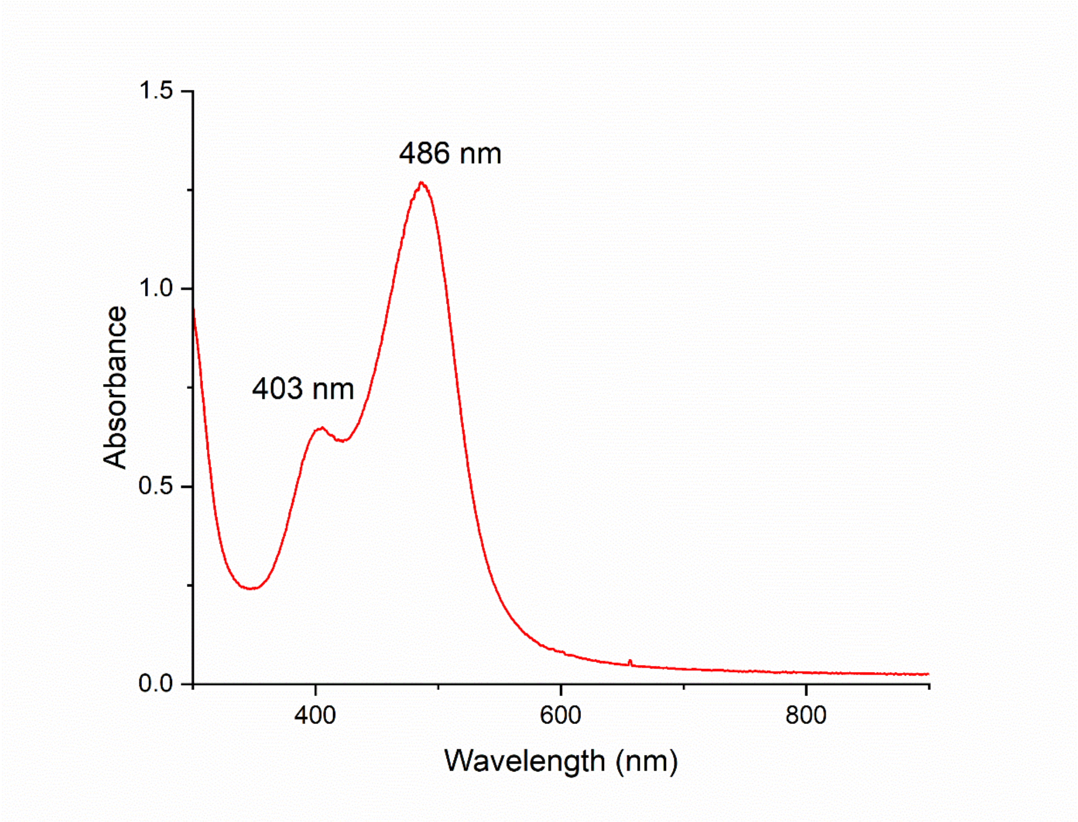

Figure S9. UV/Vis spectrum of compound **2** (RT, THF).

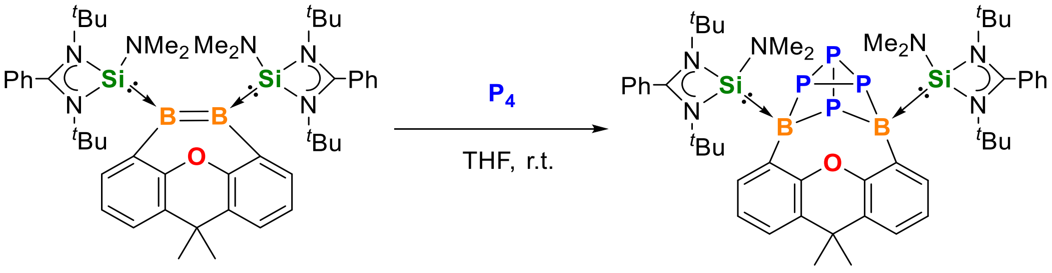

2 **3**

Synthesis of compound 3. To a mixture of compound **2** (418 mg, 0.5 mmol) and P_4 (62 mg, 0.5 mmol) in a 50 mL Schlenk flask was added 25 mL THF at room temperature under stirring. The color of the mixture changed from red to yellow immediately. After 15 min, the all volatiles were removed and dried under vacuum to afford compound **3** as a yellow powder (456 mg, 95% isolated yield). Yellow crystals suitable for X-ray diffraction analysis were obtained from a toluene/Et_2O solution at 4 °C for two weeks.

M.p.: 159.1 ^{o}C.

^{1}H NMR (500 MHz, THF-d_8) δ/ppm = 8.03 (d, J = 7.5 Hz, 2H, Ar-H), 7.63 (d, J = 7.4 Hz, 2H, Ar-H), 7.52 (m, , 6H, Ar-H), 7.44 (t, J = 8.0 Hz, 2H, Ar-H), 6.81 (d, J = 8.8 Hz, 2H, Ar-H), 6.77 (t, J = 7.4 Hz, 2H, Ar-H), 3.12 (s, 12H, N(CH_3)$_2$), 1.55 (s, 3H, C(CH_3)$_2$), 1.38 (s, 3H, C(CH_3)$_2$), 1.30 (s, 18H, C(CH_3)$_3$), 1.27 (s, 18H, C(CH_3)$_3$).

^{13}C NMR (101 MHz, THF-d_8) δ/ppm = 176.15 (s, NCN), 162.86, 141.26, 131.99, 131.25, 130.76, 129.99, 128.50, 128.27, 122.21, 120.49 (s, Ar-C), 55.01 (s, NC(CH$_3$)$_3$), 54.56 (s, NC(CH$_3$)$_3$), 40.11(s, N(CH$_3$)$_2$), 38.61 (s, C(CH$_3$)$_2$, 31.63 (s, NC(CH$_3$)$_3$, 31.35 (s, NC(CH$_3$)$_3$, 27.03 (s, C(CH$_3$)$_2$).

^{11}B{^{1}H} NMR (160 MHz, Benzene-d_6) δ/ppm = -15.64.

^{31}P{^{1}H} NMR (202 MHz, THF-d_8) δ/ppm = 144.95 (br, PB$_2$), -85.80 (br, PBP$_2$), -179.80 (br, PP$_3$).

^{29}Si{^{1}H} NMR (99 MHz, THF-d_8) δ/ppm = 23.99.

HR-MS (ESI): (m/z) calcd for $[M+H]^+$ ($C_{49}H_{71}B_2N_6OP_4Si_2^+$): 961.4359; Found: 961.4391.

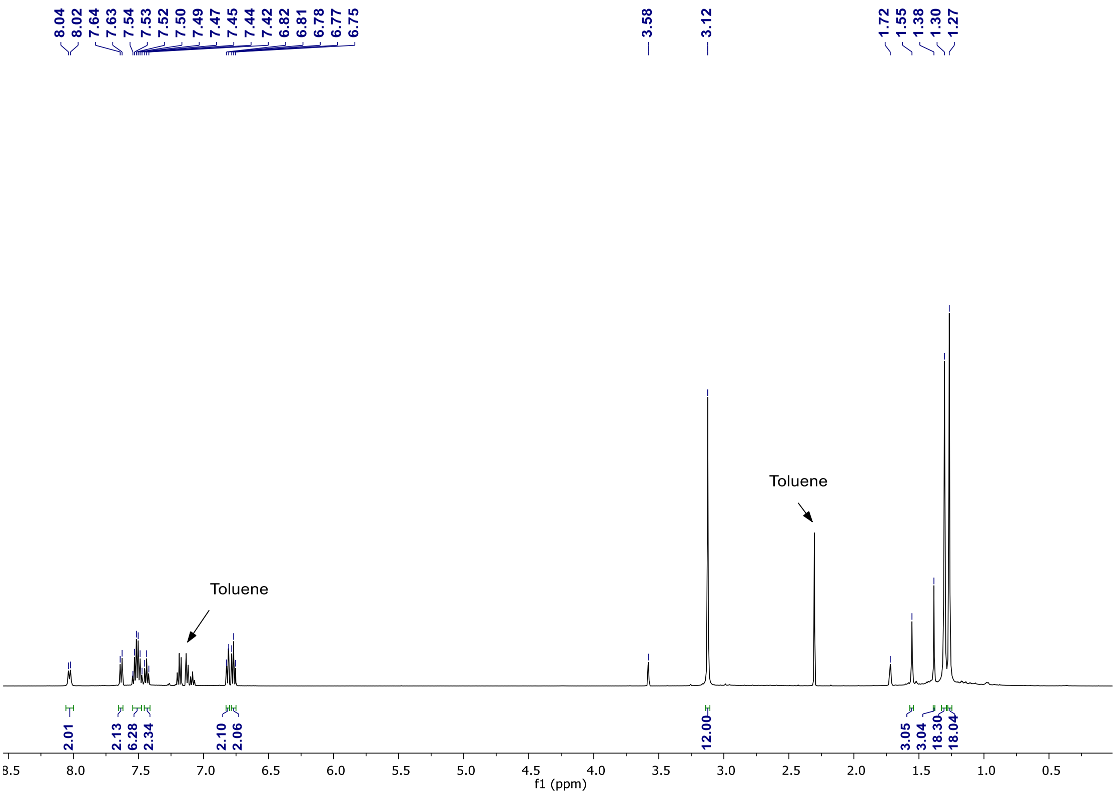

Figure S10. ^{1}H NMR spectrum of **3** in THF-d_8.

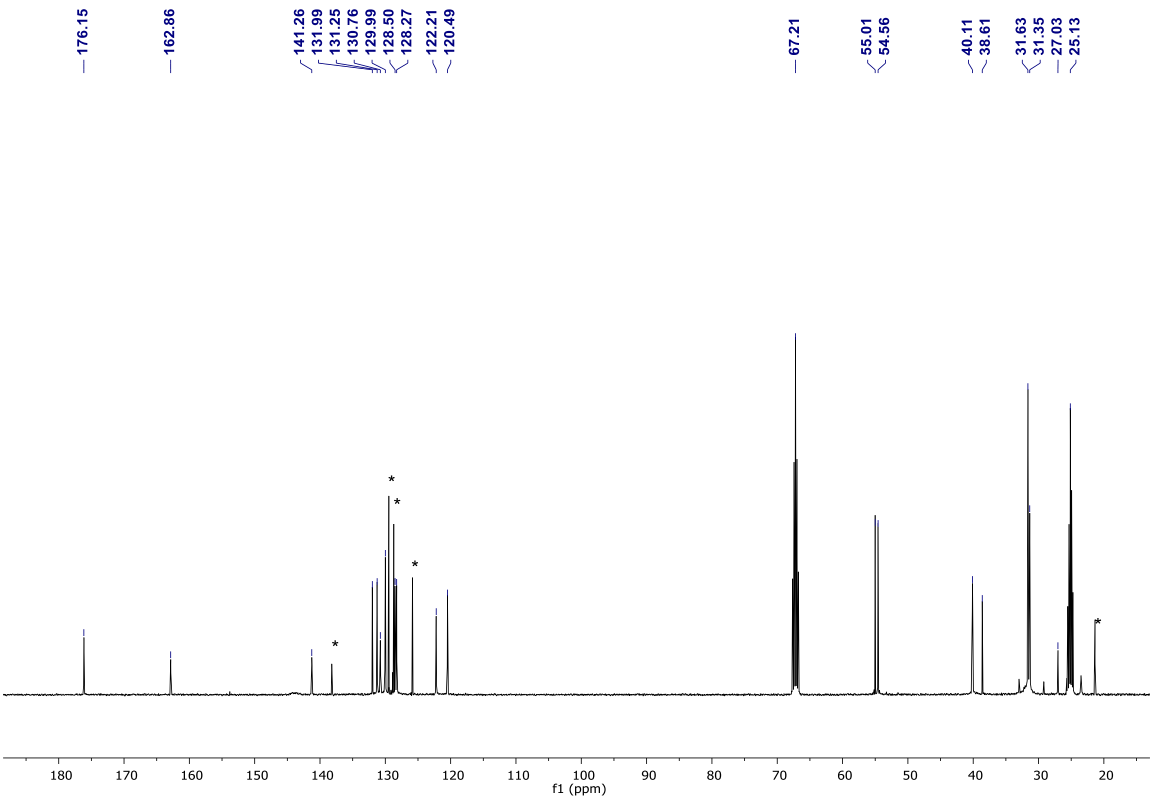

Figure S11. $^{13}C\{^{1}H\}$ NMR spectrum of **3** in THF-d_8.* is solvent (Toluene).

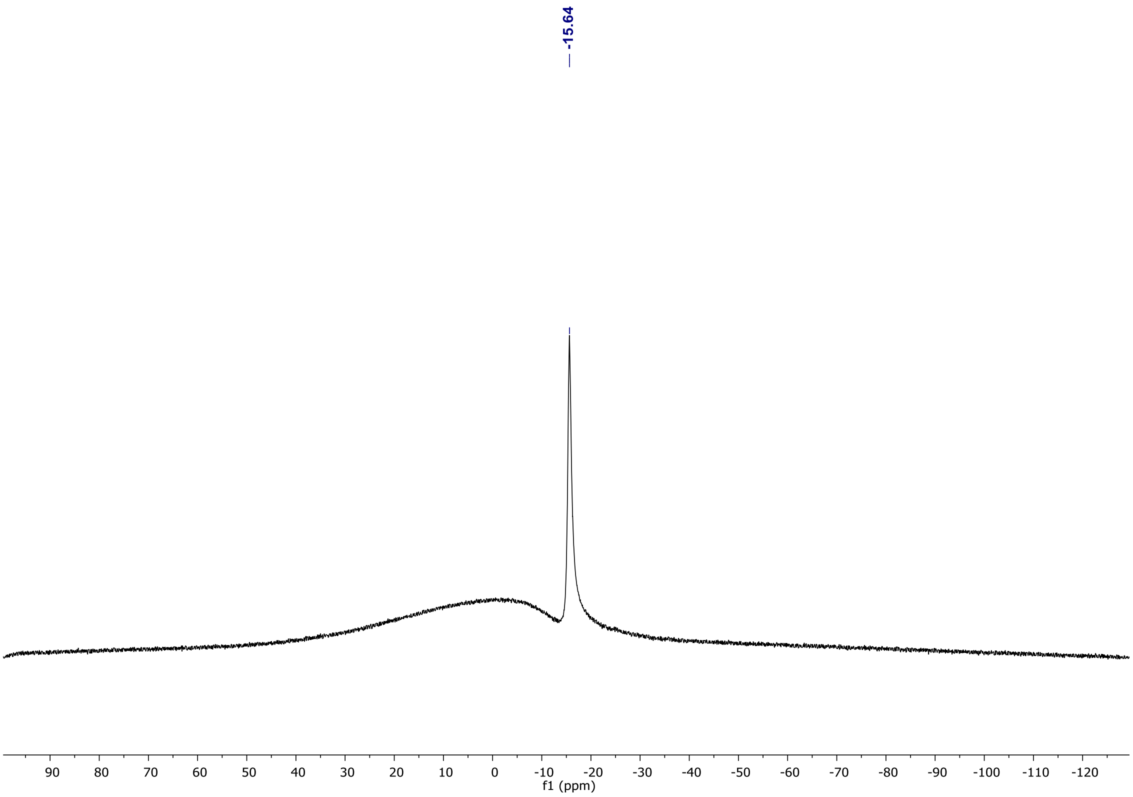

Figure S12. $^{11}B\{^{1}H\}$ NMR spectrum of **3** in THF-d_8.

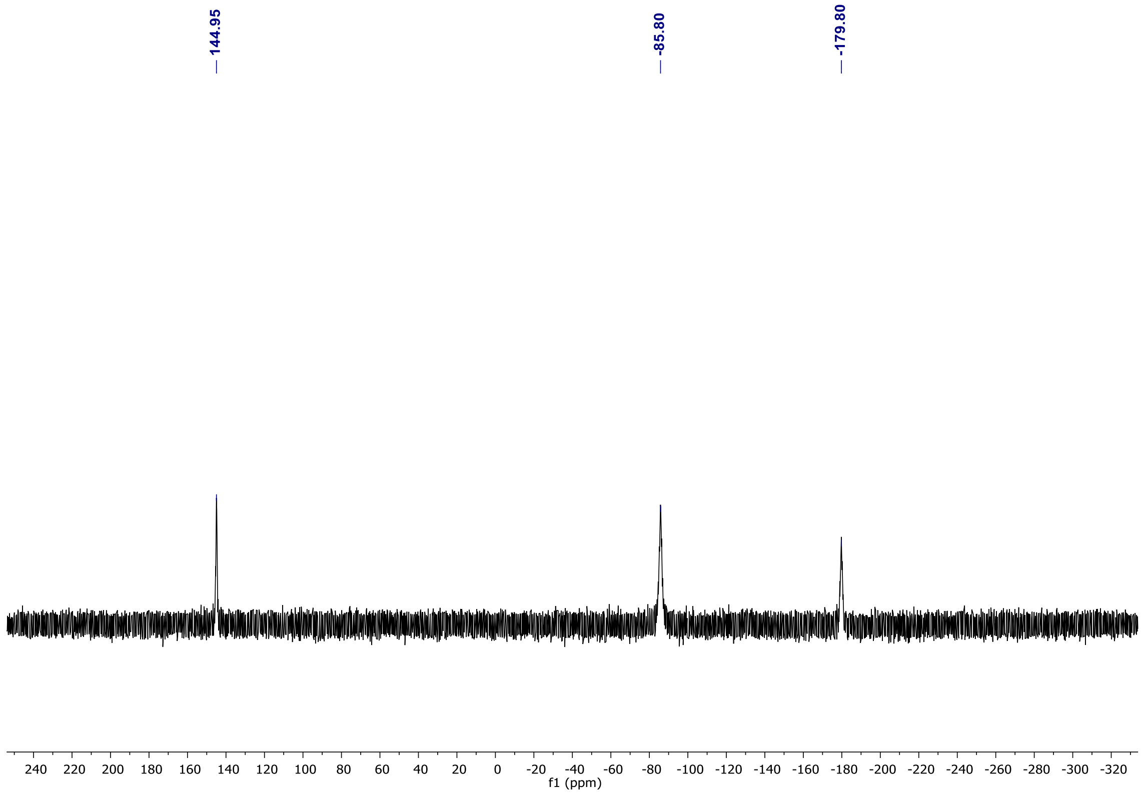

Figure S13. $^{31}P\{^{1}H\}$ NMR spectrum of **3** in THF-d_8.

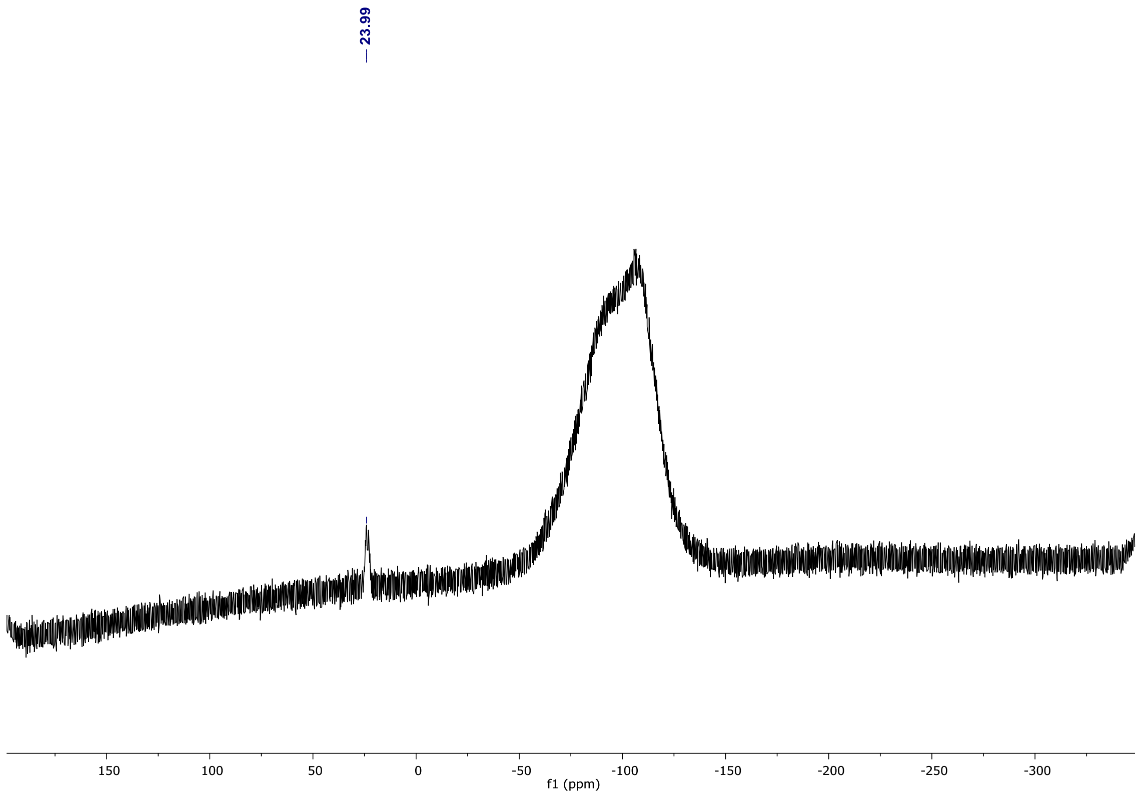

Figure S14. $^{29}Si\{^{1}H\}$ NMR spectrum of **3** in THF-d_8.

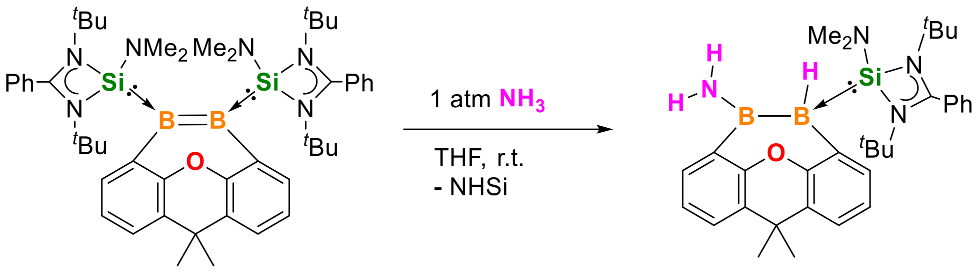

2 **4**

Synthesis of compound 4. After three freeze-pumpthaw cycles, the THF solution of compound **2** (418 mg, 0.5 mmol) in a 100 mL Schlenk flask was subjected to 1 atm NH_3 at room temperature and stirred for 12 h. The color of the mixture changed from red to colorless slowly. All volatiles were then removed and 50 mL hexane was added. The mixture was filtered and the residue was washed with hexane (10 mL) again. Finally, the all volatiles were removed and dried under vacuum to give compound **4** as a white powder (178 mg, 65% isolated yield). Colorless crystals suitable for X-ray diffraction analysis were obtained from a hexane solution at 4 °C.

M.p.: 202.5 °C.

^{1}H NMR (500 MHz, THF-d_8) δ/ppm = 7.54 (m, 1H, Ar-*H*), 7.47 (m, 3H, Ar-*H*), 7.39 (m, 1H, Ar-*H*), 6.98 (dd, *J* = 7.3, 1.2 Hz, 1H, Ar-*H*), 6.92 (m, 1.2 Hz, 2H, Ar-*H*), 6.72 (t, *J* = 7.3 Hz, 1H, Ar-*H*), 6.67 (dd, *J* = 7.4, 1.3 Hz, 1H, Ar-*H*), 6.48 (t, *J* = 7.3 Hz, 1H, Ar-*H*), 4.80 (s, 1H, NH_2), 4.17 (s, 1H, NH_2), 2.88 (s, 6H, N(CH_3)$_2$), 2.09 (s, 3H, C(CH_3)$_2$), 1.59 (s, 3H, C(CH_3)$_2$), 1.24 (s, 9H, C(CH_3)$_3$), 1.03 (s, 9H, C(CH_3)$_3$).

^{13}C NMR (126 MHz, THF-d_8) δ/ppm = 173.76 (s, N*C*N), 170.37, 166.83, 147.22, 145.09, 134.17, 131.93, 131.01, 130.32, 129.28, 128.49, 128.43, 127.58, 124.15, 122.61, 119.53 (s, Ar-*C*), 54.36 (s, N*C*(CH_3)$_3$), 53.79 (s, N*C*(CH_3)$_3$), 43.13 (s, *C*(CH_3)$_2$), 38.56 (s, N(*C*H_3)$_2$), 31.44 (s, NC(*C*H_3)$_3$, 30.46 (s, NC(*C*H_3)$_3$, 27.63 (s, C(*C*H_3)$_2$), 23.71(s, C(*C*H_3)$_2$).

^{11}B NMR (160 MHz, THF-d_8) δ/ppm = 52.06 (*B*NH_2), -34.81 (d, J_{B-H} = 80.4 Hz) (*B*H).

^{29}Si{^{1}H} NMR (99 MHz, THF-d_8) δ/ppm = 20.22.

IR (cm^{-1}): 3485.78 (w, N-H), 3397.86 (w, N-H), 2266.09 (m, B-H).

HR-MS(LIFDI) : (m/z) calcd for [M-H]$^+$ ($C_{32}H_{43}B_2N_4O_1Si_1^+$) 549.3387; Found:549.3397

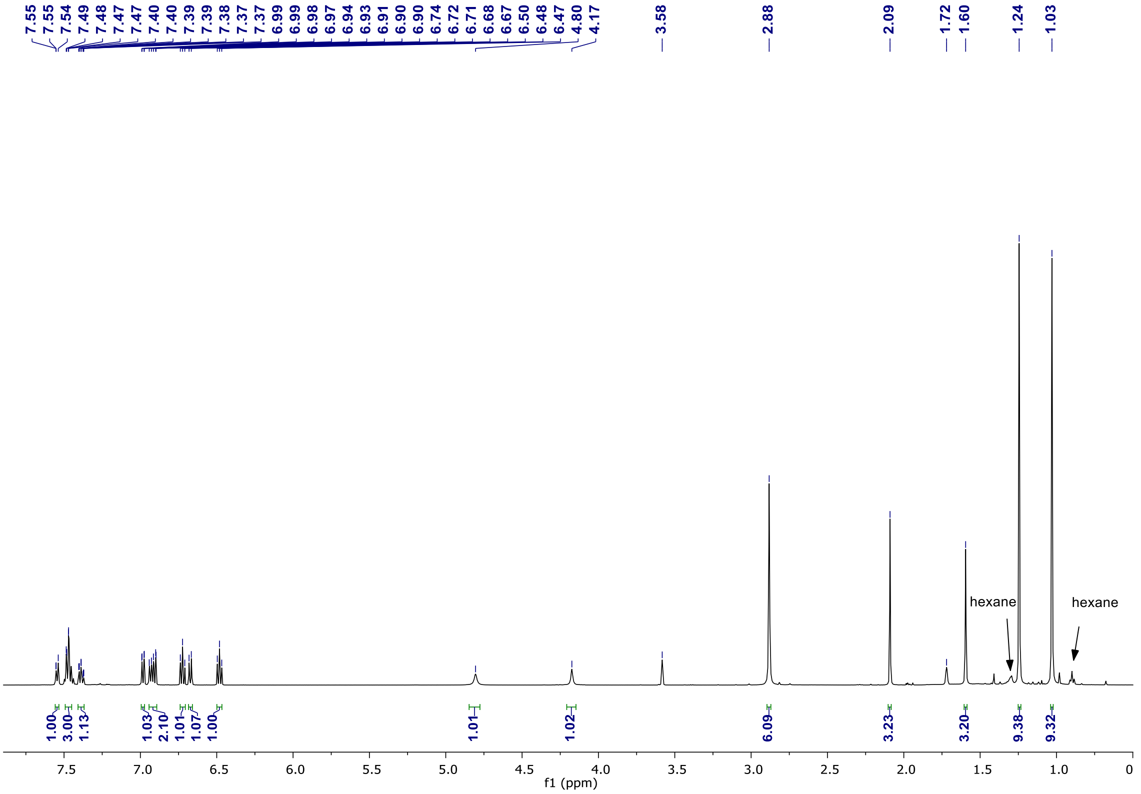

Figure S15. ^{1}H NMR spectrum of **4** in THF-d_8.

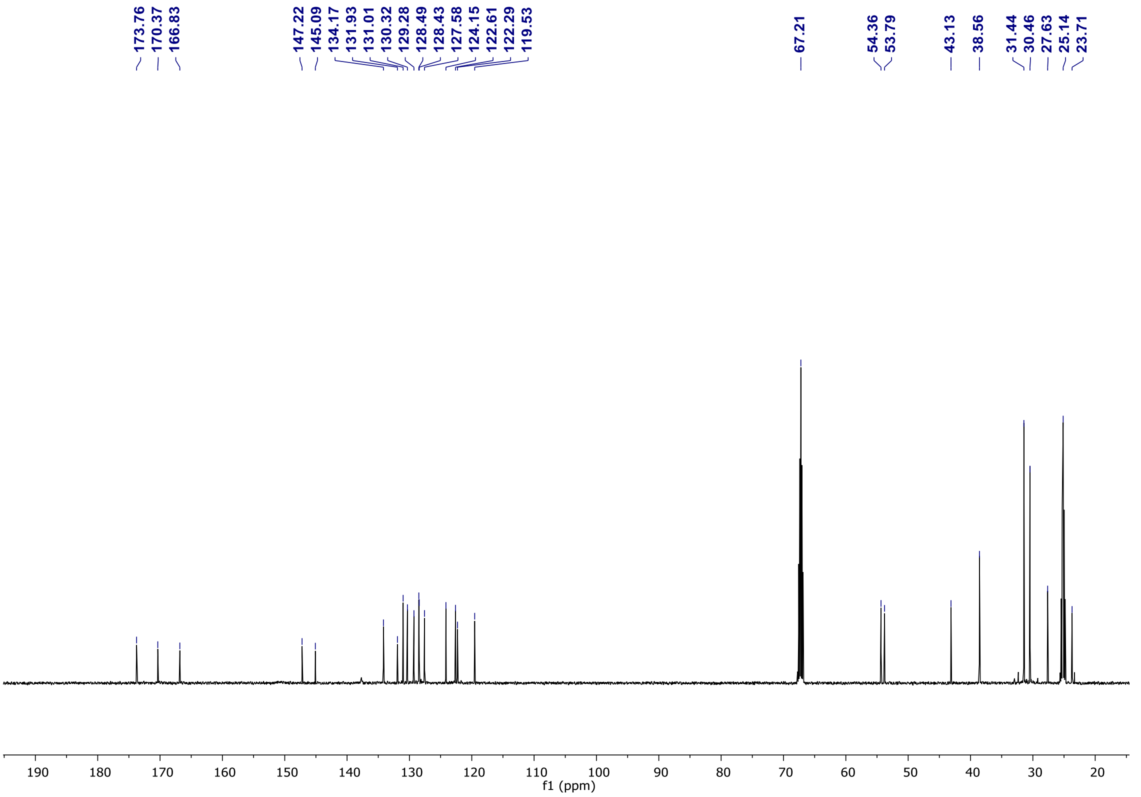

Figure S16. $^{13}C\{^{1}H\}$ NMR spectrum of **4** in THF-d_8.

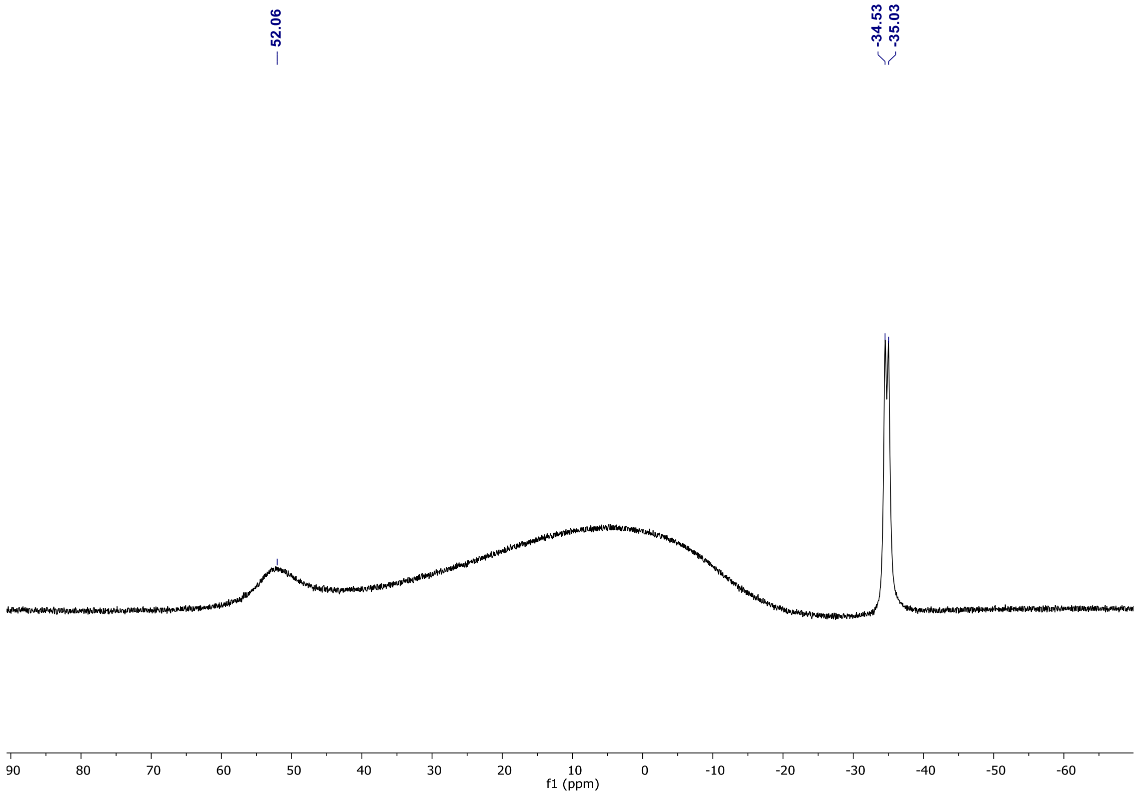

Figure S17. ^{11}B NMR spectrum of **4** in THF-d_8.

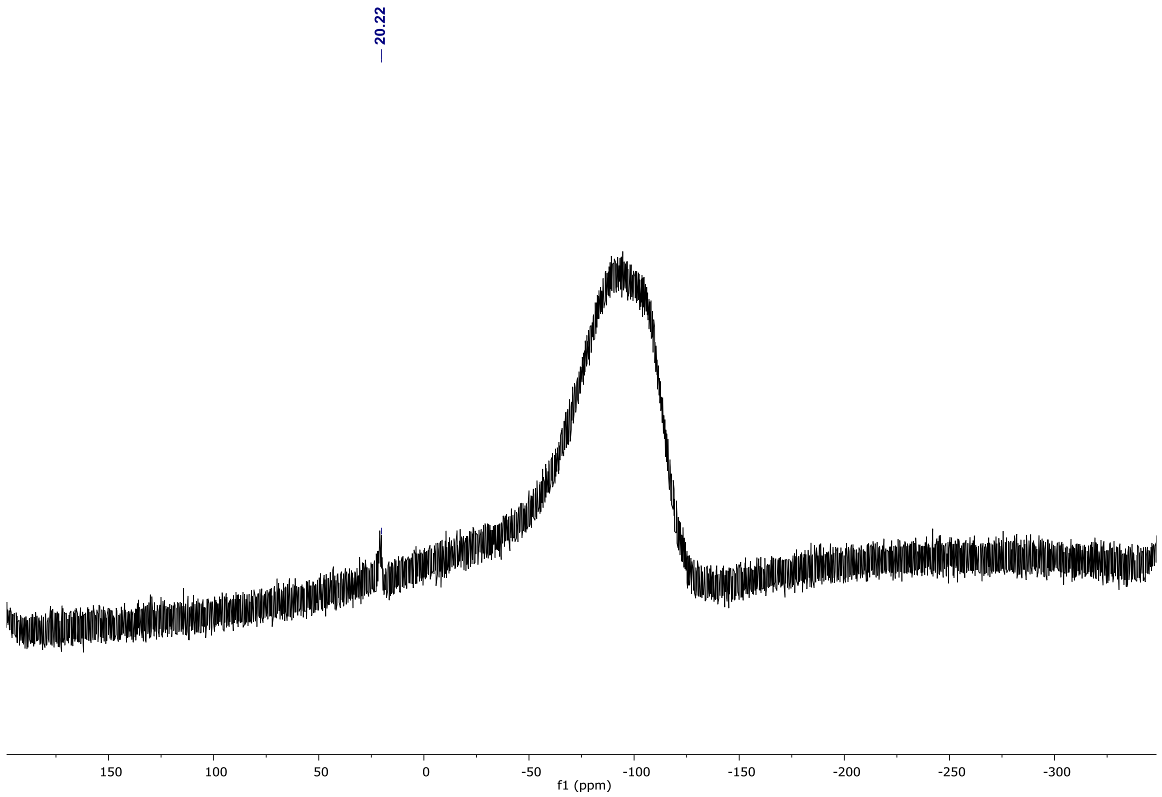

Figure S18. $^{29}Si\{^1H\}$ NMR spectrum of **4** in THF-d_8.

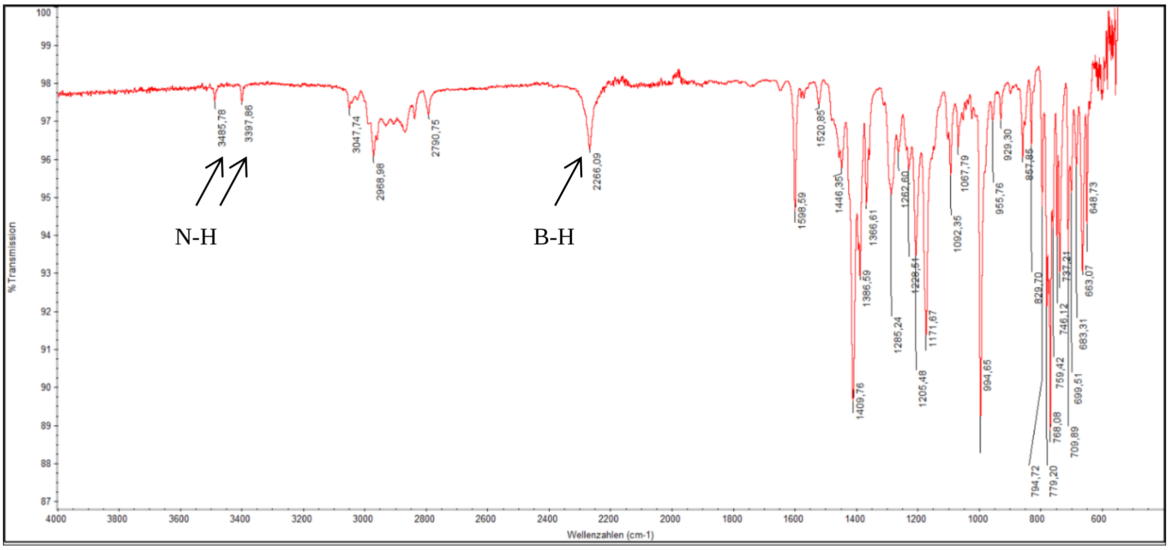

Figure S19. IR spectrum of **4.**

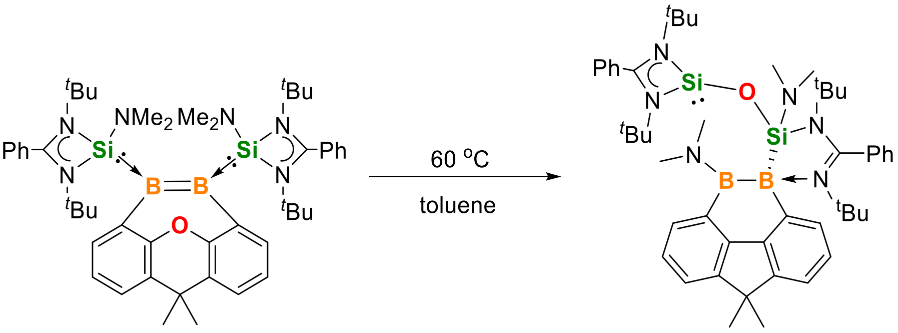

2 **5**

Synthesis of compound 5. The tolulene solution of **2** (418 mg, 0.5 mmol) in a 50 mL Schlenk flask was heated at 60 °C under stirring. The color of the mixture changed from red to yellow slowly. After stirring for 5 hours, all volatiles were removed under vacuum and the residue was washed with Et_2O (15 mL) to afford compound **5** as a white powder after dried under vacuum (292 mg, 69% isolated yield). Colorless block crystals suitable for X-ray single crystal diffraction analysis were obtained from a concentrated toluene solution at room temperature. **5** is soluble in toluene during the reaction. However, once it crystallized from toluene, its solubility is bad. We have tried various *d*-solvents to characterize its NMR. Its solubility is poor in benzene-d_6, THF-d_8, pyridine-d_5 and acetonitrile-d_3. It is not stable in DCM-d_2, $CDCl_3$ and *o*-$C_6D_4Cl_2$ due to the precence of silylene moiety. The measurement of $^{29}Si\{^1H\}$ NMR of **5** requires the high concentration because of the quadrupole effect from adjacent boron atoms. The yield of **5** is quite good, we did a *in situ* NMR scale reaction in THF-d_8 to characterize its $^{29}Si\{^1H\}$ NMR.

M.p.: 222.9 °C.

^{1}H NMR (500 MHz, THF-d_8) δ/ppm = 7.93 (d, J = 7.6 Hz, 1H, Ar-*H*), 7.75 – 7.71 (m, 2H, Ar-*H*), 7.51 (d, J = 7.4 Hz, 1H, Ar-*H*), 7.46 (m, , 2H, Ar-*H*), 7.40 – 7.36 (m, 2H, Ar-*H*), 7.30 (m, 2H, Ar-*H*), 7.27 – 7.22 (m, 3H, Ar-*H*), 7.04 (t, J = 7.5 Hz, 1H, Ar-*H*), 6.92 (t, J = 7.3 Hz, 1H, Ar-*Hk*), 6.87 (d, J = 7.2, 1H), 3.28 (s, 6H, $N(CH_3)_2$), 2.15 (s, 6H, $N(CH_3)_2$), 1.39 (s, 3H, $C(CH_3)_2$), 1.19 (d, J = 6.2 Hz, 18H, $C(CH_3)_3$), 1.10 (s, 9H, $C(CH_3)_3$), 0.97 (s, 3H, $C(CH_3)_2$), 0.55 (s, 9H, $C(CH_3)_3$).

^{13}C NMR (101 MHz, THF-d_8) δ 163.01(s, N*C*N), 152.29, 151.69, 137.07, 135.34, 135.12, 134.55, 132.32, 132.16, 130.96, 130.30, 129.89, 128.95, 128.44, 128.23, 128.13, 127.16, 126.90, 124.48, 124.17, 121.90 (s, Ar-*C*), 62.65 (s, $C(CH_3)_3$), 57.83(s, $C(CH_3)_3$), 53.16(s, $C(CH_3)_3$), 53.08 (s, $C(CH_3)_3$), 47.04 (s, $N(CH_3)_2$), 46.70 (s, $C(CH_3)_2$), 43.91(s, $N(CH_3)_2$), 39.20 (s, $N(CH_3)_2$), 34.34 (s, $C(CH_3)_3$), 33.26 (s, $C(CH_3)_3$), 32.94 (s, $C(CH_3)_3$), 32.29 (s, $C(CH_3)_3$), 31.14 (s, $C(CH_3)_2$), 29.15 (s, $C(CH_3)_2$).

^{11}B{^{1}H} NMR (160 MHz, THF-d_8) δ/ppm = 55.00 (*B*NMe$_2$), -19.53 (*B*Si).

^{29}Si{^{1}H} NMR (99 MHz, THF-d_8) δ/ppm = 21.46 (*Si*-silylene), -22.32 (*Si*B).

HR-MS (ESI): (m/z) calcd for $[M+H]^+$ ($C_{49}H_{71}B_2N_6O_1Si_2^+$): 837.5409; Found: 837.5435

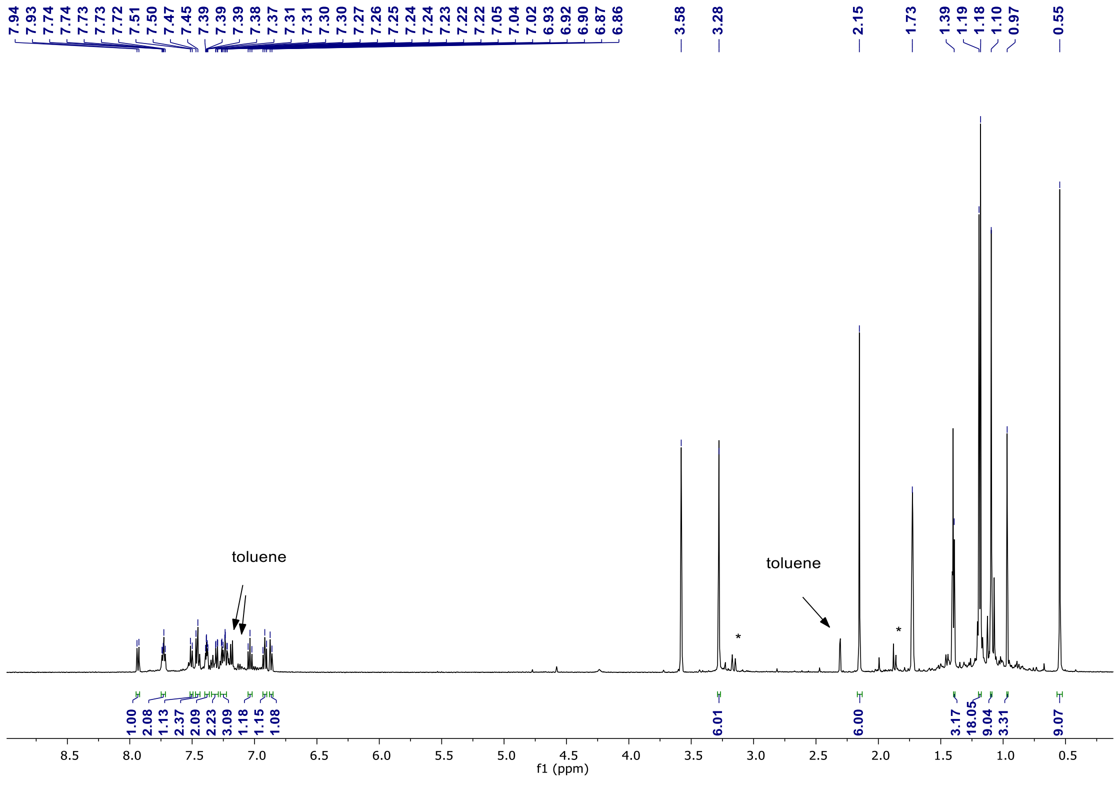

Figure S20. ^{1}H NMR spectrum of **5** in THF-d_8. *Unidentified impurities.

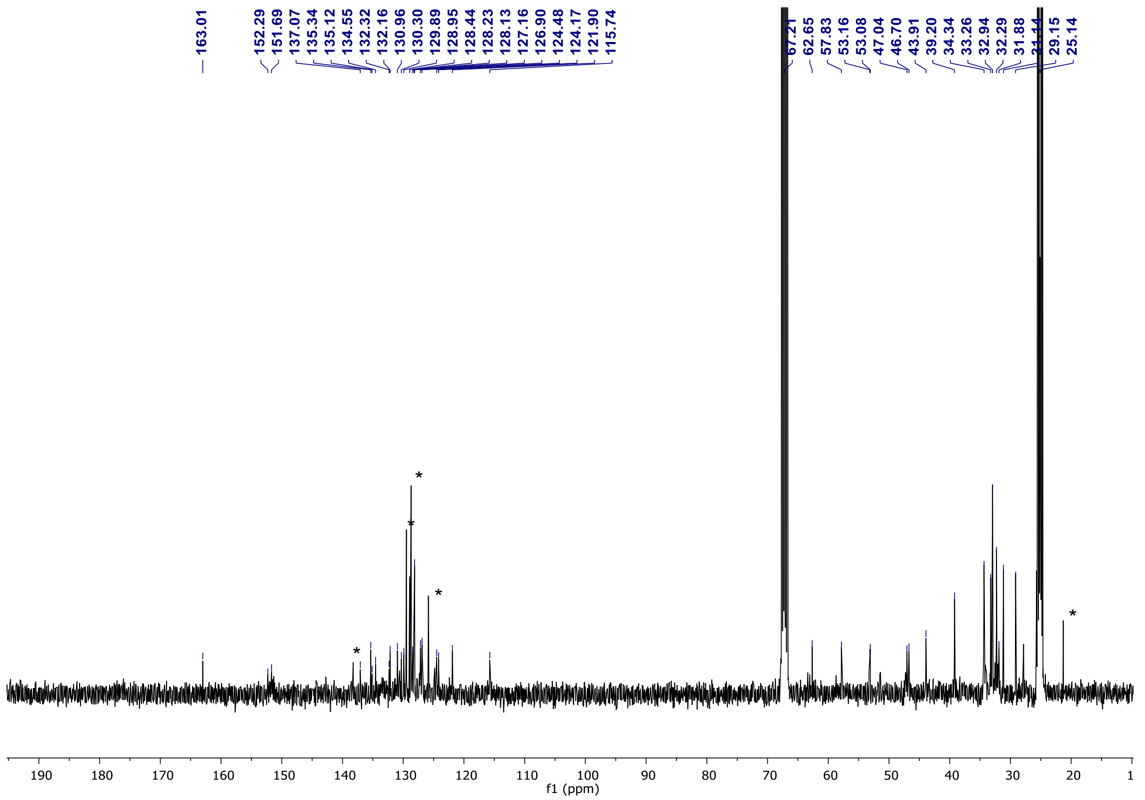

Figure S21. $^{13}C\{^{1}H\}$ NMR spectrum of **5** in THF-d_8. * is solvent (Toluene).

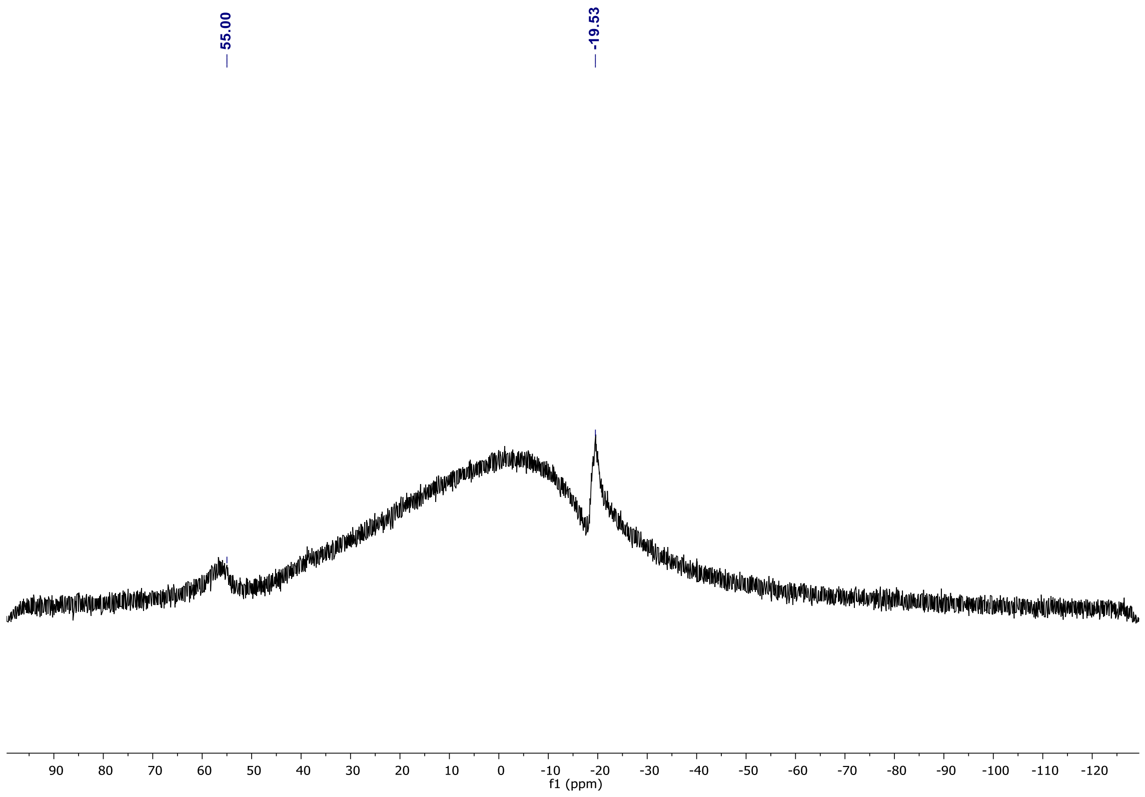

Figure S22. $^{11}B\{^{1}H\}$ NMR spectrum of **5** in THF-d_8.

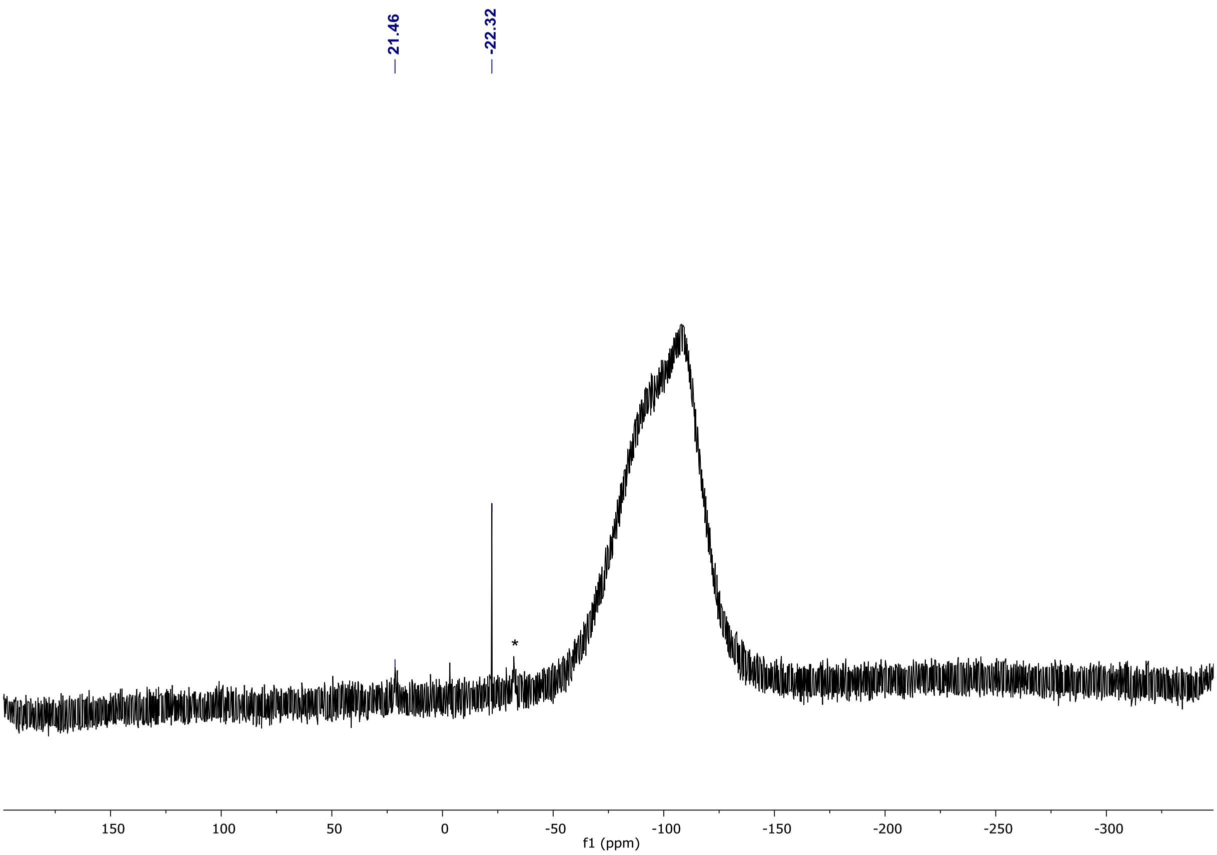

Figure S23. *In situ* $^{29}Si\{^1H\}$ NMR spectrum of **5** in THF-d_8. * is the unidentified signal.

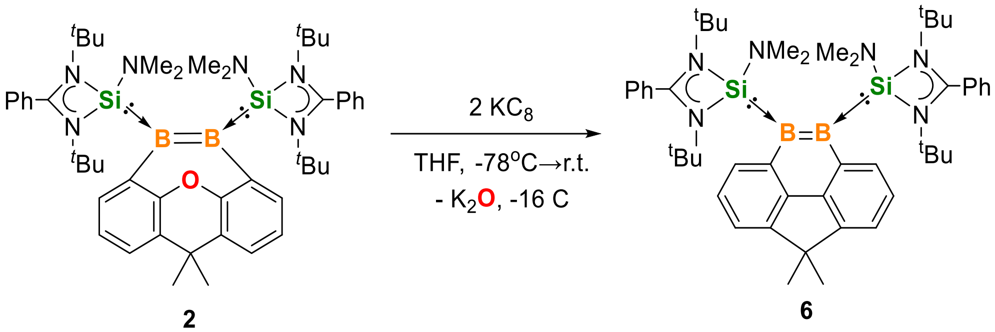

Synthesis of compound 6. To the mixture of **2** (1.67 g, 2 mmol) and KC_8 (540 mg, 4 mmol) in a 100 mL Schlenk flask was added 60 mL THF at -78 °C under stirring. The mixture was allowed to warm up to room temperature and stirred for 2 h. The dark red mixture was filtered and the residue was washed with THF (10 mL x 3). The all volatiles were removed and recrystallization from THF at 4 °C to afford compound **6** as a red crystalline solid (492 mg, 30% isolated yield). Red block crystals suitable for X-ray diffraction analysis were obtained from a concentrated DME solution at -20 °C.

M.p.: 191.3 °C.

^{1}H NMR (500 MHz, THF-d_8) δ/ppm = 8.14 (d, J = 7.8 Hz, 2H, Ar-H), 8.02 (d, J = 7.7 Hz, 2H, Ar-H), 7.68 (d, J = 7.6 Hz, 2H, Ar-H), 7.63 – 7.41 (m, 6H, Ar-H), 7.22 – 7.18 (m, 2H), 7.02 (d, J = 6.6 Hz, 2H), 3.18 (s, 12H, $N(CH_3)_2$), 1.54 (s, 6H, $C(CH_3)_2$), 1.07 (s, 36H, $C(CH_3)_3$).

^{13}C{^{1}H} NMR (126 MHz, THF-d_8) δ/ppm = 174.02 (s, N*C*N), 152.50, 147.09, 141.33, 133.33, 131.20, 129.79, 129.27, 129.10, 128.78, 128.07, 120.71, 110.97 (s, Ar-*C*), 54.30 (s, $NC(CH_3)_3$), 47.01 (s, $C(CH_3)_2$, 40.47 (s, $N(CH_3)_2$), 31.40 (s, $C(CH_3)_2$, 28.15 (s, $C(CH_3)_2$).

^{11}B{^{1}H} NMR (160 MHz, THF-d_8) δ/ppm = 29.19.

^{29}Si{^{1}H} NMR (99 MHz, THF-d_8) δ/ppm = 17.67.

HR-MS (ESI): (m/z) calcd for [M+H]$^{+}$ ($C_{49}H_{71}B_2N_6Si_2^{+}$) 821.5459; Found: 821.5457.

UV-Vis (THF), λ_{max}: 396, 478 nm.

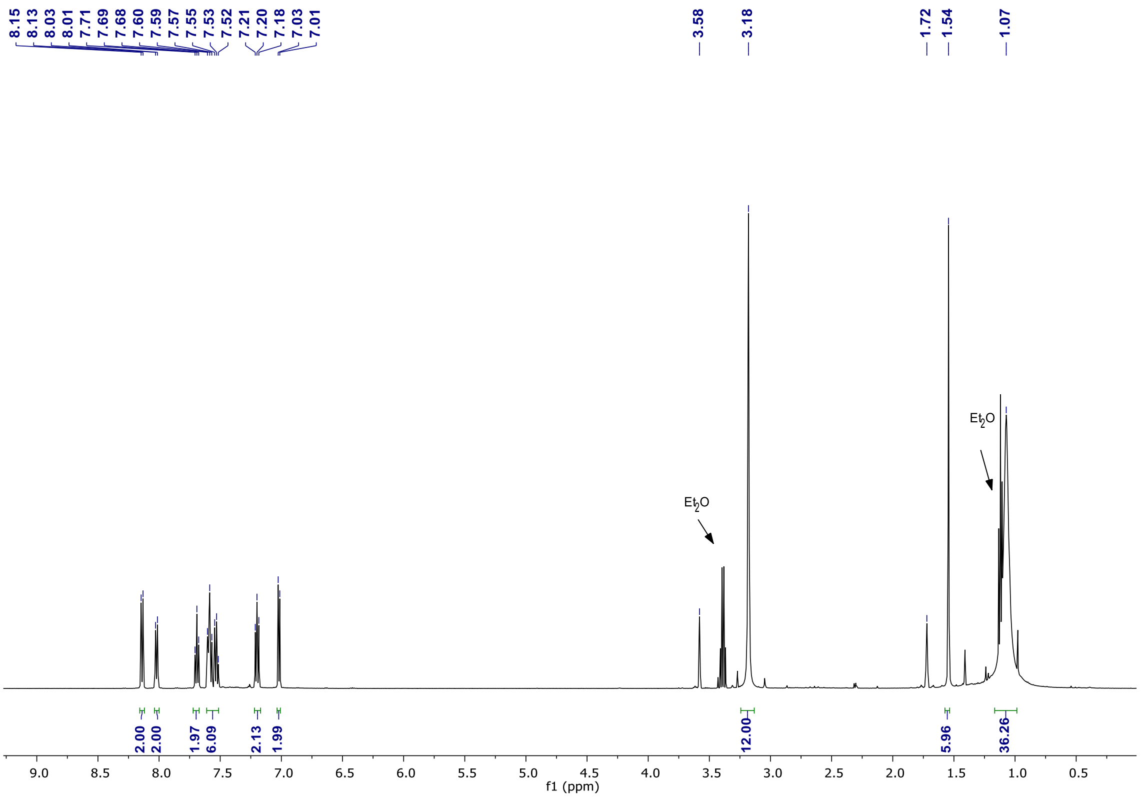

Figure S24. ^{1}H NMR spectrum of **6** in in THF-d_8.

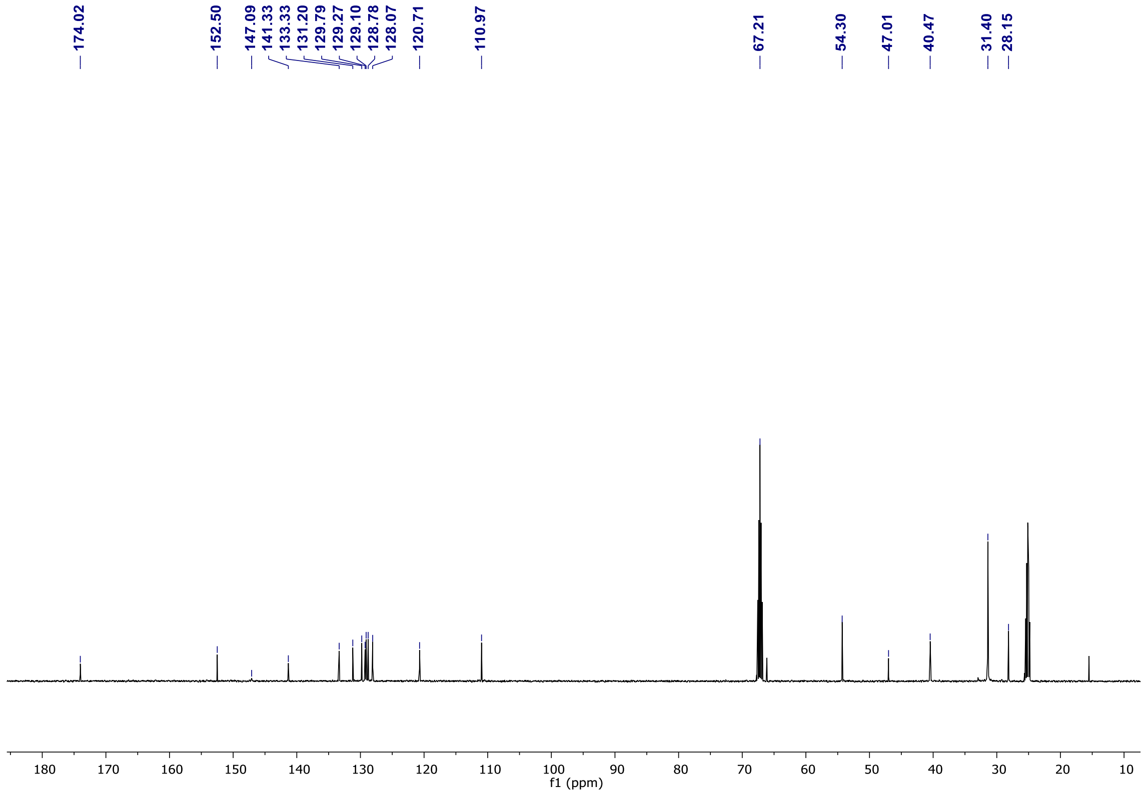

Figure S25. $^{13}C\{^{1}H\}$ NMR spectrum of **6** in in THF-*d*$_8$.

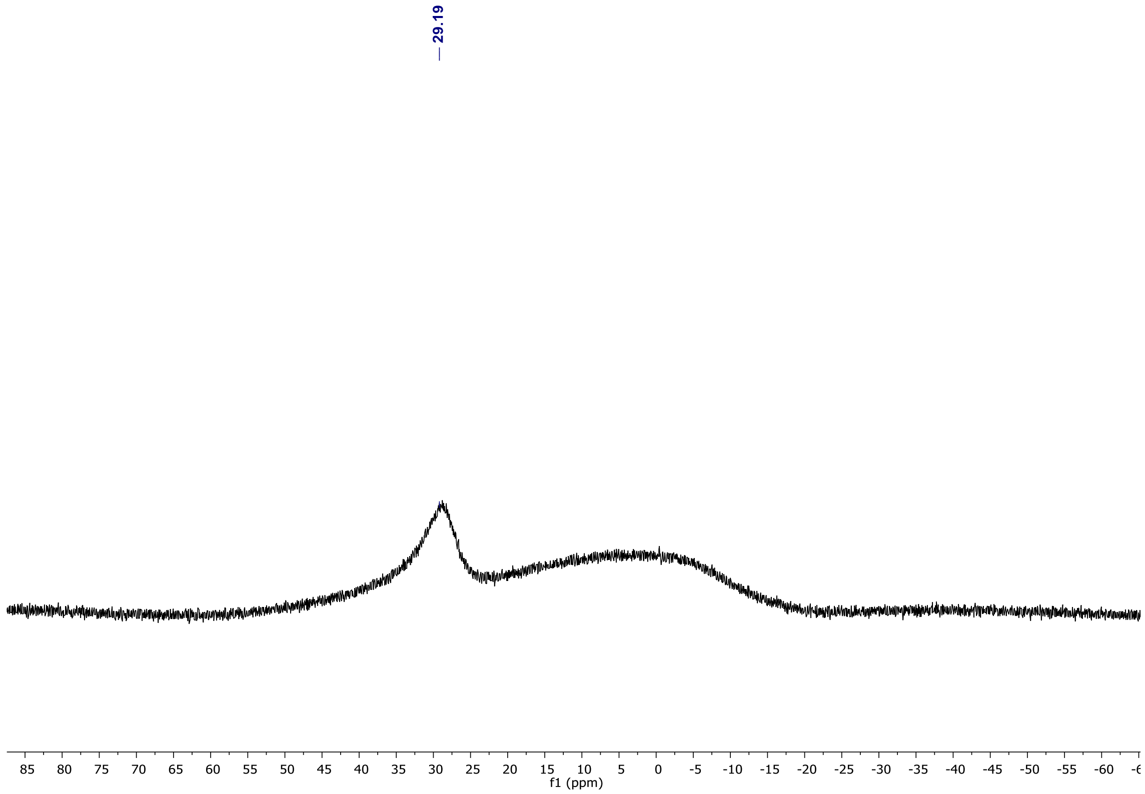

Figure S26. $^{11}B\{^{1}H\}$ NMR spectrum of **6** in in THF-*d*$_8$.

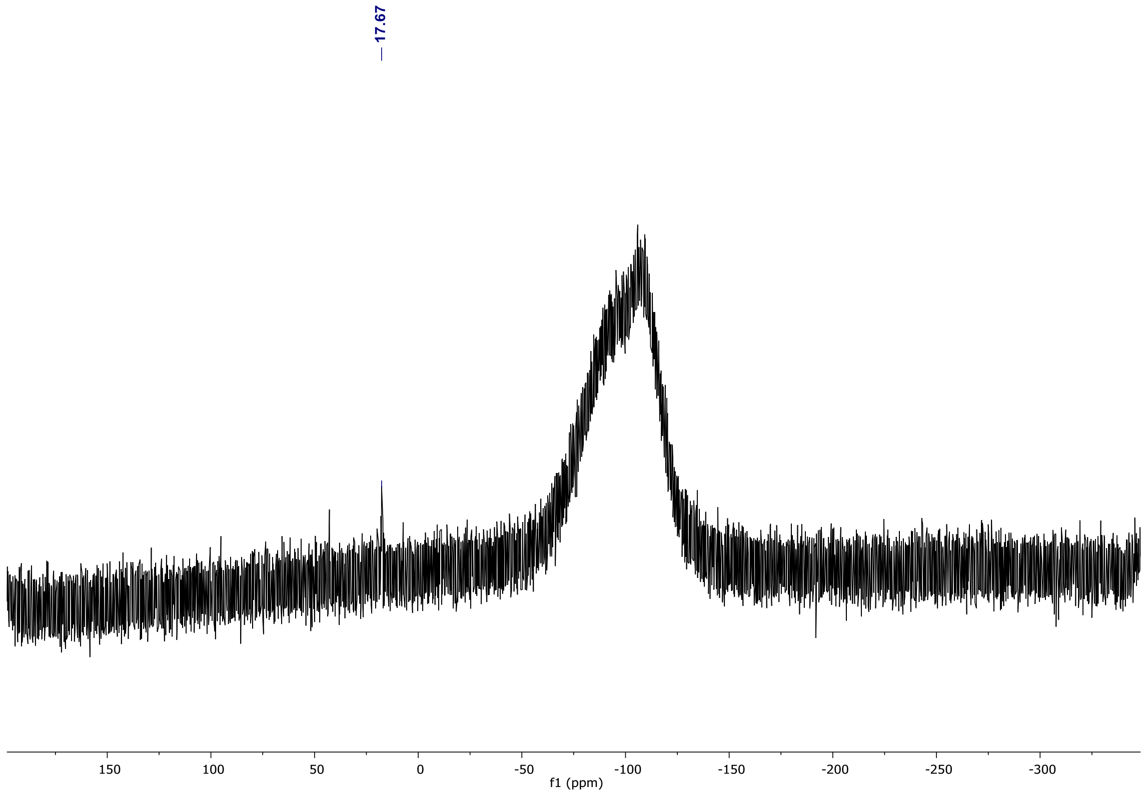

Figure S27. $^{29}Si\{^{1}H\}$ NMR spectrum of **6** in THF-d_8.

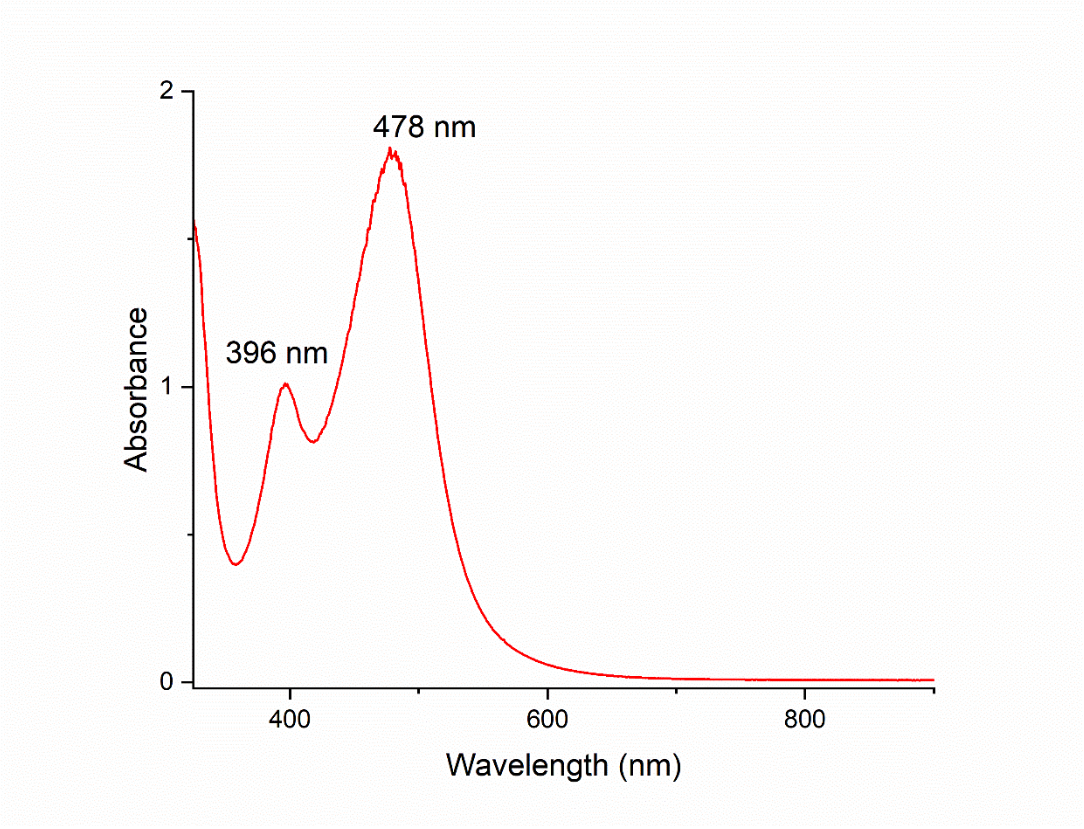

Figure S28. UV/Vis spectrum of compound **6** (RT, THF).

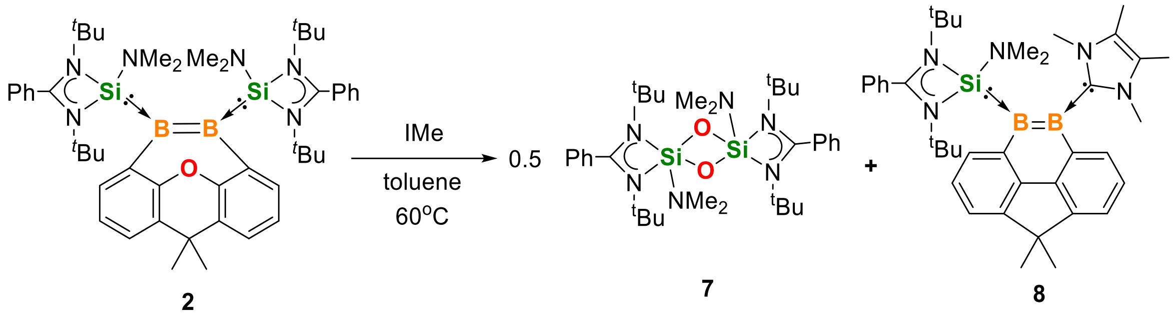

Synthesis of compound 7 and 8. The toluene solution of **2** (836 mg, 1 mmol) and IMe carbene (124 mg, 1 mmol) in a 100 mL Schlenk flask was heated at 60 °C under stirring. After stirring for 12 hours, the reaction mixture was filtered to give a red solution. Compound **7** and **8** were isolated as colorless crystals and red crystals from toluene solution by fractional crystallization in 74% (236 mg) and 60% (385 mg) yields, respectively.

Compound 7:

M.p.: 307.1 °C.

^{1}H NMR (500 MHz, Benzene-d_6) δ/ppm = 7.46 – 7.43 (m, 1H, Ar-*H*), 7.39 (d, *J* = 7.5 Hz, 1H, Ar-*H*), 7.07 – 6.94 (m, 8H, Ar-*H*), 3.18 (s, 6H, N(C*H*$_3$)$_2$), 3.15 (s, 6H, N(C*H*$_3$)$_2$), 1.39 (s, 18H, C(C*H*$_3$)$_3$), 1.36 (s, 18H, C(C*H*$_3$)$_3$).

^{13}C{^{1}H} NMR (126 MHz, Benzene-d_6) δ/ppm = 170.99 (s, N*C*N), 170.00 (s, N*C*N), 135.73, 135.70, 130.32, 130.28, 129.23, 129.18, 128.49, 128.36, 128.16, 127.97, 127.57, 127.44, (s, Ar-*C*) 53.33 (s, N*C*(CH$_3$)$_3$), 53.30 (s, N*C*(CH$_3$)$_3$), 40.74 (s, N(*C*H$_3$)$_2$), 40.56 (s, N(*C*H$_3$)$_2$), 31.82 (s, C(*C*H$_3$)$_3$), 31.68 (s, C(*C*H$_3$)$_3$).

^{29}Si{^{1}H} NMR (99 MHz, Benzene-d_6) δ/ppm = -99.46 (s), -100.17 (s).

HR-MS (ESI): (m/z) calcd for $[M\text{-}NMe_2]^+$ ($C_{32}H_{52}N_5O_2Si_2^+$) 594.3654; Found: 594.3650.

Compoound 8:

M.p.: 278.3 °C.

^{1}H NMR (500 MHz, THF-d_8) δ/ppm = 7.97 (d, J = 7.8 Hz, 1H, Ar-H), 7.85 (d, J = 7.6 Hz, 1H, Ar-H), 7.65 (t, J = 7.5 Hz, 1H, Ar-H), 7.56 (t, J = 7.5 Hz, 2H, Ar-H), 7.52 (d, J = 8.2 Hz, 1H, Ar-H), 7.15 (m, 1H, Ar-H), 6.96 – 6.91 (m, 3H, Ar-H), 6.77 (d, J = 7.9 Hz, 1H, Ar-H), 3.66 (s, 6H, N(CH_3)$_2$), 2.62 (s, 6H, NCH_3), 2.24 (s, 6H, CCH_3), 1.55 (s, 6H, C(CH_3)$_2$), 1.10 (s, 18H, C(CH_3)$_3$).

^{13}C{^{1}H} NMR (126 MHz, THF-d_8) δ/ppm = 173.59 (s, NCN), 152.49 (s, Ar-C), 142.07 (s, Ar-C), 140.45 (s, Ar-C), 133.19 (s, Ar-C), 131.17 (s, Ar-C), 130.20 (s, Ar-C), 130.09 (s, Ar-C), 129.07 (s, Ar-C), 128.82 (s, Ar-C), 128.17(s, Ar-C), 125.14 (s, Ar-C), 123.90 (s, IMe-C4,5), 121.42 (s, Ar-C), 120.82 (s, Ar-C), 110.87 (s, Ar-C), 54.09 (s, NC(CH$_3$)$_3$), 47.34 (s, C(CH$_3$)$_2$), 38.51(s, IMe-CCH$_3$), 33.90 (s, N(CH$_3$)$_2$), 31.28 (s, C(CH$_3$)$_3$), 27.98 (s, C(CH$_3$)$_2$), 8.47(s, IMe-NCH$_3$).

^{11}B{^{1}H} NMR (160 MHz, THF-d_8) δ/ppm = 33.47 (s), 11.61 (s).

^{29}Si{^{1}H} NMR (99 MHz, THF-d_8) δ/ppm = 21.90 (s).

HR-MS (ESI): (m/z) calcd for $[M+H]^+$ ($C_{39}H_{54}B_2N_5Si_1^+$) 642.4329; Found: 642.4324.

UV-Vis (THF), λ_{max}: 408, 464 nm.

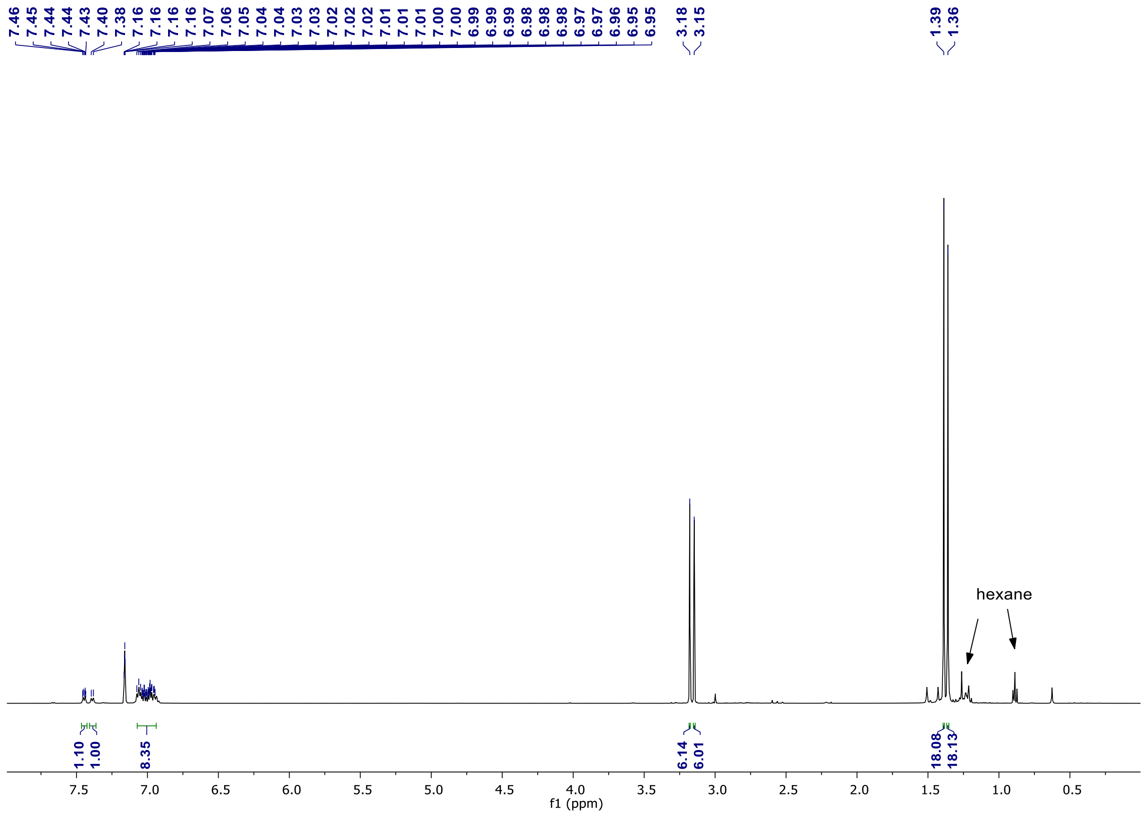

Figure S29. ^{1}H NMR spectrum of **7** in Benzene-d_{6}.

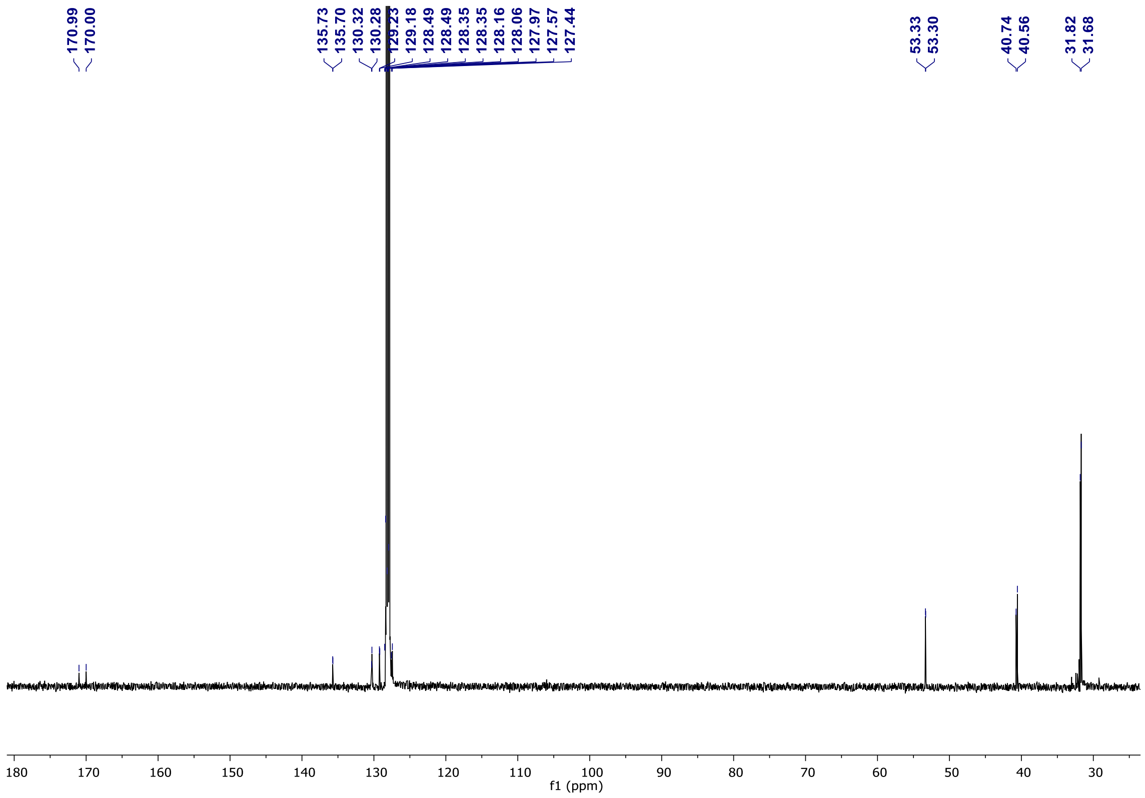

Figure S30. $^{13}C\{^{1}H\}$ NMR spectrum of **7** in Benzene-d_{6}.

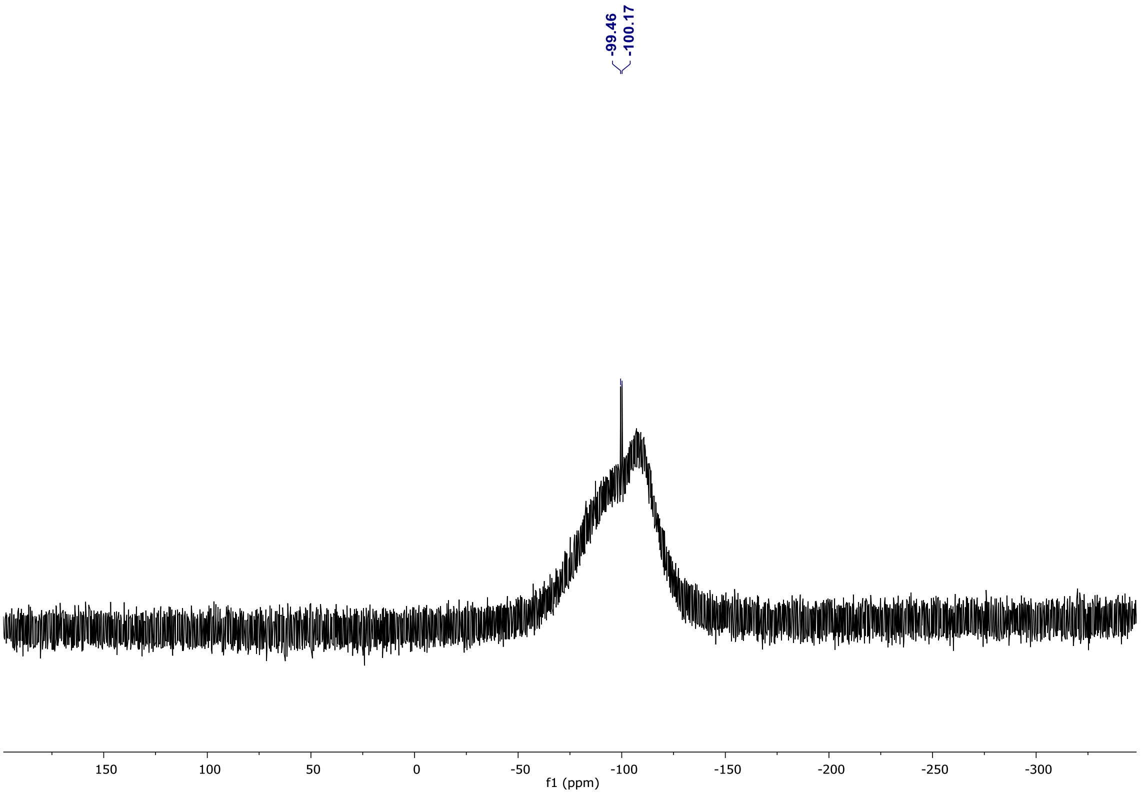

Figure S31. $^{29}Si\{^{1}H\}$ NMR spectrum of **7** in Benzene-d_6.

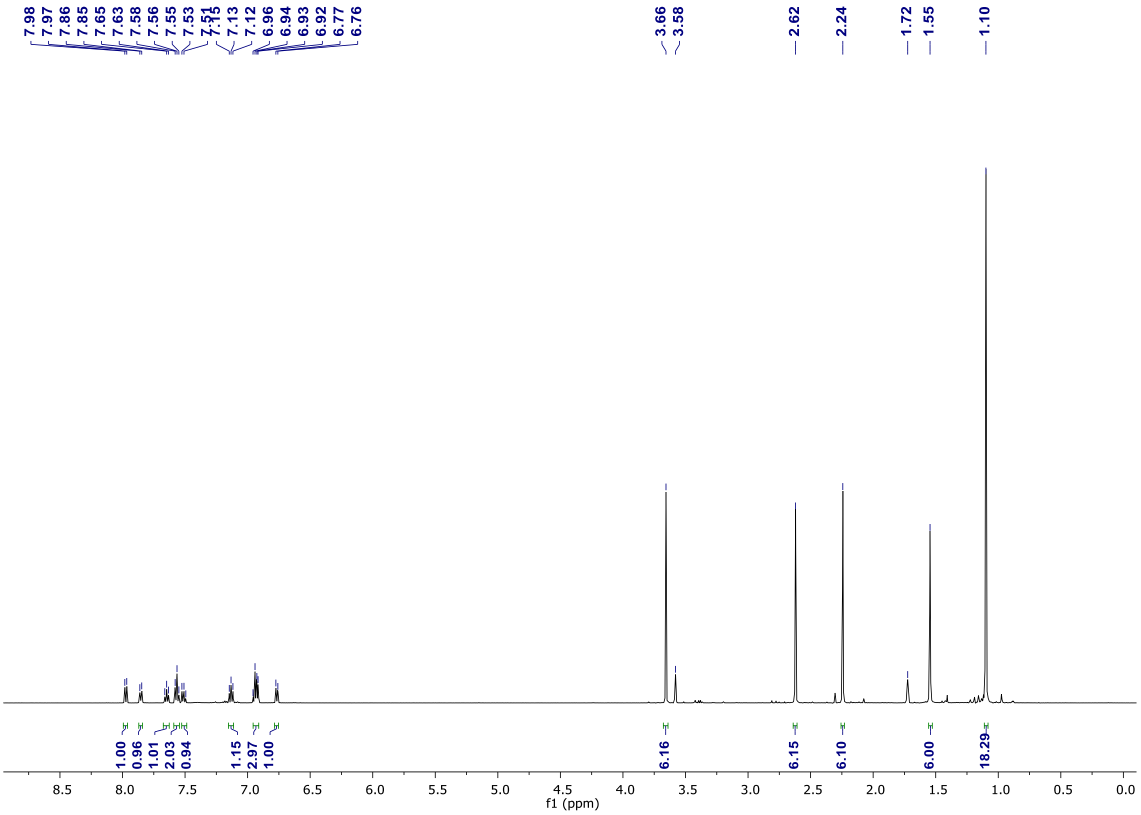

Figure S32. ^{1}H NMR spectrum of **8** in THF-d_8.

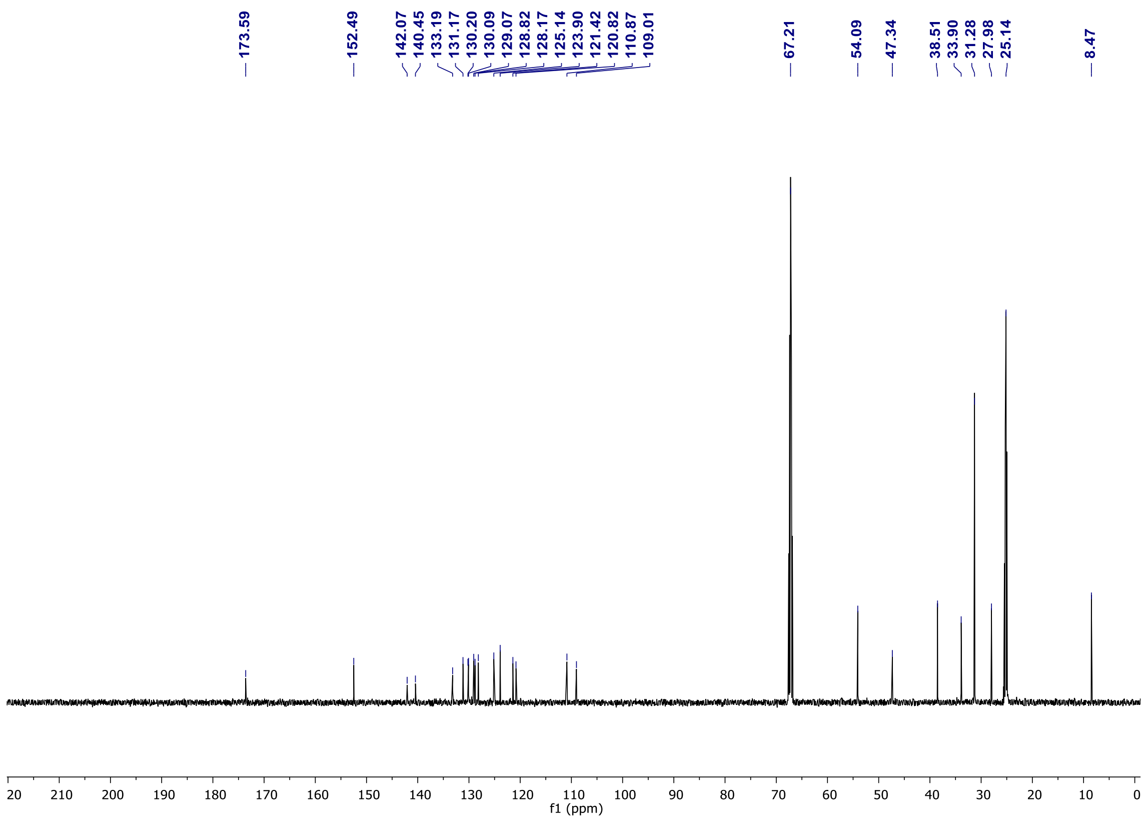

Figure S33. $^{13}C\{^{1}H\}$ NMR spectrum of **8** in THF-d_8.

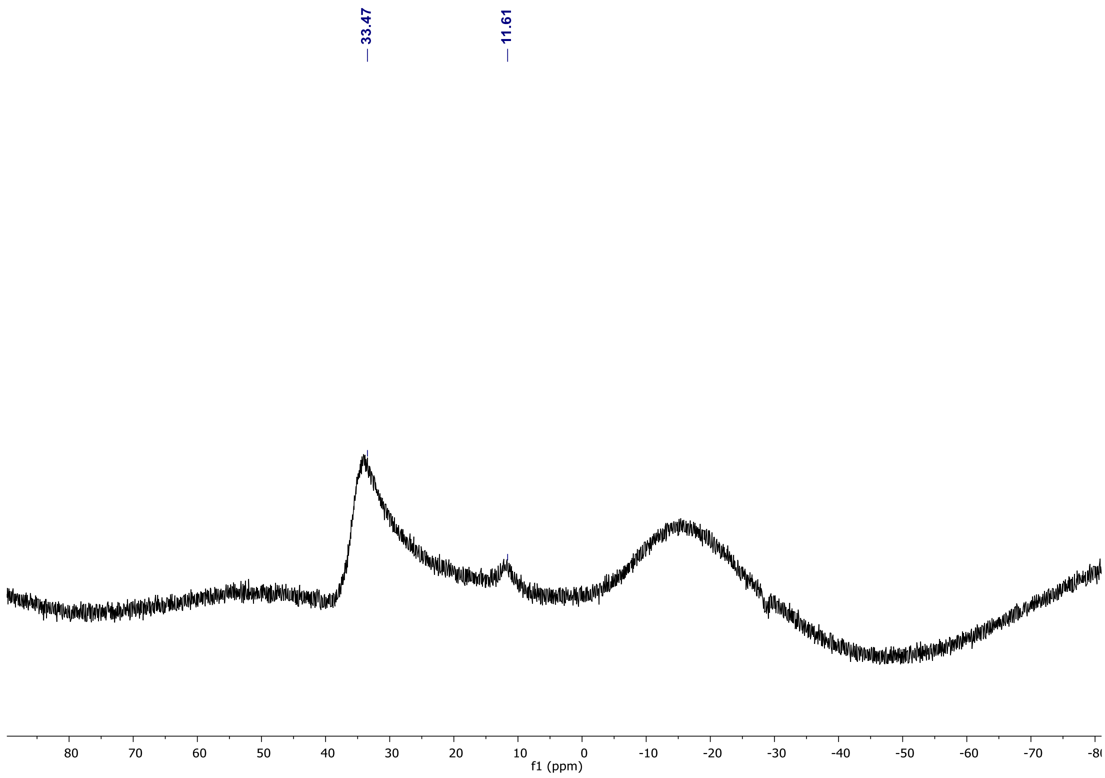

Figure S34. $^{11}B\{^{1}H\}$ NMR spectrum of **8** in THF-d_8.

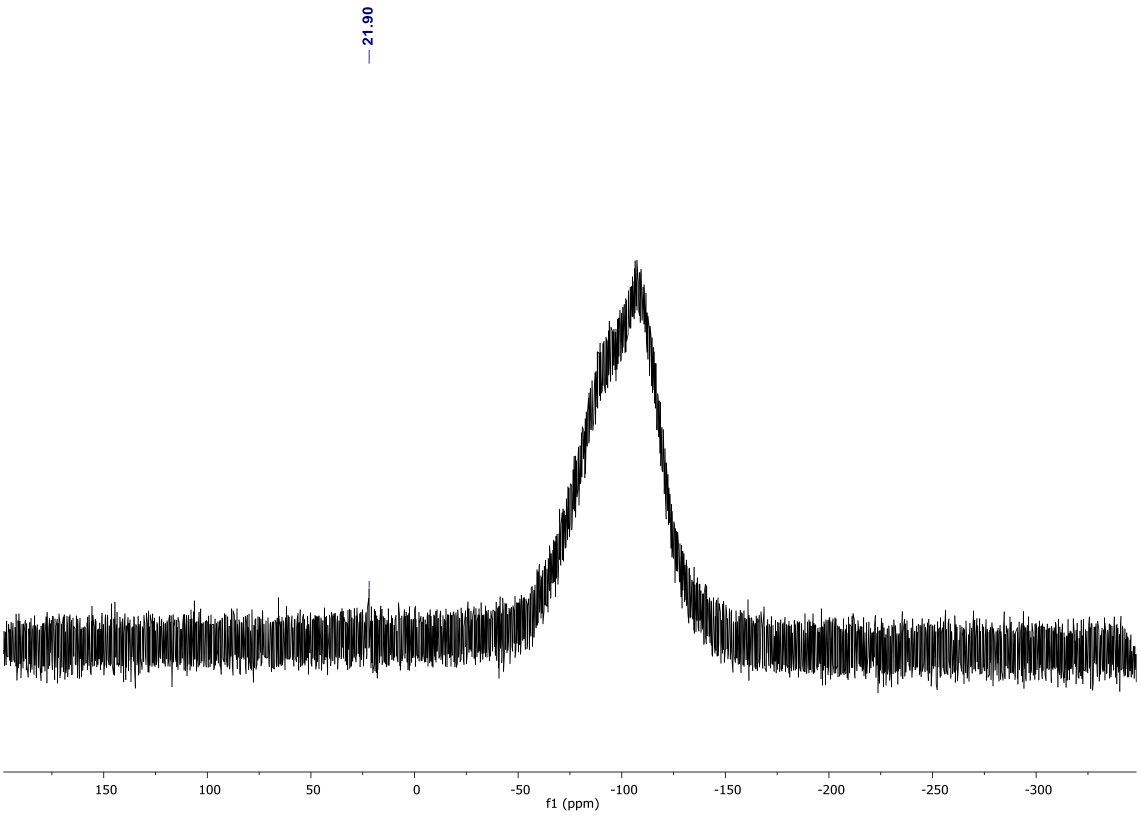

Figure S35. $^{29}Si\{^{1}H\}$ NMR spectrum of **8** in THF-d_8.

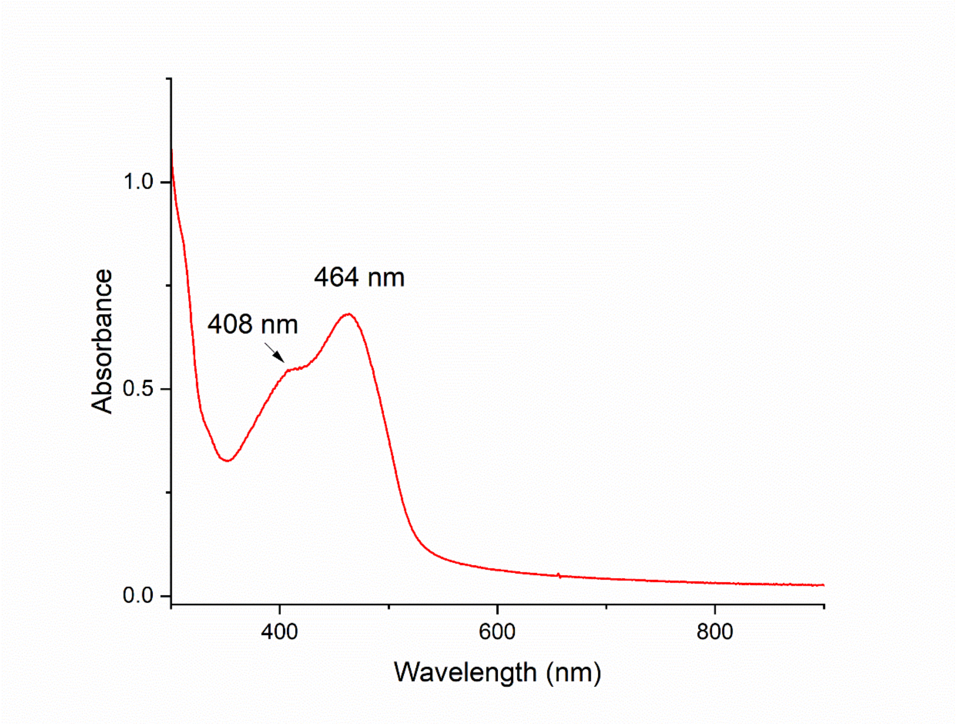

Figure S36. UV/Vis spectrum of compound **8** (RT, THF).

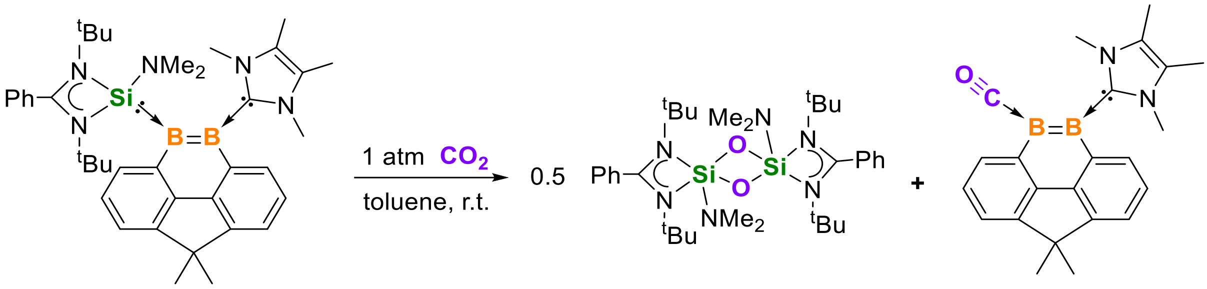

Synthesis of compound 9. After three freeze-pumpthaw cycles, the toluene solution of compound **8** (642 mg, 1 mmol) in a 100 mL Schlenk flask was subjected to 1 atm CO_2 at room temperature under stirring. Then the mixture was stirred for 10 min. All volatiles were removed to give a yellow solid, which was washed with 15 mL Et_2O to remove the minor product **10** and other impurity (see below). The residuals was dry under vacuum, after which 8 mL toluene was added. After filteration and concentration, yellow crystals of compound **9** suitable for X-ray diffraction analysis were obtained from the toluene solution. This reaction is complicated and three products as well as some impurity were formed. In addition, the solubility of compounds **7** and **9** are very similar, they always crystallize together. It is hard to determinate the yield of products.

M.p.: 163.2 °C (dec.):

^{1}H NMR (500 MHz, THF-d_8) δ/ppm = 7.63 (d, J = 7.3 Hz, 1H, Ar-H), 7.23 – 7.11 (m, 4H, Ar-H), 7.01 (d, J = 7.5 Hz, 1H, Ar-H), 3.65 (s, 6H, IMe-CH_3), 2.31 (s, 6H, IMe-CH_3), 1.56 (s, 6H, C(CH_3)$_2$).

^{13}C{^{1}H} NMR (126 MHz, THF-d_8) δ/ppm = 153.38, 152.67, 146.32, 141.72, 131.95 (s, Ar-C), 126.35 (s, IMe-C4,5), 125.61, 124.91, 124.24, 119.49, 114.64 (s, Ar-C), 48.14 (s, C(CH$_3$)$_2$), 33.43 (s, IMe-CCH$_3$), 27.47 (s, C(CH$_3$)$_2$), 8.26 (s, IMe-NCH$_3$).

^{11}B{^{1}H} NMR (160 MHz, THF-d_8) δ/ppm = 63.48 (s), -11.75 (s).

HR-MS (APCI): (m/z) calcd for $[M]^+$ ($C_{23}H_{24}B_2N_2O_1$) 366.2069; Found: 366.2061.

IR (cm^{-1}): 1935.20 (s, CO)

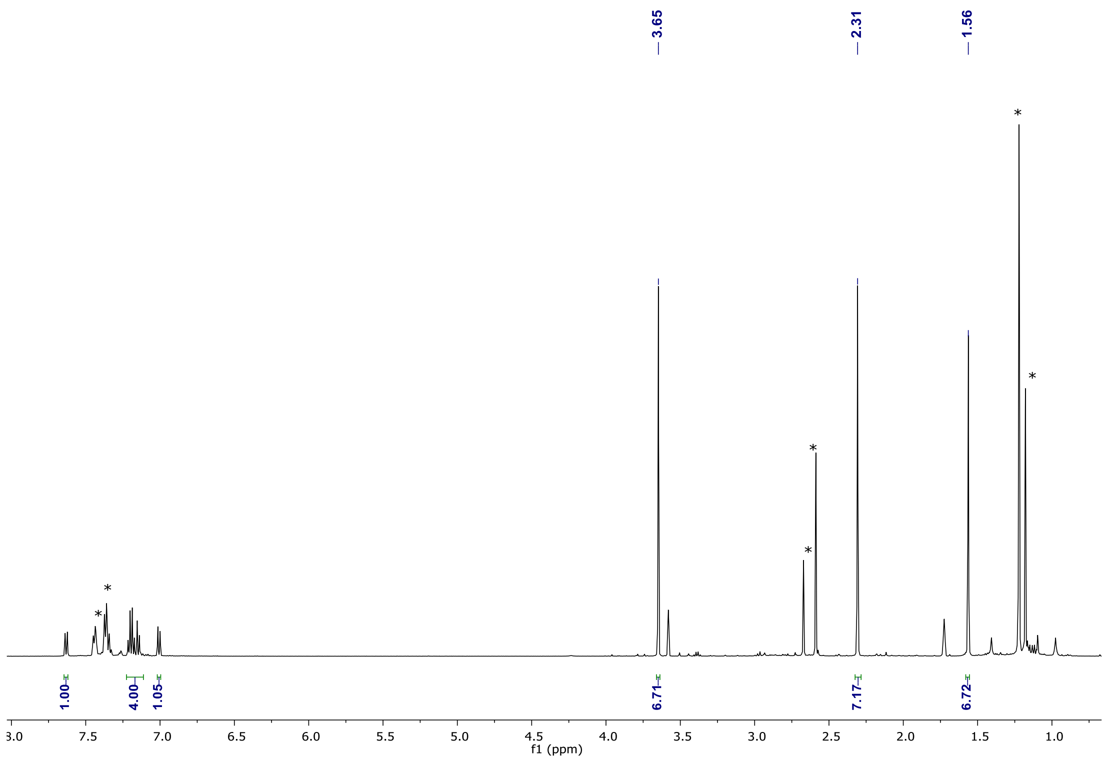

Figure S37. ^{1}H NMR spectrum of **9** in THF-d_8. *Compound **7**.

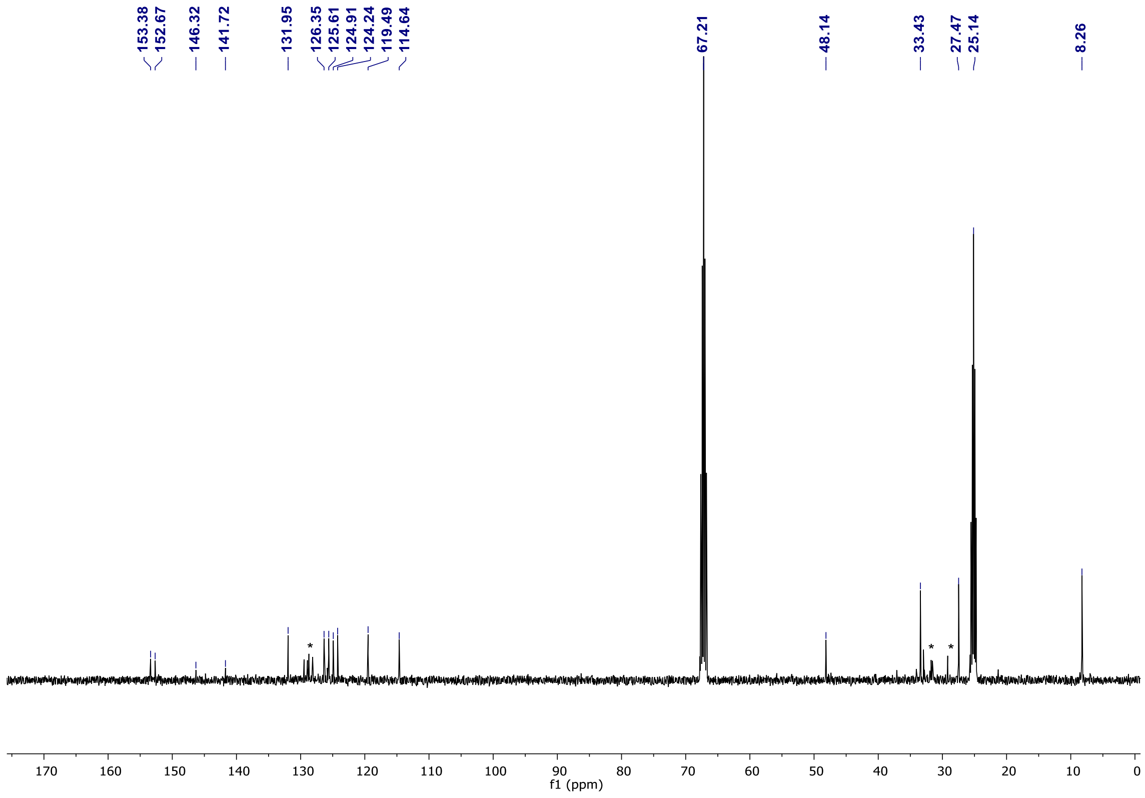

Figure S38. $^{13}C\{^{1}H\}$ NMR spectrum of **9** in THF-d_8. *Compound **10**.

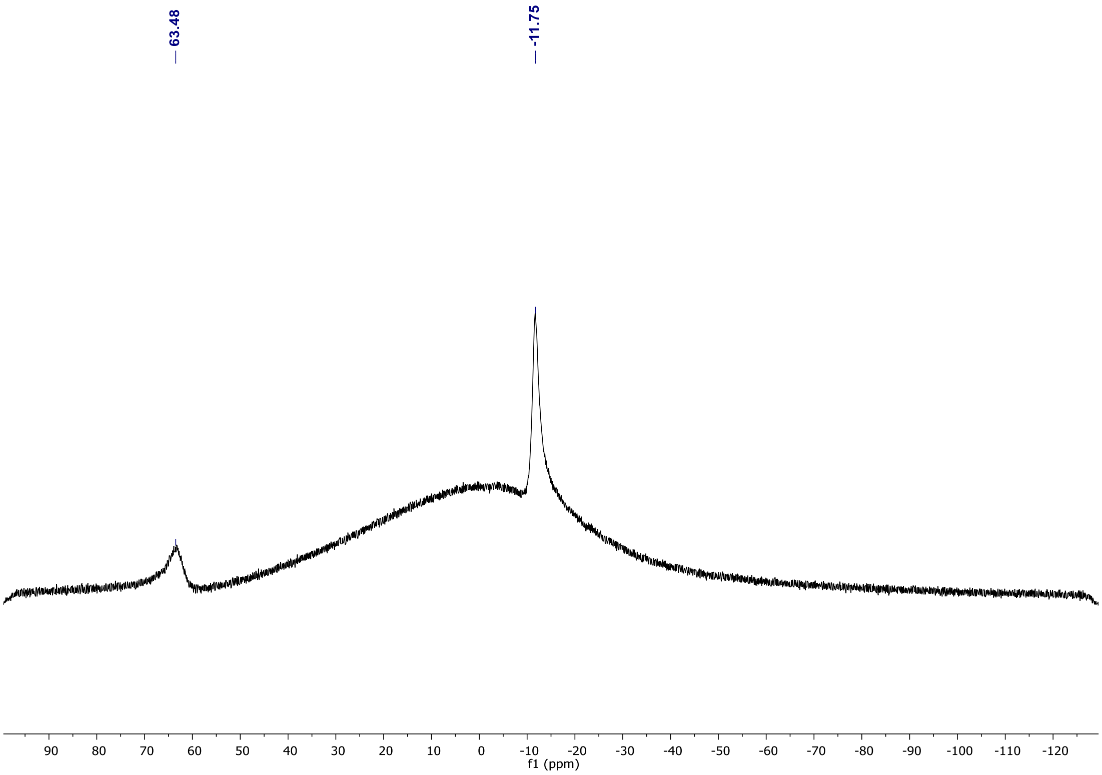

Figure S39. $^{11}B\{^1H\}$ NMR spectrum of **9** in THF-d_8.

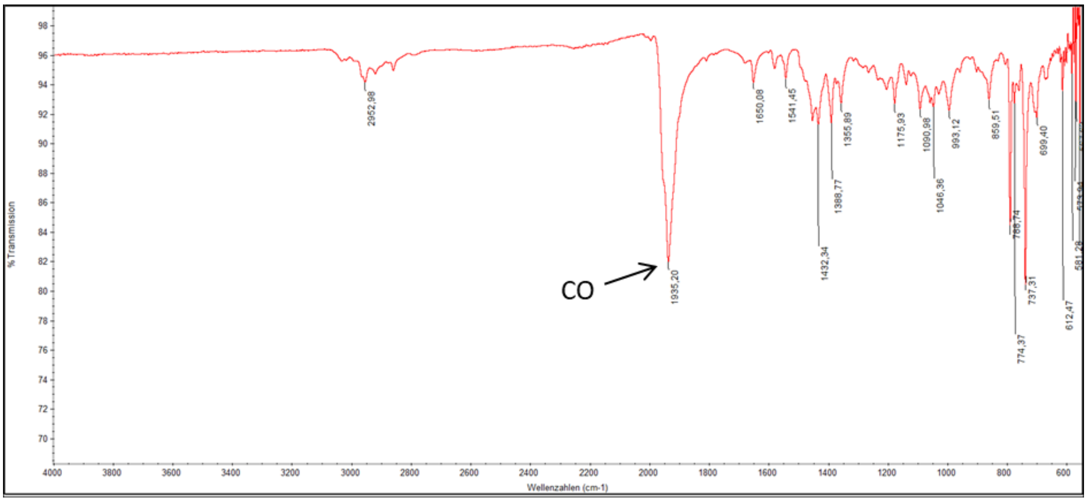

Figure S40. IR spectrum of **9**.

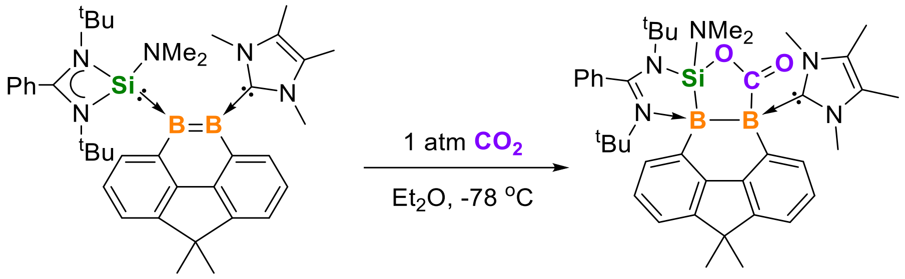

8 **10**

Synthesis of compound 10. After three freeze-pumpthaw cycles, the Et_2O suspension of compound **8** (642 mg, 1 mmol) in a 100 mL Schlenk flask was subjected to 1 atm CO_2 at -78 °C under stirring. The mixture was allowed to warm up to -20 °C and stirred for 30 min. The color of the mixture changed from red to yellow. The yellow suspension was filtered and concentrated to afford major product **10** as yellow single crystals (568 mg, 83% isolated yield).

M.p.: 203.3 °C (dec.).

^{1}H NMR (400 MHz, THF-d_8) δ/ppm = 7.70 (d, J = 8.2 Hz, 1H, Ar-H), 7.58 – 7.54 (m, 1H, Ar-H), 7.49 (t, J = 6.9 Hz, 1H, Ar-H), 7.42 (t, J = 6.9 Hz, 2H, Ar-H), 7.32 (d, J = 7.4 Hz, 1H, Ar-H), 7.15 (d, J = 6.8 Hz, 1H, Ar-H), 7.02 (d, J = 7.1 Hz, 1H, Ar-H), 6.91 – 6.84 (m, 2H, Ar-H), 6.76 (d, J = 7.1 Hz, 1H, Ar-H), 3.65 (s, 3H, IMe-NCH_3), 2.68 (s, 3H, IMe-NCH_3), 2.16 (s, 3H, IMe-CCH_3), 1.97 (s, 3H, IMe-CCH_3), 1.92 (s, 6H, N(CH_3)$_2$), 1.41 (s, 6H, C(CH_3)$_2$), 1.06 (s, 9H, NC(CH_3)$_3$), 0.96 (s, 9H, NC(CH_3)$_3$).

$^{13}C\{^{1}H\}$ NMR (101 MHz, THF-d_8) δ/ppm = 175.95 (s, NCN), 151.37, 151.19, 145.98, 140.55, 137.02, 135.73, 133.45, 131.60, 130.29, 129.21, 127.28, 126.99, 125.48, 125.13 (s,Ar-C), 123.99 (s, IMe-CCH$_3$), 123.88 (IMe--CCH$_3$), 118.44, 113.72 (s,Ar-C), 61.75 (s, C(CH$_3$)$_3$), 57.62 (s, C(CH$_3$)$_3$), 47.35 (s, C(CH$_3$)$_2$), 37.11 (s, C(CH$_3$)$_3$), 35.83 (s, IMe-NCH$_3$), 34.08 (s, C(CH$_3$)$_3$), 33.36 (s, IMe-NCH$_3$), 32.81(s, IMe-CCH$_3$), 28.84 (s, C(CH$_3$)$_2$), 27.49 (s, C(CH$_3$)$_2$), 8.60 (s, IMe-CCH$_3$), 8.34 (s, N(CH$_3$)$_2$).

$^{11}B\{^{1}H\}$ NMR (128 MHz, THF-d_8) δ/ppm = -11.08 (s), -16.89 (s).

$^{29}Si\{^{1}H\}$ NMR (79 MHz, THF-d_8) δ/ppm = 13.85 (s).

HR-MS (ESI): (m/z) calcd for $[M+H]^+$($C_{40}H_{54}B_2N_5O_2Si_1^+$): 686.4227; Found: 686.4246.

IR (cm^{-1}): 1666.16 (s, OC=O).

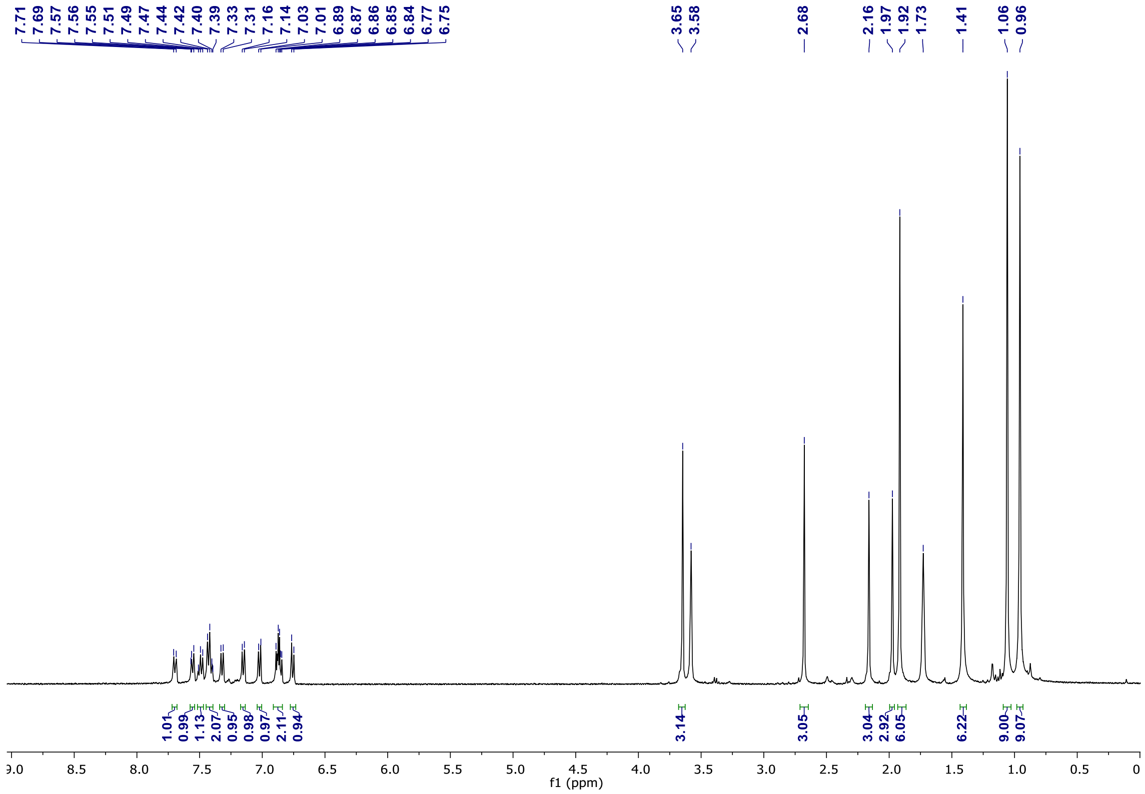

Figure S41. ^{1}H NMR spectrum of **10** in THF-d_8.

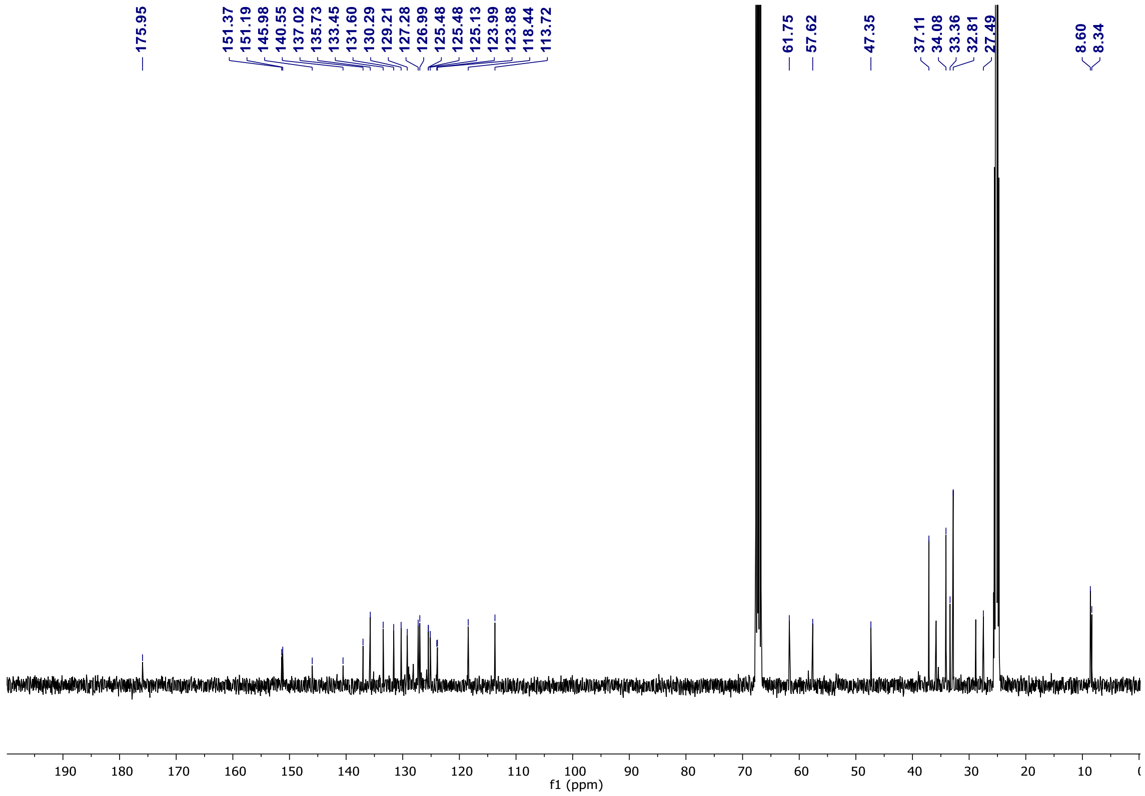

Figure S42. ^{13}C{^{1}H} NMR spectrum of **10** in THF-d_8.

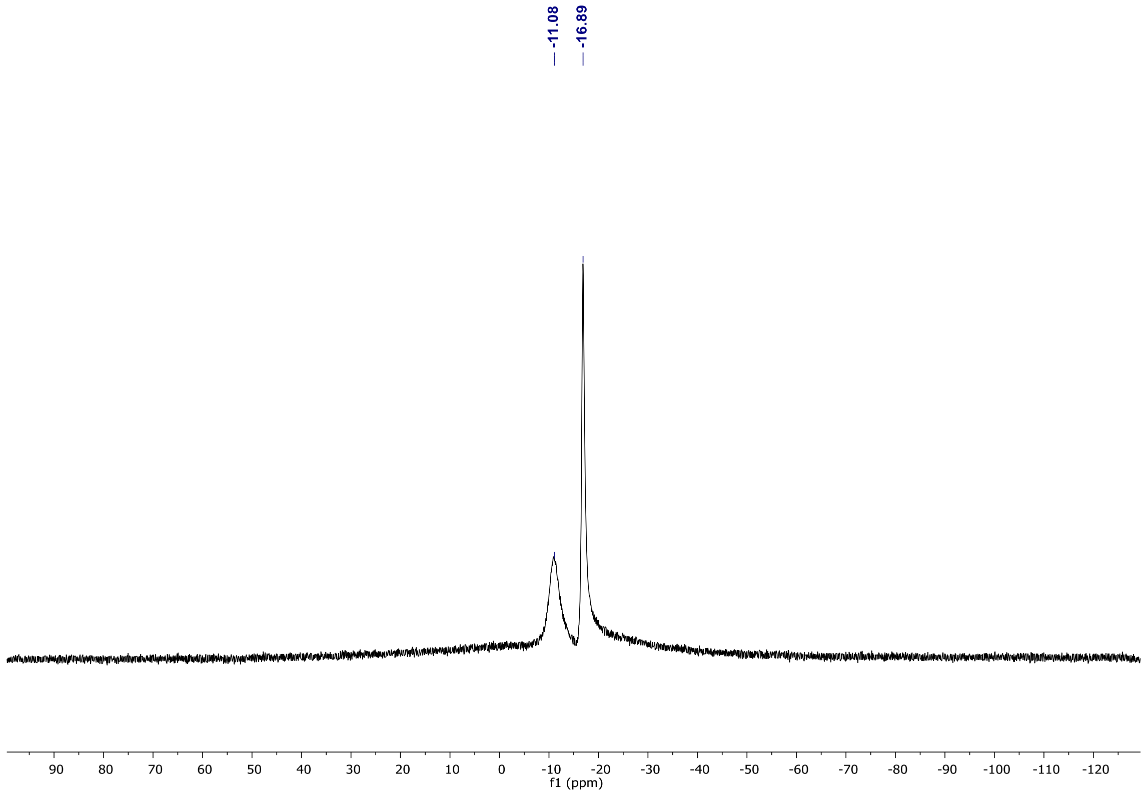

Figure S43. $^{11}B\{^1H\}$ NMR spectrum of **10** in THF-d_8.

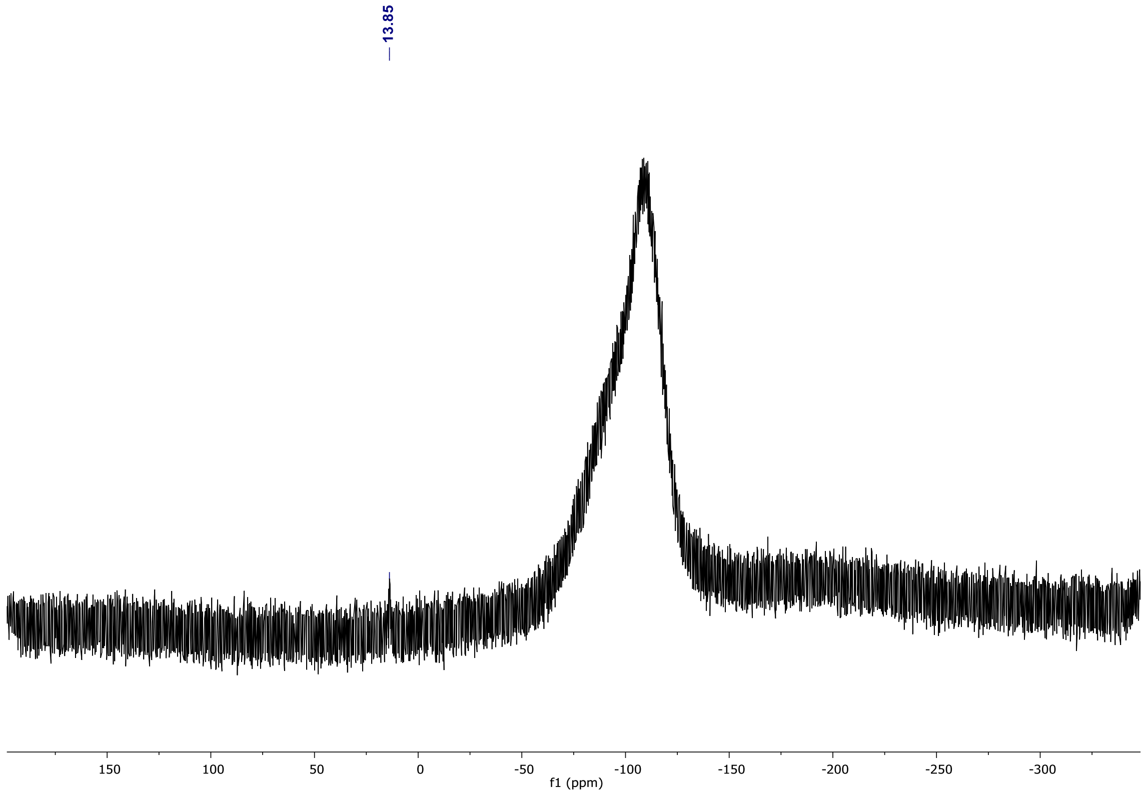

Figure S44. $^{29}Si\{^1H\}$ NMR spectrum of **10** in THF-d_8.

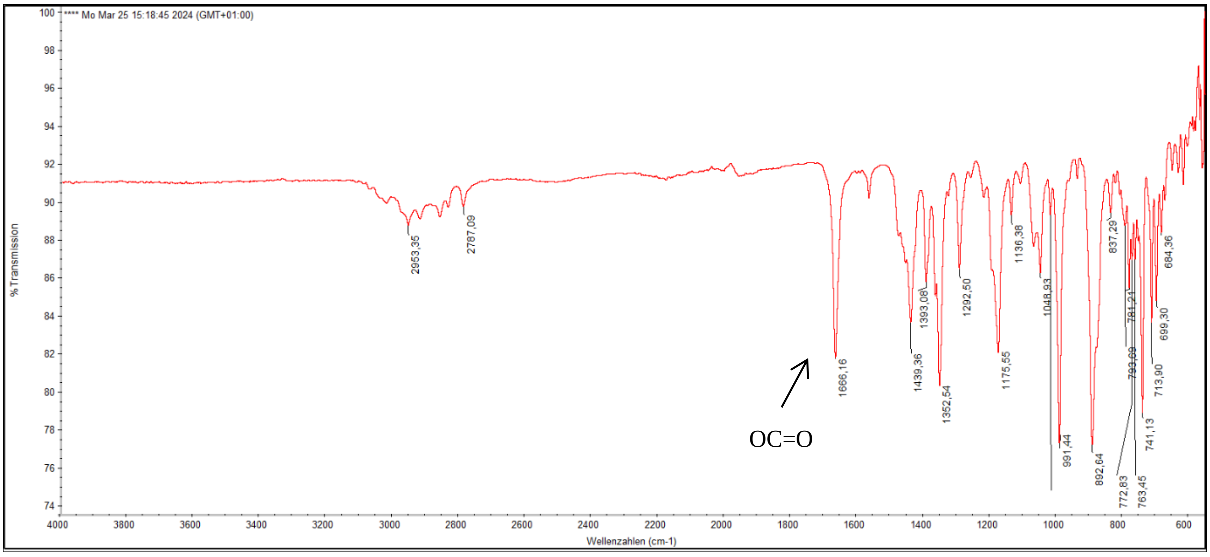

Figure S45. IR spectrum of **10**.

C. X-ray Crystallographic Data

Table S1. Crystal data and structure refinement for **1**.

Empirical formula	C51 H75 B2 Br4 N6 O2 Si2	
Formula weight	1201.61	
Temperature	149.96 K	
Wavelength	1.54184 Å	
Crystal system	Monoclinic	
Space group	I 1 2/a 1	
Unit cell dimensions	a = 17.9591(5) Å	a= 90°.
	b = 15.1327(4) Å	b= 95.480(2)°.
	c = 43.1136(9) Å	g = 90°.
Volume	11663.4(5) Å^3	
Z	8	
Density (calculated)	1.369 Mg/m^3	
Absorption coefficient	4.081 mm^{-1}	
F(000)	4936	
Crystal size	0.05 x 0.04 x 0.02 mm^3	
Theta range for data collection	3.097 to 72.542°.	
Index ranges	-21<=h<=18, -17<=k<=18, -53<=l<=49	
Reflections collected	23968	
Independent reflections	11293 [R(int) = 0.0489]	
Completeness to theta = 67.684°	99.8 %	
Absorption correction	Semi-empirical from equivalents	
Max. and min. transmission	1.00000 and 0.17552	
Refinement method	Full-matrix least-squares on F^2	
Data / restraints / parameters	11293 / 9 / 626	
Goodness-of-fit on F^2	0.985	
Final R indices [I>2sigma(I)]	R1 = 0.0398, wR2 = 0.0862	
R indices (all data)	R1 = 0.0578, wR2 = 0.0948	
Extinction coefficient	n/a	
Largest diff. peak and hole	0.531 and -0.445 e.Å^{-3}	

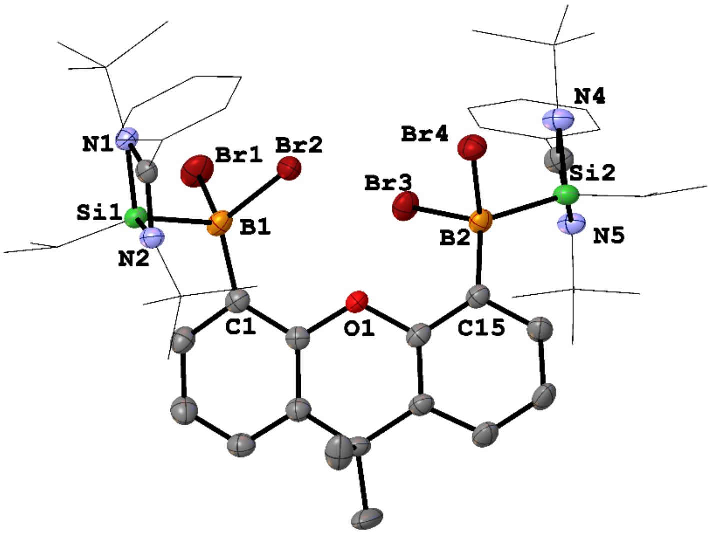

Figure S46. Molecular structure of compound **1**. Thermal ellipsoids are drawn at the 50% probability level. H atoms and solvent DME are omitted for clarity.

Table S2. Selected interatomic distances and angles of compound **1.**

Bond lengths [Å]		Angles [°]	
Br(2)-B(1)	2.035(4)	Br(2)-B(1)-Br(1)	108.75(18)
Br(1)-B(1)	2.074(3)	Br(2)-B(1)-Si(1)	101.47(16)
Br(4)-B(2)	2.080(4)	Si(1)-B(1)-Br(1)	107.63(15)
Br(3)-B(2)	2.058(3)	Br(3)-B(2)-Br(4)	109.41(16)
Si(1)-B(1)	2.046(4)	Si(2)-B(2)-Br(4)	104.42(17)
Si(2)-B(2)	2.032(4)	Si(2)-B(2)-Br(3)	102.01(15)
O(1)-C(6)	1.379(4)		
O(1)-C(10)	1.377(4)		
C(1)-B(1)	1.597(5)		
C(15)-B(2)	1.609(5)		

Table S3. Crystal data and structure refinement for **2**.

Empirical formula	C56 H78 B2 N6 O Si2	
Formula weight	929.04	
Temperature	110.49(10) K	
Wavelength	1.54184 Å	
Crystal system	Monoclinic	
Space group	P 1 21/c 1	
Unit cell dimensions	a = 15.6923(3) Å	a= 90°.
	b = 15.5327(3) Å	b= 90.791(2)°.
	c = 21.9776(4) Å	g = 90°.
Volume	5356.39(18) $Å^3$	
Z	4	
Density (calculated)	1.152 Mg/m^3	
Absorption coefficient	0.930 mm^{-1}	
F(000)	2008	
Crystal size	0.04 x 0.02 x 0.01 mm^3	
Theta range for data collection	2.816 to 72.426°.	
Index ranges	-18<=h<=13, -18<=k<=18, -26<=l<=27	
Reflections collected	25490	
Independent reflections	10367 [R(int) = 0.0453]	
Completeness to theta = 67.684°	99.9 %	
Absorption correction	Semi-empirical from equivalents	
Max. and min. transmission	1.00000 and 0.82318	
Refinement method	Full-matrix least-squares on F^2	
Data / restraints / parameters	10367 / 0 / 623	
Goodness-of-fit on F^2	1.029	
Final R indices [I>2sigma(I)]	R1 = 0.0547, wR2 = 0.1402	
R indices (all data)	R1 = 0.0813, wR2 = 0.1591	
Extinction coefficient	n/a	
Largest diff. peak and hole	0.414 and -0.398 e.$Å^{-3}$	

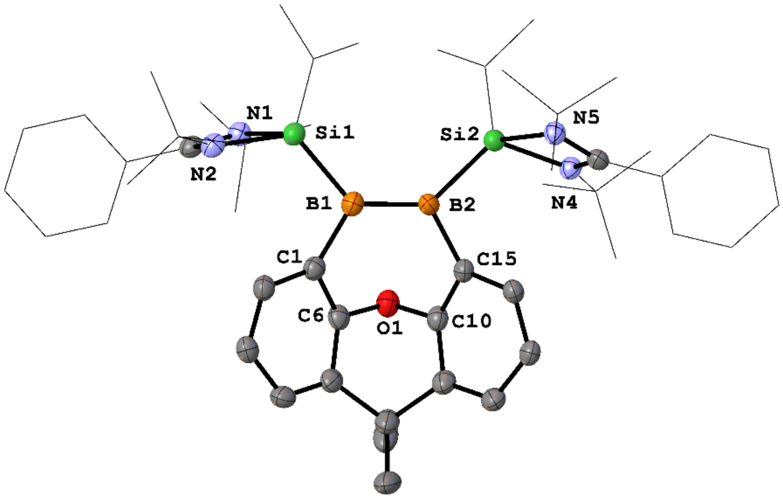

Figure S47. Molecular structure of compound **2**. Thermal ellipsoids are drawn at the 50% probability level. H atoms and solvent toluene are omitted for clarity.

Table S4. Selected interatomic distances and angles of compound **2.**

Bond lengths [Å]		Angles [°]	
Si(1)-B(1)	1.967(3)	C(1)-B(1)-Si(1)	109.30(17)
Si(2)-B(2)	1.975(3)	C(1)-B(1)-B(2)	120.4(2)
B(1)-B(2)	1.646(4)	C(15)-B(2)-Si(2)	106.46(17)
O(1)-C(6)	1.398(3)	C(15)-B(2)-B(1)	118.2(2)
O(1)-C(10)	1.401(3)	C(6)-O(1)-C(10)	99.28(17)
C(1)-B(1)	1.618(4)		
C(15)-B(2)	1.618(3)		

Table S5. Crystal data and structure refinement for **3**.

Empirical formula	C178.50 H246 B6 N18 O3 P12 Si6	
Formula weight	3296.96	
Temperature	149.99(10) K	
Wavelength	1.54184 Å	
Crystal system	Triclinic	
Space group	P-1	
Unit cell dimensions	a = 18.4505(7) Å	a= 113.394(5)°.
	b = 25.1155(12) Å	b= 108.062(4)°.
	c = 25.1189(13) Å	g = 97.606(3)°.
Volume	9711.6(9) $Å^3$	
Z	2	
Density (calculated)	1.127 Mg/m^3	
Absorption coefficient	1.748 mm^{-1}	
F(000)	3522	
Crystal size	0.09 x 0.06 x 0.03 mm^3	
Theta range for data collection	2.630 to 72.618°.	
Index ranges	-20<=h<=22, -30<=k<=30, -30<=l<=30	
Reflections collected	74908	
Independent reflections	37400 [R(int) = 0.1340]	
Completeness to theta = 67.684°	99.9 %	
Absorption correction	Semi-empirical from equivalents	
Max. and min. transmission	1.00000 and 0.49199	
Refinement method	Full-matrix least-squares on F^2	
Data / restraints / parameters	37400 / 6 / 1783	
Goodness-of-fit on F^2	0.864	
Final R indices [I>2sigma(I)]	R1 = 0.0690, wR2 = 0.1049	
R indices (all data)	R1 = 0.1548, wR2 = 0.1394	
Extinction coefficient	n/a	
Largest diff. peak and hole	0.276 and -0.291 e.$Å^{-3}$	

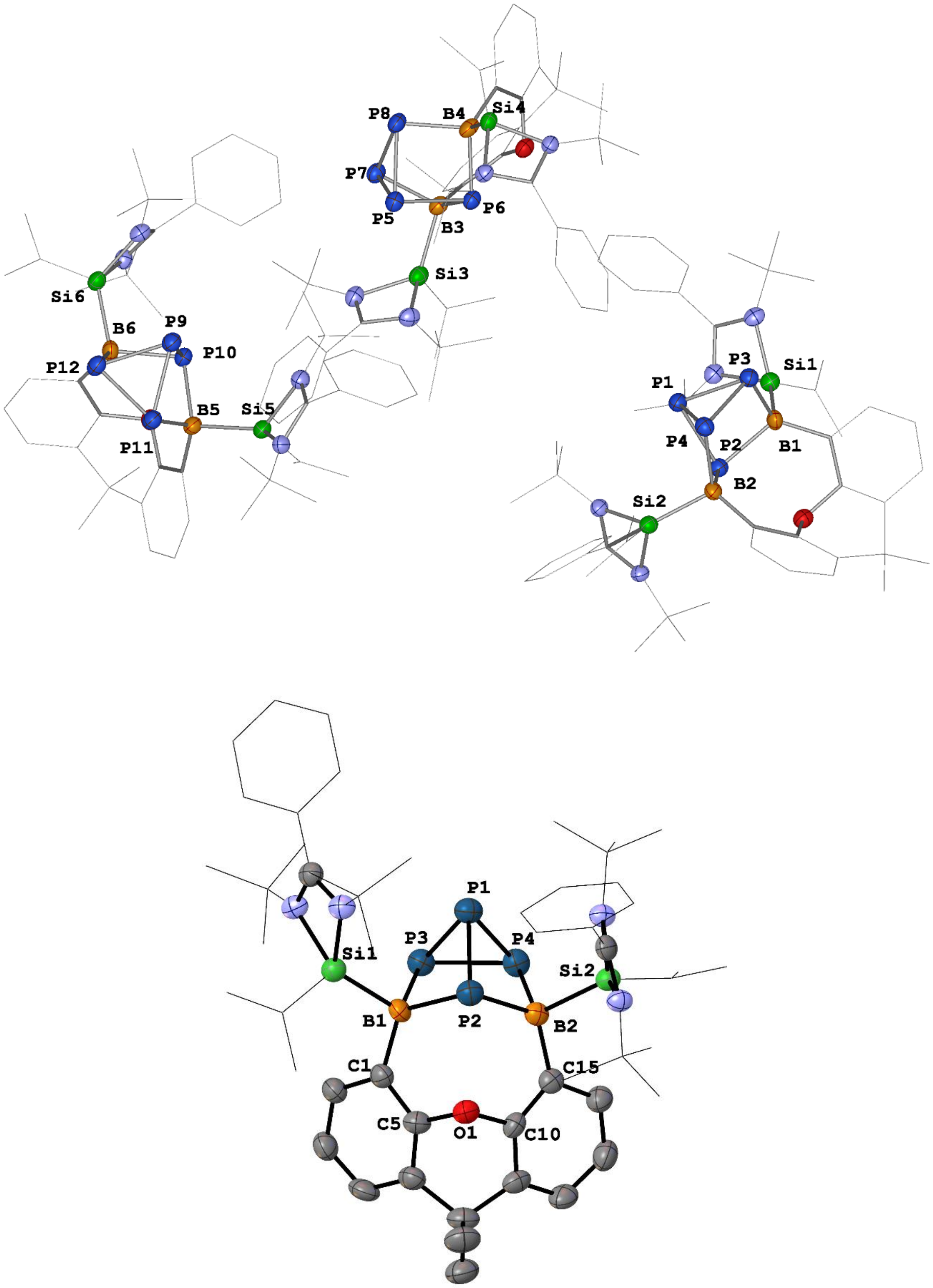

Figure S48. Molecular structure of compound **3** (Three different molecules are observed in the asymmetric unit; the bottom one is selected for showing in MS). Thermal ellipsoids are drawn at the 50% probability level. H atoms and solvent toluene are omitted for clarity.

Table S6. Selected interatomic distances and angles of compound **3.**

Bond lengths [Å]		Angles [°]	
P(2)-B(1)	2.036(6)	B(2)-P(2)-B(1)	103.7(2)
P(2)-B(2)	2.007(6)	Si(1)-B(1)-P(2)	105.1(3)
P(3)-B(1)	1.990(5)	C(1)-B(1)-P(2)	122.2(4)
P(4)-B(2)	1.991(5)	P(2)-B(2)-Si(2)	107.0(3)
Si(1)-B(1)	2.020(6)	C(15)-B(2)-P(2)	121.5(3)
Si(2)-B(2)	2.026(5)	B(3)-P(6)-B(4)	104.7(2)
P(6)-B(3)	2.032(6)	P(6)-B(3)-Si(3)	107.4(3)
P(6)-B(4)	2.048(5)	C(50)-B(3)-P(6)	123.0(4)
P(7)-B(3)	2.029(5)	Si(4)-B(4)-P(6)	106.7(3)
P(8)-B(4)	2.007(6)	C(64)-B(4)-P(6)	122.6(3)
Si(3)-B(3)	2.043(6)	B(5)-P(10)-B(6)	103.2(2)
Si(4)-B(4)	2.005(6)	P(10)-B(5)-Si(5)	106.0(3)
P(10)-B(5)	2.028(6)	C(99)-B(5)-P(10)	121.4(4)
P(10)-B(6)	2.035(6)	Si(6)-B(6)-P(10)	106.4(3)
P(11)-B(5)	2.004(5)	C(113)-B(6)-P(10)	122.2(3)
P(12)-B(6)	2.006(6)		
Si(5)-B(5)	2.055(6)		
Si(6)-B(6)	2.013(6)		

Table S7. Crystal data and structure refinement for **4**.

Empirical formula	C32 H44 B2 N4 O Si	
Formula weight	550.42	
Temperature	150.00 K	
Wavelength	1.54184 Å	
Crystal system	Monoclinic	
Space group	P 1 21/n 1	
Unit cell dimensions	a = 9.3843(3) Å	a= 90°.
	b = 16.8644(5) Å	b= 100.616(3)°.
	c = 20.4105(6) Å	g = 90°.
Volume	3174.89(17) $Å^3$	
Z	4	
Density (calculated)	1.152 Mg/m^3	
Absorption coefficient	0.876 mm^{-1}	
F(000)	1184	
Crystal size	0.09 x 0.07 x 0.05 mm^3	
Theta range for data collection	3.424 to 72.429°.	
Index ranges	-11<=h<=11, -20<=k<=20, -18<=l<=25	
Reflections collected	22715	
Independent reflections	6209 [R(int) = 0.0553]	
Completeness to theta = 67.684°	100.0 %	
Absorption correction	Semi-empirical from equivalents	
Max. and min. transmission	1.00000 and 0.24966	
Refinement method	Full-matrix least-squares on F^2	
Data / restraints / parameters	6209 / 0 / 371	
Goodness-of-fit on F^2	1.041	
Final R indices [I>2sigma(I)]	R1 = 0.0653, wR2 = 0.1734	
R indices (all data)	R1 = 0.0827, wR2 = 0.1884	
Extinction coefficient	n/a	
Largest diff. peak and hole	0.632 and -0.246 e.$Å^{-3}$	

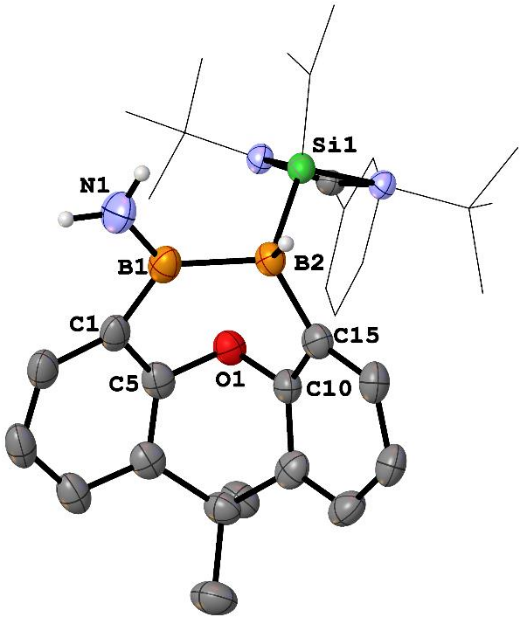

Figure S49. Molecular structure of compound **4**. Thermal ellipsoids are drawn at the 50% probability level. H atoms are omitted for clarity.

Table S8. Selected interatomic distances and angles of compound **4.**

Bond lengths [Å]		Angles [°]	
B(1)-B(2)	1.757(5)	N(1)-B(1)-B(2)	120.0(3)
N(1)-B(1)	1.406(4)	N(1)-B(1)-C(1)	115.2(3)
C(1)-B(1)	1.595(5)	C(10)-O(1)-C(5)	102.80(19)
O(1)-C(5)	1.389(3)	C(15)-B(2)-Si(1)	112.37(18)
O(1)-C(10)	1.387(3)	C(15)-B(2)-B(1)	116.0(2)
C(15)-B(2)	1.614(4)	B(1)-B(2)-Si(1)	106.16(19)
Si(1)-B(2)	1.999(3)		

Table S9. Crystal data and structure refinement for **5**.

Empirical formula	C49 H70 B2 N6 O Si2	
Formula weight	836.91	
Temperature	150.00 K	
Wavelength	1.54184 Å	
Crystal system	Monoclinic	
Space group	P 1 21/n 1	
Unit cell dimensions	a = 14.7595(4) Å	a= 90°.
	b = 16.8752(4) Å	b= 109.297(3)°.
	c = 20.2261(6) Å	g = 90°.
Volume	4754.7(2) $Å^3$	
Z	4	
Density (calculated)	1.169 Mg/m^3	
Absorption coefficient	0.994 mm^{-1}	
F(000)	1808	
Crystal size	0.09 x 0.07 x 0.04 mm^3	
Theta range for data collection	3.251 to 72.507°.	
Index ranges	-17<=h<=17, -20<=k<=20, -24<=l<=21	
Reflections collected	19062	
Independent reflections	9175 [R(int) = 0.0287]	
Completeness to theta = 67.684°	99.8 %	
Absorption correction	Semi-empirical from equivalents	
Max. and min. transmission	1.00000 and 0.86081	
Refinement method	Full-matrix least-squares on F^2	
Data / restraints / parameters	9175 / 1 / 559	
Goodness-of-fit on F^2	1.013	
Final R indices [I>2sigma(I)]	R1 = 0.0442, wR2 = 0.1102	
R indices (all data)	R1 = 0.0616, wR2 = 0.1214	
Extinction coefficient	n/a	
Largest diff. peak and hole	0.326 and -0.322 e.$Å^{-3}$	

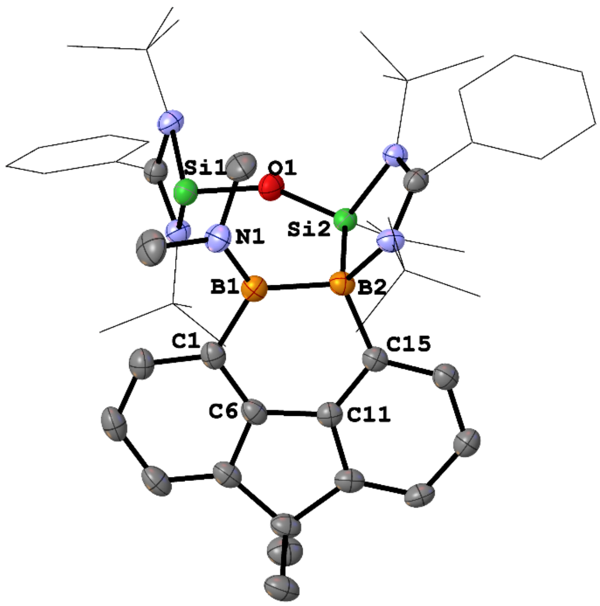

Figure S50. Molecular structure of compound **5**. Thermal ellipsoids are drawn at the 50% probability level. H atoms are omitted for clarity.

Table S10. Selected interatomic distances and angles of compound **5.**

Bond lengths [Å]		Angles [°]	
B(1)-B(2)	1.743(3)	Si(2)-O(1)-Si(1)	151.49(9)
C(1)-B(1)	1.595(3)	O(1)-Si(2)-B(2)	116.37(8)
C(15)-B(2)	1.625(3)	C(15)-B(2)-Si(2)	111.53(13)
N(1)-B(1)	1.420(3)	C(15)-B(2)-B(1)	112.35(16)
Si(2)-B(2)	1.994(2)	B(1)-B(2)-Si(2)	105.03(13)
Si(1)-O(1)	1.6603(14)	C(1)-B(1)-B(2)	116.26(16)
Si(2)-O(1)	1.6305(14)	N(1)-B(1)-C(1)	118.95(18)
		N(1)-B(1)-B(2)	124.78(18)

Table S11. Crystal data and structure refinement for **6**.

Empirical formula	C106 H160 B4 N12 O4 Si4	
Formula weight	1822.05	
Temperature	150.00(10) K	
Wavelength	1.54184 Å	
Crystal system	Triclinic	
Space group	P-1	
Unit cell dimensions	a = 13.9908(8) Å	α= 94.192(4)°.
	b = 18.9683(8) Å	β= 101.491(4)°.
	c = 21.4863(10) Å	γ = 95.979(4)°.
Volume	5531.6(5) $Å^3$	
Z	2	
Density (calculated)	1.094 Mg/m^3	
Absorption coefficient	0.903 mm^{-1}	
F(000)	1976	
Crystal size	0.09 x 0.06 x 0.04 mm^3	
Theta range for data collection	3.308 to 72.437°.	
Index ranges	-16<=h<=17, -23<=k<=23, -26<=l<=26	
Reflections collected	42532	
Independent reflections	21296 [R(int) = 0.0646]	
Completeness to theta = 67.684°	99.8 %	
Absorption correction	Semi-empirical from equivalents	
Max. and min. transmission	1.00000 and 0.50604	
Refinement method	Full-matrix least-squares on F^2	
Data / restraints / parameters	21296 / 99 / 1130	
Goodness-of-fit on F^2	0.955	
Final R indices [I>2sigma(I)]	R1 = 0.0592, wR2 = 0.1402	
R indices (all data)	R1 = 0.1048, wR2 = 0.1676	
Extinction coefficient	n/a	
Largest diff. peak and hole	0.234 and -0.349 e.$Å^{-3}$	

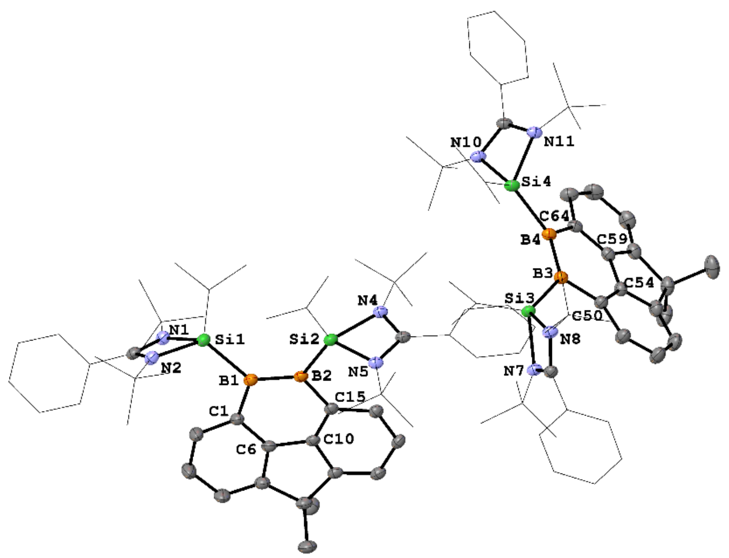

Figure S51. Molecular structure of compound **6** (Two different molecules are observed in the asymmetric unit). Thermal ellipsoids are drawn at the 50% probability level. H atoms and solvent DME are omitted for clarity.

Table S12. Selected interatomic distances and angles of compound **6.**

Bond lengths [Å]		Angles [°]	
B(1)-B(2)	1.650(4)	C(1)-B(1)-Si(1)	112.0(2)
B(3)-B(4)	1.652(4)	C(1)-B(1)-B(2)	116.4(2)
Si(1)-B(1)	1.985(3)	B(2)-B(1)-Si(1)	131.4(2)
Si(2)-B(2)	1.988(3)	C(15)-B(2)-Si(2)	112.4(2)
Si(3)-B(3)	1.995(3)	C(15)-B(2)-B(1)	116.7(2)
Si(4)-B(4)	2.000(3)	B(1)-B(2)-Si(2)	130.8(2)
C(6)-C(10)	1.431(4)	C(50)-B(3)-Si(3)	110.63(19)
C(54)-C(59)	1.425(4)	C(50)-B(3)-B(4)	116.8(2)
		B(4)-B(3)-Si(3)	131.6(2)
		C(64)-B(4)-Si(4)	111.90(19)
		C(64)-B(4)-B(3)	115.9(2)
		B(3)-B(4)-Si(4)	131.7(2)

Table S13. Crystal data and structure refinement for **7**.

Empirical formula	C34 H58 N6 O2 Si2	
Formula weight	639.04	
Temperature	150.01 K	
Wavelength	1.54184 Å	
Crystal system	Monoclinic	
Space group	P 1 21/n 1	
Unit cell dimensions	a = 9.7878(3) Å	a= 90°.
	b = 14.4273(3) Å	b= 108.019(3)°.
	c = 13.5027(4) Å	g = 90°.
Volume	1813.22(9) $Å^3$	
Z	2	
Density (calculated)	1.170 Mg/m^3	
Absorption coefficient	1.178 mm^{-1}	
F(000)	696	
Crystal size	0.05 x 0.03 x 0.02 mm^3	
Theta range for data collection	4.610 to 72.470°.	
Index ranges	-11<=h<=12, -16<=k<=17, -16<=l<=15	
Reflections collected	12715	
Independent reflections	3566 [R(int) = 0.0304]	
Completeness to theta = 67.684°	100.0 %	
Absorption correction	Semi-empirical from equivalents	
Max. and min. transmission	1.00000 and 0.24687	
Refinement method	Full-matrix least-squares on F^2	
Data / restraints / parameters	3566 / 0 / 207	
Goodness-of-fit on F^2	1.041	
Final R indices [I>2sigma(I)]	R1 = 0.0362, wR2 = 0.0947	
R indices (all data)	R1 = 0.0413, wR2 = 0.0995	
Extinction coefficient	n/a	
Largest diff. peak and hole	0.279 and -0.364 e.$Å^{-3}$	

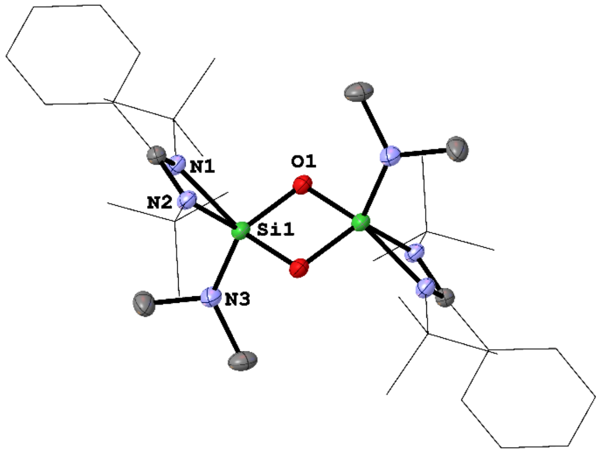

Figure S52. Molecular structure of compound **7**. Thermal ellipsoids are drawn at the 50% probability level. H atoms and solvent toluene are omitted for clarity.

Table S14. Selected interatomic distances and angles of compound **7.**

Bond lengths [Å]		Angles [°]	
Si(1)-O(1)	1.6662(9)	O(1)-Si(1)-O(1)#1	85.07(5)
Si(1)-O(1)#1	1.7242(9)	O(1)-Si(1)-N(1)	90.54(5)
Si(1)-N(1)	2.0066(11)	O(1)#1-Si(1)-N(1)	161.46(5)
Si(1)-N(2)	1.8338(11)	O(1)-Si(1)-N(2)	120.03(5)
Si(1)-N(3)	1.7266(12)	O(1)#1-Si(1)-N(2)	99.00(5)
		O(1)-Si(1)-N(3)	122.74(5)
		O(1)#1-Si(1)-N(3)	100.72(5)

Table S15. Crystal data and structure refinement for **8**.

Empirical formula	C85 H114 B4 N10 Si2	
Formula weight	1375.28	
Temperature	150.00(10) K	
Wavelength	1.54184 Å	
Crystal system	Monoclinic	
Space group	P 1 21/c 1	
Unit cell dimensions	a = 19.4845(7) Å	a= 90°.
	b = 19.8475(9) Å	b= 98.236(3)°.
	c = 21.2537(4) Å	g = 90°.
Volume	8134.4(5) Å^3	
Z	4	
Density (calculated)	1.123 Mg/m^3	
Absorption coefficient	0.765 mm^{-1}	
F(000)	2968	
Crystal size	0.13 x 0.1 x 0.08 mm^3	
Theta range for data collection	3.061 to 72.479°.	
Index ranges	-23<=h<=22, -16<=k<=24, -22<=l<=26	
Reflections collected	34908	
Independent reflections	15738 [R(int) = 0.0613]	
Completeness to theta = 67.684°	99.9 %	
Absorption correction	Semi-empirical from equivalents	
Max. and min. transmission	1.00000 and 0.85129	
Refinement method	Full-matrix least-squares on F^2	
Data / restraints / parameters	15738 / 68 / 906	
Goodness-of-fit on F^2	1.013	
Final R indices [I>2sigma(I)]	R1 = 0.0793, wR2 = 0.2041	
R indices (all data)	R1 = 0.1590, wR2 = 0.2666	
Extinction coefficient	n/a	
Largest diff. peak and hole	0.229 and -0.269 e.Å^{-3}	

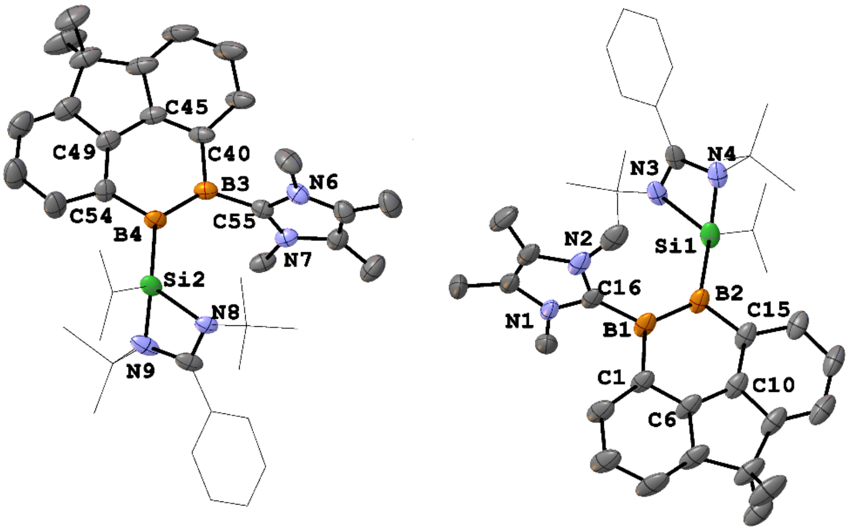

Figure S53. Molecular structure of compound **8** (Two different molecules are observed in the asymmetric unit). Thermal ellipsoids are drawn at the 50% probability level. H atoms are omitted for clarity.

Table S16. Selected interatomic distances and angles of compound **8.**

Bond lengths [Å]		Angles [°]	
B(2)-B(1)	1.623(7)	C(16)-B(1)-B(2)	129.5(4)
B(4)-B(3)	1.603(6)	C(1)-B(1)-C(16)	112.2(4)
Si(1)-B(2)	1.968(4)	C(1)-B(1)-B(2)	118.1(4)
Si(2)-B(4)	1.965(4)	C(15)-B(2)-Si(1)	117.3(3)
C(16)-B(1)	1.584(6)	C(15)-B(2)-B(1)	117.2(4)
C(55)-B(3)	1.599(6)	B(1)-B(2)-Si(1)	125.1(3)
C(10)-C(6)	1.419(6)	C(55)-B(3)-B(4)	130.3(3)
C(49)-C(45)	1.412(6)	C(40)-B(3)-C(55)	111.7(3)
		C(40)-B(3)-B(4)	118.0(4)
		C(54)-B(4)-Si(2)	116.6(3)
		C(54)-B(4)-B(3)	117.1(3)
		B(3)-B(4)-Si(2)	125.8(3)

Table S17. Crystal data and structure refinement for **9**.

Empirical formula	C49.50 H52 B4 N4 O2	
Formula weight	778.19	
Temperature	149.91(19) K	
Wavelength	1.54184 Å	
Crystal system	Monoclinic	
Space group	P 1 21/c 1	
Unit cell dimensions	a = 17.5116(8) Å	a= 90°.
	b = 21.4542(13) Å	b= 90.807(4)°.
	c = 11.2933(7) Å	g = 90°.
Volume	4242.4(4) $Å^3$	
Z	4	
Density (calculated)	1.218 Mg/m^3	
Absorption coefficient	0.561 mm^{-1}	
F(000)	1652	
Crystal size	0.07 x 0.02 x 0.01 mm^3	
Theta range for data collection	2.523 to 72.922°.	
Index ranges	-18<=h<=21, -26<=k<=26, -13<=l<=13	
Reflections collected	30744	
Independent reflections	8297 [R(int) = 0.1016]	
Completeness to theta = 67.684°	100.0 %	
Absorption correction	Semi-empirical from equivalents	
Max. and min. transmission	1.00000 and 0.28538	
Refinement method	Full-matrix least-squares on F^2	
Data / restraints / parameters	8297 / 0 / 517	
Goodness-of-fit on F^2	1.011	
Final R indices [I>2sigma(I)]	R1 = 0.0677, wR2 = 0.1665	
R indices (all data)	R1 = 0.1163, wR2 = 0.2008	
Extinction coefficient	n/a	
Largest diff. peak and hole	0.247 and -0.458 e.$Å^{-3}$	

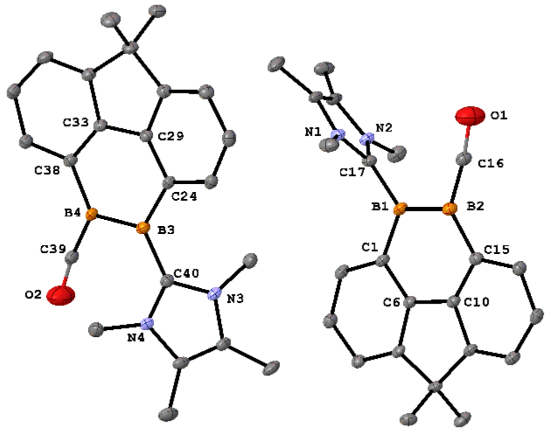

Figure S54. Molecular structure of compound **9** (Two different molecules are observed in the asymmetric unit). Thermal ellipsoids are drawn at the 50% probability level. H atoms are omitted for clarity.

Table S18. Selected interatomic distances and angles of compound **9.**

Bond lengths [Å]		Angles [°]	
B(1)-B(2)	1.629(5)	C(1)-B(1)-B(2)	116.4(2)
B(3)-B(4)	1.633(5)	C(1)-B(1)-C(17)	118.6(3)
B(1)-C(17)	1.592(4)	C(17)-B(1)-B(2)	125.0(3)
B(3)-C(40)	1.574(4)	C(15)-B(2)-B(1)	120.1(3)
B(2)-C(16)	1.441(5)	C(16)-B(2)-B(1)	110.6(3)
B(4)-C(39)	1.435(5)	C(16)-B(2)-C(15)	129.2(3)
C(6)-C(10)	1.443(4)	C(24)-B(3)-B(4)	114.8(3)
C(29)-C(33)	1.445(4)	C(24)-B(3)-C(40)	119.6(3)
		C(40)-B(3)-B(4)	125.6(3)
		C(38)-B(4)-B(3)	121.3(3)
		C(39)-B(4)-B(3)	111.4(3)
		C(39)-B(4)-C(38)	127.3(3)

Table S19. Crystal data and structure refinement for **10**.

Empirical formula	C136 H199 B6 N15 O10 Si3	
Formula weight	2353.22	
Temperature	150.00(10) K	
Wavelength	1.54184 Å	
Crystal system	Triclinic	
Space group	P-1	
Unit cell dimensions	a = 19.2103(8) Å	a= 69.336(4)°.
	b = 19.2124(7) Å	b= 87.779(4)°.
	c = 20.8943(9) Å	g = 74.530(4)°.
Volume	6940.9(5) $Å^3$	
Z	2	
Density (calculated)	1.126 Mg/m^3	
Absorption coefficient	0.782 mm^{-1}	
F(000)	2544	
Crystal size	0.17 x 0.08 x 0.05 mm^3	
Theta range for data collection	2.391 to 72.975°.	
Index ranges	-22<=h<=23, -23<=k<=20, -25<=l<=25	
Reflections collected	52829	
Independent reflections	26802 [R(int) = 0.0717]	
Completeness to theta = 67.684°	99.8 %	
Absorption correction	Semi-empirical from equivalents	
Max. and min. transmission	1.00000 and 0.18884	
Refinement method	Full-matrix least-squares on F^2	
Data / restraints / parameters	26802 / 0 / 1393	
Goodness-of-fit on F^2	1.020	
Final R indices [I>2sigma(I)]	R1 = 0.0827, wR2 = 0.2197	
R indices (all data)	R1 = 0.1230, wR2 = 0.2522	
Extinction coefficient	n/a	
Largest diff. peak and hole	0.606 and -0.533 e.$Å^{-3}$	

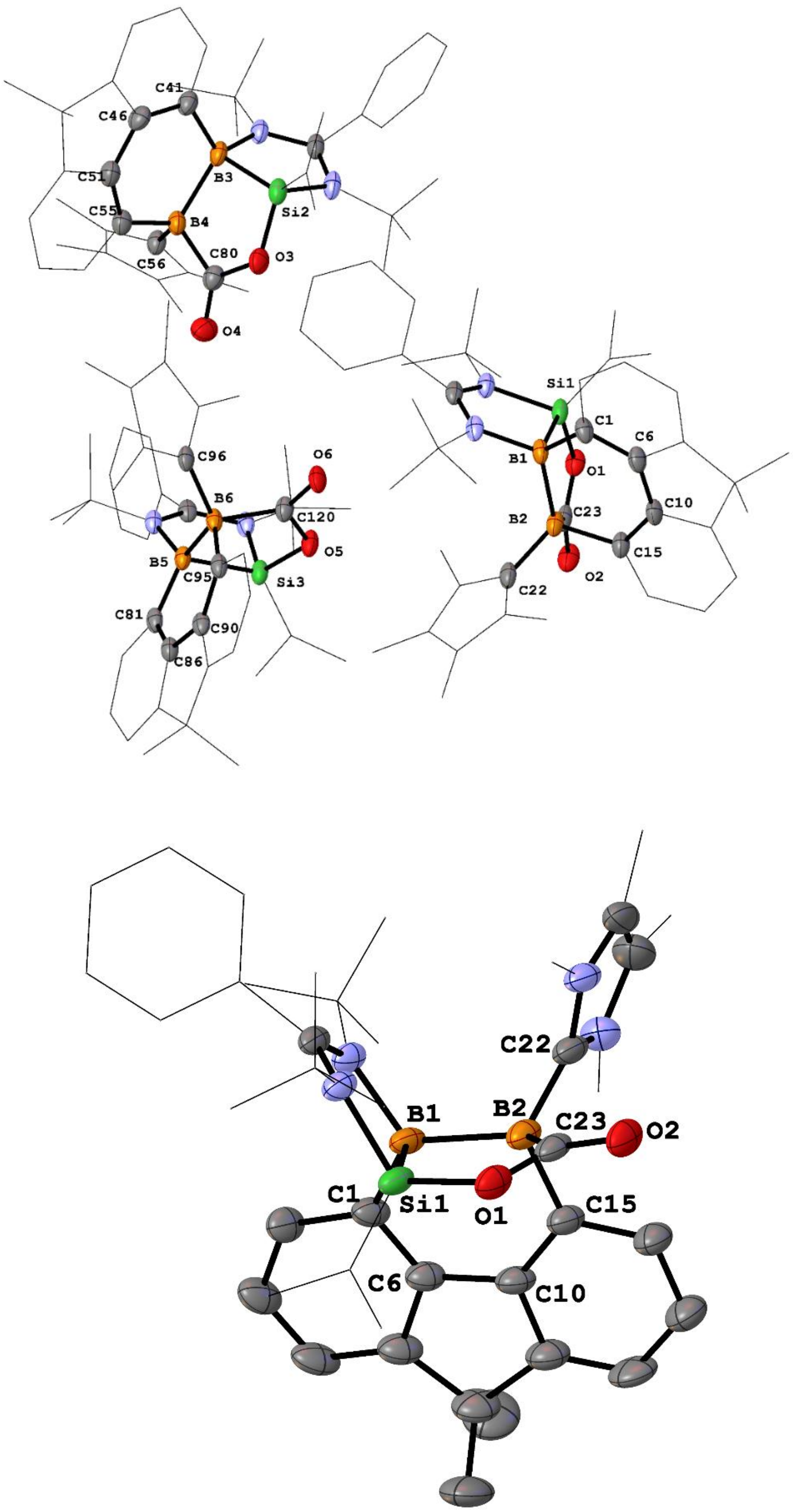

Figure S55. Molecular structure of compound **10** (Three different molecules are observed in the asymmetric unit; the bottom one is selected for showing in MS). Thermal ellipsoids are drawn at the 50% probability level. H atoms are omitted for clarity.

Table S20. Selected interatomic distances and angles of compound **10.**

Bond lengths [Å]		Angles [°]	
Si(1)-O(1)	1.681(2)	O(1)-Si(1)-B(1)	107.79(15)
Si(1)-B(1)	1.956(4)	B(2)-B(1)-Si(1)	92.3(2)
B(1)-B(2)	1.846(5)	C(22)-B(2)-B(1)	118.2(3)
C(22)-B(2)	1.619(6)	C(23)-B(2)-B(1)	110.0(3)
C(23)-B(2)	1.634(5)	C(15)-B(2)-B(1)	109.0(3)
O(2)-C(23)	1.229(4)	O(1)-C(23)-B(2)	116.0(3)
O(1)-C(23)	1.377(5)	O(2)-C(23)-B(2)	127.4(4)
C(1)-B(1)	1.601(4)	O(2)-C(23)-O(1)	116.1(3)
C(1)-C(6)	1.394(5)	C(23)-O(1)-Si(1)	111.73(19)
C(6)-C(10)	1.444(5)	C(6)-C(1)-B(1)	118.2(3)
C(10)-C(15)	1.394(5)	C(1)-C(6)-C(10)	126.3(3)
C(15)-B(2)	1.645(5)	C(15)-C(10)-C(6)	127.1(3)
Si(2)-O(3)	1.669(3)	C(10)-C(15)-B(2)	121.3(3)
Si(2)-B(3)	1.965(4)	O(3)-Si(2)-B(3)	108.45(16)
B(3)-B(4)	1.858(6)	B(4)-B(3)-Si(2)	91.6(2)
C(56)-B(4)	1.601(5)	C(55)-B(4)-B(3)	109.7(3)
C(80)-B(4)	1.645(5)	C(56)-B(4)-B(3)	117.7(3)
O(3)-C(80)	1.403(4)	C(80)-B(4)-B(3)	111.1(3)
O(4)-C(80)	1.203(4)	O(3)-C(80)-B(4)	113.9(3)
C(41)-B(3)	1.596(5)	O(4)-C(80)-B(4)	129.8(3)
C(41)-C(46)	1.409(5)	O(4)-C(80)-O(3)	115.9(3)
C(46)-C(51)	1.442(6)	C(80)-O(3)-Si(2)	112.0(2)

C(51)-C(55)	1.389(5)	C(46)-C(41)-B(3)	117.7(3)
C(55)-B(4)	1.646(5)	C(41)-C(46)-C(51)	127.1(3)
Si(3)-O(5)	1.672(2)	C(55)-C(51)-C(46)	127.2(3)
Si(3)-B(5)	1.958(3)	C(51)-C(55)-B(4)	120.8(3)
B(6)-B(5)	1.852(5)	O(5)-Si(3)-B(5)	108.21(13)
C(96)-B(6)	1.607(5)	B(6)-B(5)-Si(3)	92.46(18)
C(120)-B(6)	1.652(5)	C(96)-B(6)-B(5)	116.8(3)
O(5)-C(120)	1.390(4)	C(96)-B(6)-C(120)	106.9(3)
O(6)-C(120)	1.211(4)	C(96)-B(6)-C(95)	113.8(3)
C(81)-B(5)	1.588(5)	O(5)-C(120)-B(6)	115.3(3)
C(86)-C(81)	1.392(5)	O(6)-C(120)-B(6)	128.1(3)
C(90)-C(86)	1.476(4)	O(6)-C(120)-O(5)	116.1(3)
C(95)-C(90)	1.400(5)	C(120)-O(5)-Si(3)	111.52(19)
C(95)-B(6)	1.626(6)	C(86)-C(81)-B(5)	118.2(3)
		C(81)-C(86)-C(90)	125.9(3)
		C(95)-C(90)-C(86)	125.9(3)
		C(90)-C(95)-B(6)	121.7(3)

F. Theoretical Calculations

Geometry optimizations were performed with the Gaussian 16 program.[4] The calculations were carried out for all molecules using the BP86[5, 6] functional with def2-TZVP[7, 8, 9] basis set and dispersion correction by Grimme with Becke-Johnson damping D3(BJ)[10] (termed as BP86-D3(BJ)/def2-TZVP). Partial charges q were obtained from a single-point calculation of the molecules at the BP86/def2-TZVP level by using NBO 7.0[11, 12] as implemented with Gaussian 16 program. The calculated Mayer[13, 14, 15] bond orders P was analyzed by using Multiwfn.[16] NICS calculations were carried out with Multiwfn. The structures were illustrated by CYLview.[17]

The bonding situation was analyzed by means of an energy decomposition analysis (EDA)[18, 19] together with the natural orbitals for chemical valence (NOCV)[20, 21] method by using the ADF 2022 program package.[22, 23] The EDA-NOCV calculations were carried out at the BP86-D3(BJ)/TZ2P level[24] using the BP86/def2-TZVP optimized geometries. In this analysis, the intrinsic interaction energy (ΔE_{int}) between two fragments can be divided into four energy components as follows:

$$\Delta E_{int} = \Delta E_{elstat} + \Delta E_{pauli} + \Delta E_{orb} + \Delta E_{disp} \qquad (1)$$

While the electrostatic ΔE_{elstat} term represents the quasiclassical electrostatic interaction between the unperturbed charge distributions of the prepared fragments, the Pauli repulsion ΔE_{Pauli} corresponds to the energy change associated with the transformation from the superposition of the unperturbed electron densities of the isolated fragments to the wavefunction,[25] which properly obeys the Pauli principle through explicit antisymmetrization and renormalization of the production wavefunction. The orbital term ΔE_{orb} can be further decomposed into contributions from each irreducible representation of the point group of the interacting system as follows:

$$\Delta E_{orb} = \sum_r \Delta E_r \qquad (2)$$

The addition of ΔE_{prep} to the intrinsic interaction energy ΔE_{int} gives the total energy ΔE, which has opposite sign compared with the bond dissociation energy D_e [Eq. (3)]

$$\Delta E_{(-D_e)} = \Delta E_{int} + \Delta E_{prep} \qquad (3)$$

The combination of the EDA with NOCV enables the partition of the total orbital interactions into pairwise contributions of the orbital interactions which is very vital to get a complete picture of the bonding. The charge deformation $\Delta\rho_k(r)$, resulting from the mixing of the orbital pairs $\psi_k(r)$ and $\psi_{-k}(r)$ of the interacting fragments presents the amount and the shape of the charge flow due to the orbital interactions [Eq. (4)], and the associated energy term ΔE_{orb} provides with the size of stabilizing orbital energy originated from such interaction [Eq. (5)].

$$\Delta\rho_{orb}(r) = \sum_k \Delta\rho_k(r) = \sum_{K=1}^{N/2} V_K \left[-\psi^2_{-K}(r) + \psi^2_k(r)\right] \qquad (4)$$

$$\Delta E_{orb} = \sum_k \Delta E_k^{orb} = \sum_{K=1}^{N/2} V_K\left[-F^{TS}_{-K,-K} + F^{TS}_{k,K}\right] \qquad (5)$$

More details about the EDA-NOCV method and its application are given in recent reviews articles.[26, 27, 28, 2]

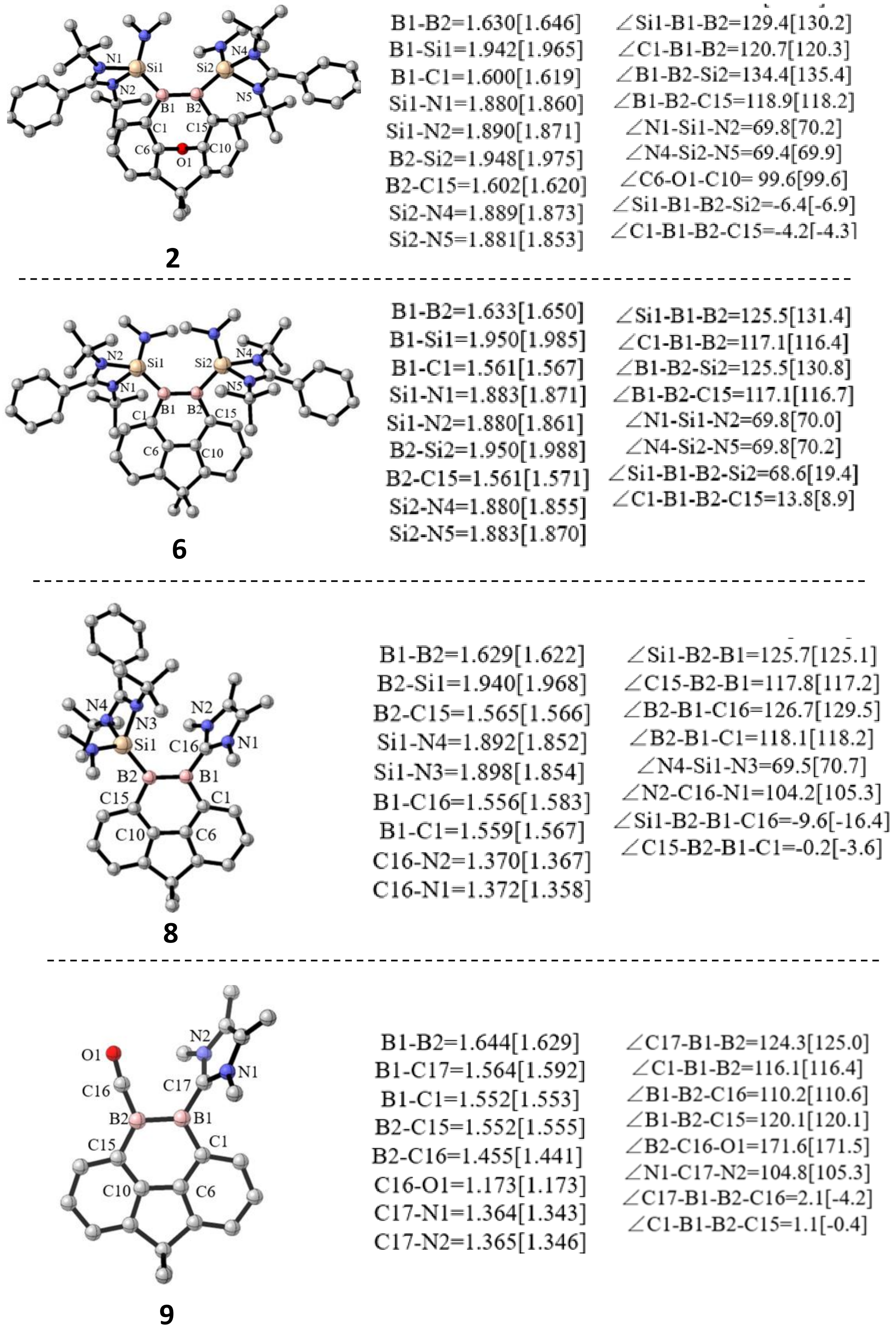

Figure S56. Optimized geometries of the compounds **2, 6, 8, 9** at the BP86-D3(BJ)/def2-TZVP levels. Selected bond lengths in Å, the experimental values are given in brackets. Hydrogen atoms are omitted for clarity (color code, C: gray, N: blue, B: pink, Si: yellow, O: red).

Table S21. The calculated NBO charges (q) and Mayer bond orders (P) of compounds **2**, **6**, **8** and **9** at the BP86-D3(BJ)/def2-TZVP level.

	**Partial charge q**		**Mayer bond order P**	
**2**	B1	-0.52	B1-B2	1.55
	B2	-0.51	B1-Si1	0.70
	Si1	1.91	B1-C1	0.86
	Si2	1.91	B2-C15	0.89
	C1	-0.29	B2-Si2	0.69
	C15	-0.28		
	SiN_3R(B1)	0.40		
	SiN_3R(B2)	0.39		
**6**	B1	-0.48	B1-B2	1.42
	B2	-0.48	B1-Si1	0.79
	Si1	1.89	B1-C1	0.96
	Si2	1.89	B2-C15	0.96
	C1	-0.28	B2-Si2	0.79
	C15	-0.28		
	SiN_3R(B1)	0.43		
	SiN_3R(B2)	0.43		
**8**	B1	0.05	B1-B2	1.34
	B2	-0.59	B1-C16	1.16
	C15	-0.25	B1-C1	1.04
	C1	-0.27	B2-C15	0.98
	Si1	1.90	B2-Si1	0.72
	C16	0.25		
	SiN_3R(B1)	0.48		
	CN_2R(B2)	0.29		
**9**	B1	0.21	B1-B2	1.12
	B2	-0.24	B1-C1	1.08
	C1	-0.30	B1-C17	1.09
	C15	-0.20	B2-C15	1.05
	C17	0.22	B2-C16	1.41
	C16	0.55	C16-O1	2.10
	O1	-0.4		
	CN_2R(B1)	0.72		
	R(B2)	-0.15		

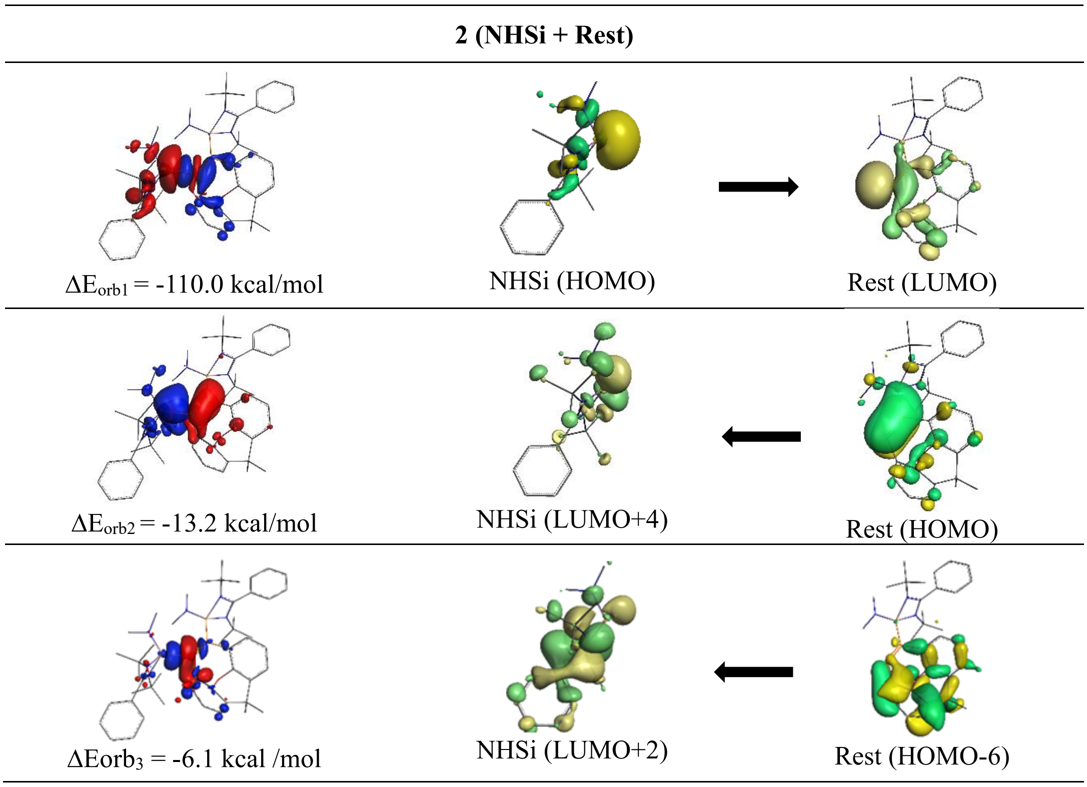

Figure S57. Plots of deformation densities Δρ of the pairwise orbital interactions and the shape of the most important interacting MOs of the chosen fragments of **2**. The direction of the charge flow is red→blue. The number in parentheses indicate the size of charge flow.

6 (NHSi + Rest)		
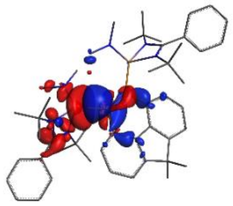 ΔE_{orb1} = -129.4 kcal/mol	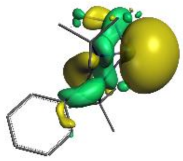 NHSi (HOMO)	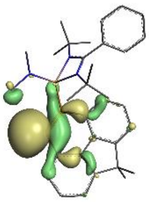 Rest (LUMO)
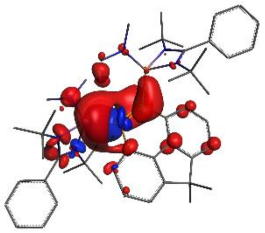 ΔE_{orb2} = -23.1 kcal /mol	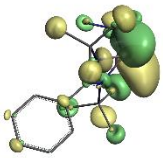 NHSi (LUMO+4)	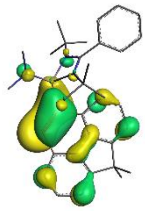 Rest (HOMO)
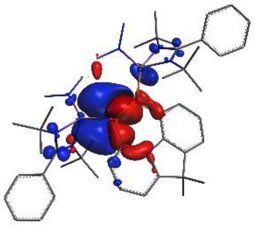 ΔE_{orb3} = -5.2 kcal /mol	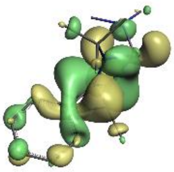 NHSi (LUMO+2)	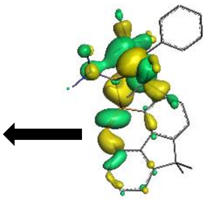 Rest (HOMO-5)

Figure S58. Plots of deformation densities Δρ of the pairwise orbital interactions and the shape of the most important interacting MOs of the chosen fragments of **6**. The direction of the charge flow is red→blue. The number in parentheses indicate the size of charge flow.

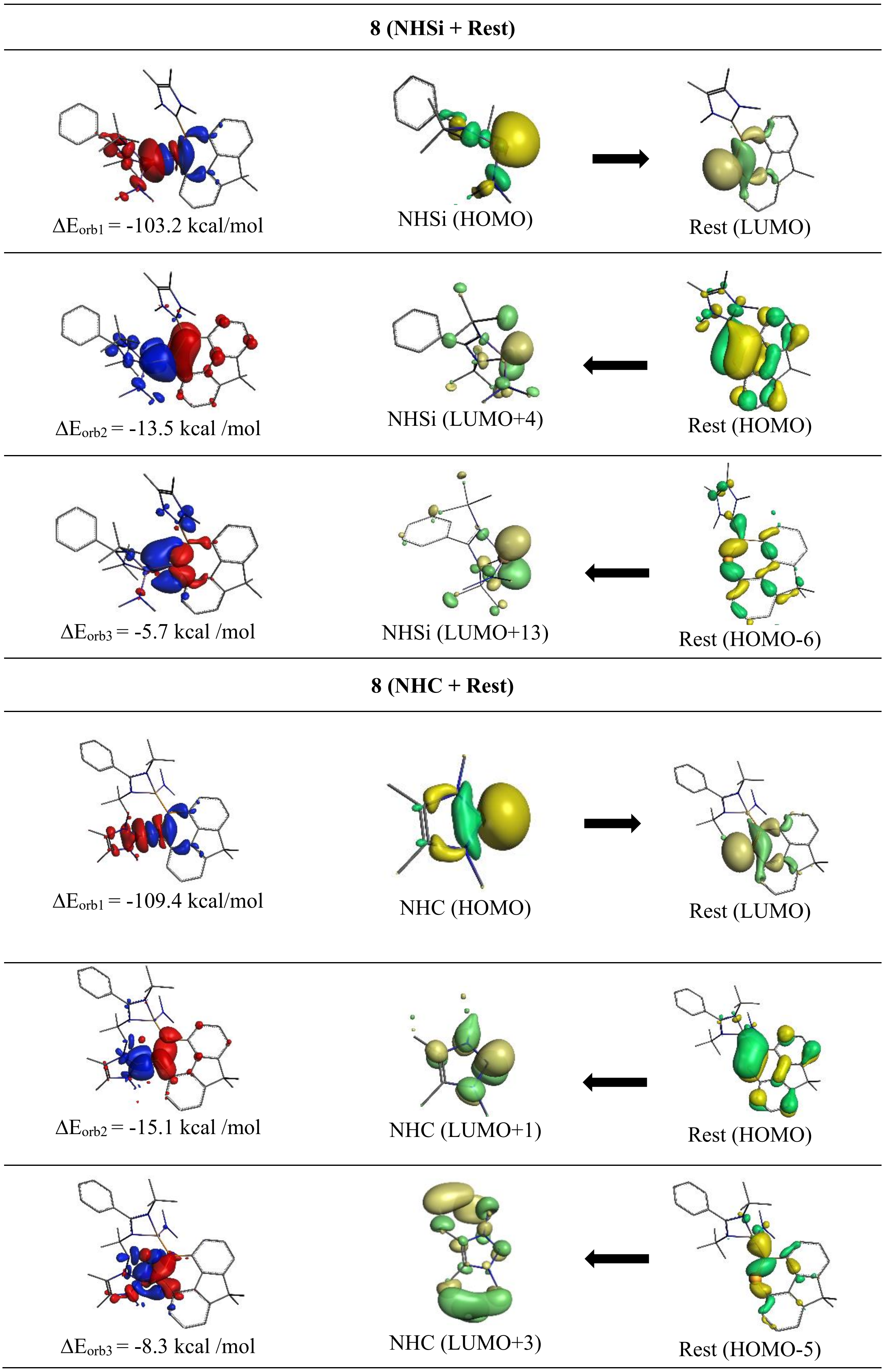

Figure S59. Plots of deformation densities Δρ of the pairwise orbital interactions and the shape of the most important interacting MOs of the chosen fragments of **8**. The direction of the charge flow is red→blue. The number in parentheses indicate the size of charge flow.

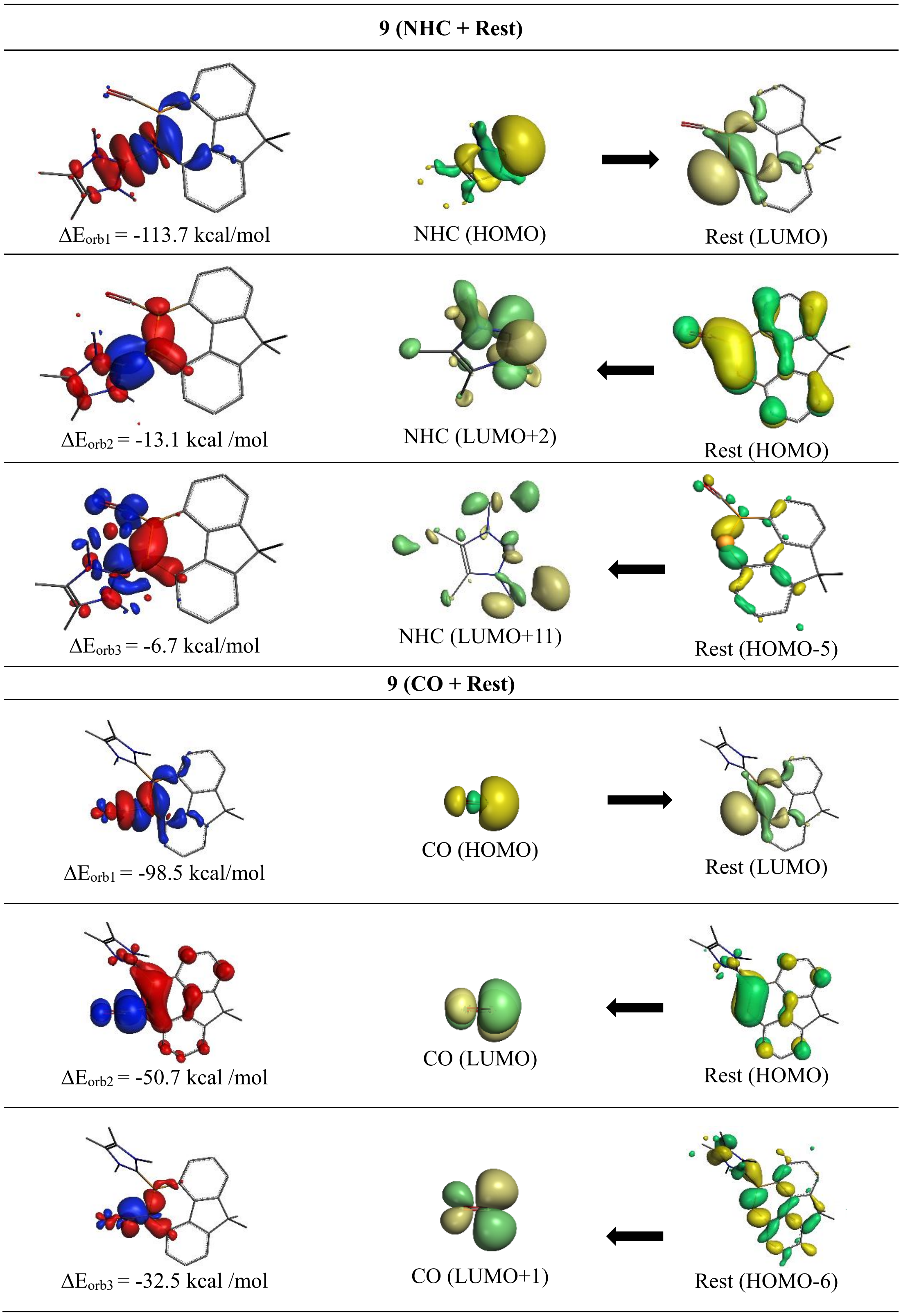

Figure S60. Plots of deformation densities Δρ of the pairwise orbital interactions and the shape of the most important interacting MOs of the chosen fragments of **9**. The direction of the charge flow is red→blue. The number in parentheses indicate the size of charge flow.

Table S22. Calculated NICS values of complexes **6**, **8** and **9** and the all-carbon phenanthrene analogue **9-C**.

	**6**	**8**	**9**	**9-C**
	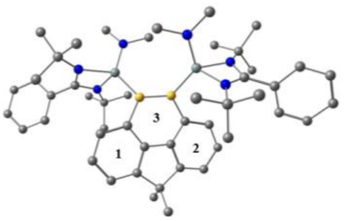	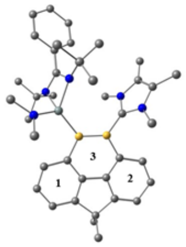	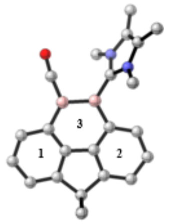	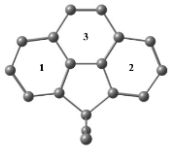
NICS(0)-1	-8.97	-9.47	-8.82	-13.5
NICS(0)-2	-8.96	-9.14	-10.12	-13.6
NICS(0)-3	2.63	2.35	6.84	-3.3
NICS(1)-ZZ-1	-24.71	-24.72	-24.87	-28.6
NICS(1)-ZZ-2	-25.22	-24.23	-24.69	-28.8
NICS(1)-ZZ-3	-14.31	-13.77	-9.53	-21.1
NICS(-1)-ZZ-1	-25.22	-24.90	-24.21	-28.7
NICS(-1)-ZZ-2	-24.71	-24.34	-25.61	-28.8
NICS(-1)-ZZ-3	-14.31	-13.73	-9.88	-21.1

Table S23. EDA-NOCV results of **2** by using NHSi and Rest in different charge and electron state as interacting fragments at the BP86+(D3BJ)/TZ2P level of theory. Energy values are given in kcal/mol.

	**2**	**2**	**2**	**2**
**Fragments**	$NHSi^{0}$ (S) + $Rest^{0}$(S)	$NHSi^{0}$ (T) + $Rest^{0}$(T)	$NHSi^{-1}$(D) + $Rest^{+1}$(D)	$NHSi^{+1}$(D) + $Rest^{-1}$(D)
ΔE_{int}	-88.3	-160.3	-213.5	-223.7
ΔE_{Pauli}	200.4	341.4	233.3	158.3
ΔE_{disp}[a]	-27.4(9.5%)	-27.4(5.5%)	-27.4(6.1%)	-27.4(7.2%)
ΔE_{elstat}[a]	-117.3(40.6%)	-166.4(33.2%)	-184.9(41.4%)	-122.1(32.0%)
ΔE_{orb}[a]	-144.0(49.6%)	-307.9(61.3%)	-234.6(52.5%)	-232.5(60.8%)

[a]The values in parentheses give the percentage contribution to the total attractive interactions ΔE_{elstat} + ΔE_{orb} + ΔE_{disp}.

Table S24. EDA-NOCV results of **6** by using NHSi and Rest in different charge and electron state as interacting fragments at the BP86+(D3BJ)/TZ2P level of theory. Energy values are given in kcal/mol.

	**6**	**6**	**6**	**6**
**Fragments**	$NHSi^{0}$(S)+ $Rest^{0}$(S)	$NHSi^{0}$(T)+ $Rest^{0}$(T)	$NHSi^{-1}$(D)+ $Rest^{+1}$(D)	$NHSi^{+1}$(D)+ $Rest^{-1}$(D)
ΔE_{int}	-84.3	-163.8	-247.5	-200.2
ΔE_{Pauli}	271.5	318.0	265.9	247.2
ΔE_{disp}[a]	-27.7(7.8%)	-27.7(5.7%)	-27.7(5.8%)	-27.7(6.2%)
ΔE_{elstat}[a]	-158.7(44.6%)	-164.4(34.1%)	-203.1(42.5%)	-218.0(48.7%)
ΔE_{orb}[a]	-169.3(47.6%)	-289.8(60.2%)	-247.5(51.7%)	-201.7(45.1%)

[a]The values in parentheses give the percentage contribution to the total attractive interactions ΔE_{elstat} + ΔE_{orb} + ΔE_{disp}.

Table S25. EDA-NOCV results of **8** by using NHSi and Rest in different charge and electron state as interacting fragments at the BP86+(D3BJ)/TZ2P level of theory. Energy values are given in kcal/mol.

	**8**	**8**	**8**	**8**
**Fragments**	$NHSi^{0}$(S) + $Rest^{0}$(S)	$NHSi^{0}$(T) + $Rest^{0}$(T)	$NHSi^{-1}$(D) + $Rest^{+1}$(D)	$NHSi^{+1}$(D) + $Rest^{-1}$(D)
ΔE_{int}	-87.5	-158.1	-213.8	-203.3
ΔE_{Pauli}	183.3	388.2	205.7	263.6
ΔE_{disp}[a]	-26.0(9.6%)	-26.0(4.8%)	-26.0(6.2%)	-26.0(5.6%)
ΔE_{elstat}[a]	-109.2(40.3%)	-191.6(35.0%)	-182.8(43.6%)	-254.0(54.4%)
ΔE_{orb}[a]	-135.6(50.1%)	-328.7(60.2%)	-210.7(50.2%)	-186.8(40.0%)

[a]The values in parentheses give the percentage contribution to the total attractive interactions ΔE_{elstat} + ΔE_{orb} + ΔE_{disp}.

Table S26. EDA-NOCV results of **8** by using NHC and Rest in different charge and electron state as interacting fragments at the BP86+(D3BJ)/TZ2P level of theory. Energy values are given in kcal/mol.

	**8**	**8**	**8**
**Fragments**	NHC^{0}(S) + $Rest^{0}$(S)	NHC^{0}(T) + $Rest^{0}$(T)	NHC^{-1}(D) + $Rest^{+1}$(D)
ΔE_{int}	-97.3	-497.9	-246.1
ΔE_{Pauli}	233.1	339.5	254.8
ΔE_{disp}[a]	-21.9(6.6%)	-21.9(2.6%)	-21.9(4.4%)
ΔE_{elstat}[a]	-157.0(47.5%)	-194.1(23.2%)	-230.8(46.1%)
ΔE_{orb}[a]	-151.6(45.9%)	-621.5(74.2%)	-248.2(49.6%)

[a]The values in parentheses give the percentage contribution to the total attractive interactions ΔE_{elstat} + ΔE_{orb} + ΔE_{disp}.

Table S27. EDA-NOCV results of **9** by using NHC and Rest in different charge and electron state as interacting fragments at the BP86+(D3BJ)/TZ2P level of theory. Energy values are given in kcal/mol.

	**9**	**9**	**9**
**Fragments**	NHC^0(S) + $Rest^0$(S)	NHC^0(T) + $Rest^0$(T)	NHC^{-1}(D) + $Rest^{+1}$(D)
ΔE_{int}	-107.3	-509.1	-294.2
ΔE_{Pauli}	205.4	297.6	212.8
ΔE_{disp}[a]	-10.4(3.3%)	-10.4(1.3%)	-10.4(2.0%)
ΔE_{elstat}[a]	-152.7(48.8%)	-170.4(21.1%)	-222.6(43.9%)
ΔE_{orb}[a]	-149.6(47.9%)	-625.8(77.6%)	-273.9(54.0%)

[a]The values in parentheses give the percentage contribution to the total attractive interactions ΔE_{elstat} + ΔE_{orb} + ΔE_{disp}.

Table S28. EDA-NOCV results of **9** by using CO and Rest in different charge and electron state as interacting fragments at the BP86+(D3BJ)/TZ2P level of theory. Energy values are given in kcal/mol.

	**9**	**9**	**9**	**9**
**Fragments**	CO^0(S) + $Rest^0$(S)	CO^0(T)+ $Rest^0$(T)	CO^{-1}(D)+ $Rest^{+1}$(D)	CO^{+1}(D)+ $Rest^{-1}$(D)
ΔE_{int}	-78.5	-412.9	-238.2	-381.0
ΔE_{Pauli}	229.3	177.0	272.9	221.0
ΔE_{disp}[a]	-4.2(1.3%)	-4.2(0.7%)	-4.2(0.8%)	-4.2(0.7%)
ΔE_{elstat}[a]	-112.8(36.6%)	-108.6(18.4%)	-241.8(47.3%)	-240.7(40.0%)
ΔE_{orb}[a]	-190.8(62.1%)	-477.1(80.9%)	-265.1(51.9%)	-357.0(59.4%)

[a]The values in parentheses give the percentage contribution to the total attractive interactions ΔE_{elstat} + ΔE_{orb} + ΔE_{disp}.

Table S29. Cartesian coordinates of the optimized geometry at the BP86+D3BJ/def2-TZVP level.

E = -2943.041144 Hartree

Compound **2**

0 1

Si	-2.10474500	-1.39063700	-0.11546100
Si	2.09649500	-1.23349000	0.35089800
O	-0.11582200	2.40903400	0.99676100
N	-3.48121300	-1.03181900	1.11858600
N	-3.76467400	-1.27451500	-1.00777900
N	-1.71296200	-3.06606600	0.02340000
N	3.61704100	-0.40046800	1.09965200
N	3.62546300	-1.61970300	-0.67411400
N	1.82348300	-2.67250300	1.27910300
C	-1.65583500	1.44092000	-0.56813500
C	-2.67883500	1.73634800	-1.50597100
H	-3.14849100	0.91846900	-2.05177900
C	-3.02188800	3.04974200	-1.83938000
H	-3.80005800	3.22852800	-2.58648200
C	-2.28907300	4.14386500	-1.33859800
H	-2.46023900	5.14203200	-1.74532500
C	-1.28220900	3.90907000	-0.39951600
C	-1.14948500	2.59059000	0.06596500
C	-0.08422500	4.84243000	-0.06073400
C	-0.05545300	6.06346400	-0.98531800
H	-0.01718600	5.77131800	-2.04409900
H	0.82236300	6.68906700	-0.76560800
H	-0.94977700	6.68449100	-0.82838700
C	-0.13980000	5.33260600	1.40604200
H	-1.04228100	5.94301800	1.56332800
H	0.74129000	5.95423900	1.62767900
H	-0.16008100	4.48827100	2.10385100
C	0.99125400	2.60543000	0.15900000
C	1.14335700	3.92056300	-0.30930200
C	2.21039800	4.15338100	-1.18030400
H	2.40087400	5.14849800	-1.58584600
C	2.98448000	3.06052500	-1.62004800
H	3.80581900	3.24120200	-2.31916700
C	2.63796800	1.74756600	-1.28853600
H	3.15224100	0.92554000	-1.78920900
C	1.56278400	1.45852700	-0.41256000
C	-8.12273400	-0.75734700	0.66146400
H	-8.93627200	-1.41964900	0.96212600
C	-8.38703300	0.57424500	0.32794400
H	-9.40946500	0.95380900	0.36765400

C	-7.34039200	1.41935300	-0.05649600
H	-7.54243700	2.46005300	-0.31525000
C	-6.03296200	0.93724100	-0.11652700
H	-5.20878300	1.58350800	-0.42637000
C	-5.76768000	-0.39871800	0.22236500
C	-6.81427100	-1.24377800	0.61448700
H	-6.60029000	-2.28023800	0.88052000
C	-4.38128000	-0.90929600	0.12109100
C	-4.29561600	-1.71344300	-2.30996300
C	-4.94076500	-3.10162800	-2.14242900
H	-4.21428300	-3.81597000	-1.73133700
H	-5.29297400	-3.48101000	-3.11333100
H	-5.80329700	-3.04830300	-1.46235200
C	-5.32269100	-0.73598300	-2.90595400
H	-6.25183400	-0.71094200	-2.32357900
H	-5.57098700	-1.05818700	-3.92800200
H	-4.91862500	0.28391300	-2.95770000
C	-3.08065500	-1.81661500	-3.24543100
H	-2.58326400	-0.84314900	-3.35720100
H	-3.39852100	-2.16754200	-4.23740900
H	-2.34289300	-2.52833600	-2.84675100
C	-3.51003900	-0.42399500	2.47312000
C	-4.62038400	-1.07754700	3.31220200
H	-5.61760900	-0.83779500	2.92071200
H	-4.56517500	-0.71028900	4.34763400
H	-4.50099300	-2.17120700	3.32730200
C	-3.70317800	1.09991000	2.41043900
H	-2.96083300	1.55494700	1.74139600
H	-3.57695600	1.52816800	3.41588600
H	-4.70743300	1.36469400	2.05548600
C	-2.14504900	-0.73242500	3.10001700
H	-1.97583500	-1.81835400	3.14531600
H	-2.10353400	-0.33272000	4.12298800
H	-1.33953000	-0.26963800	2.50833800
C	-2.56310400	-4.06303500	0.64275900
H	-3.39333600	-3.57935900	1.17429000
H	-1.99232200	-4.66799100	1.37385300
H	-2.98819700	-4.76689700	-0.10097500
C	-0.61168700	-3.62440300	-0.74222000
H	-0.97837300	-4.29732900	-1.54409200
H	0.07830900	-4.20277800	-0.10233000
H	-0.03968700	-2.80902300	-1.20451000
C	8.48758300	0.24995600	-0.55416100
H	9.53132800	0.52269600	-0.71886800

C	8.17157000	-0.93883500	0.11077500
H	8.96620600	-1.59542500	0.46896200
C	6.83675800	-1.29028200	0.31949200
H	6.58454400	-2.21541400	0.84001800
C	5.80960600	-0.45010600	-0.13372600
C	6.12799300	0.74723900	-0.79119900
H	5.32356600	1.40713800	-1.11892100
C	7.46441000	1.09068400	-1.00233200
H	7.70560500	2.02358700	-1.51454500
C	4.39149900	-0.81646500	0.09860200
C	3.88992800	0.43409400	2.28430000
C	2.56214500	0.49958800	3.05608000
H	2.24960200	-0.50511200	3.37861500
H	1.76429500	0.92556600	2.43048300
H	2.68035100	1.12717700	3.95057700
C	4.96454000	-0.23589300	3.15763200
H	5.08687300	0.32488100	4.09605000
H	5.93698000	-0.25977800	2.64710000
H	4.67112300	-1.26694700	3.40529000
C	4.32518600	1.85699900	1.89491000
H	3.58983400	2.31671600	1.22241500
H	5.30487100	1.85404900	1.39974400
H	4.40654600	2.47373900	2.80223600
C	3.90578000	-2.27095300	-1.96840300
C	2.61216900	-3.00476200	-2.34886200
H	1.78337800	-2.28701300	-2.44345400
H	2.34430400	-3.74564600	-1.58186000
H	2.73890300	-3.52369700	-3.30937100
C	4.24416700	-1.24587100	-3.06416600
H	4.42179000	-1.76612400	-4.01738800
H	5.14907200	-0.67533500	-2.81617700
H	3.41014700	-0.54528900	-3.20724200
C	5.05104800	-3.28942300	-1.82951400
H	4.83795800	-4.00552900	-1.02253400
H	6.00681300	-2.79349400	-1.61672600
H	5.16246000	-3.85350100	-2.76741700
C	0.69196100	-2.73798800	2.18918000
H	1.00572400	-2.57628800	3.24088600
H	0.19387500	-3.72427200	2.13912200
H	-0.03674000	-1.96601900	1.92219300
C	2.88693300	-3.59187000	1.64218500
H	3.72345900	-3.51015900	0.93659500
H	2.52257200	-4.63610300	1.62486000
H	3.27440800	-3.39528300	2.66302500

B	-0.85202700	0.07658200	-0.34045900
B	0.77203200	0.07997600	-0.20927700

E = -2867.844557 Hartree

Compound **6**

0 1

Si	2.13610800	-1.34085200	-0.19715100
Si	-2.13609900	-1.34113400	0.19669400
N	3.52820400	-0.49942200	-1.14640700
N	3.76992200	-1.57247600	0.70283300
N	1.83140500	-2.84373800	-0.99543800
N	-3.76993200	-1.57187300	-0.70337600
N	-3.52809600	-0.50032700	1.14671800
N	-1.83140600	-2.84452800	0.99403700
C	1.38595200	1.34961700	0.66444400
C	2.60535300	1.64218600	1.33944600
H	3.23000900	0.81631900	1.68565700
C	3.02340800	2.94696900	1.59999300
H	3.96120900	3.11127600	2.13945100
C	2.26151500	4.07028400	1.19453100
H	2.62306800	5.08195900	1.39492000
C	1.05125700	3.83339300	0.55829500
C	0.63560800	2.50421900	0.33083000
C	-0.00009800	4.80424100	0.00001400
C	-0.59730000	5.68361700	1.11556700
H	-1.02178800	5.05831400	1.91366200
H	-1.39751500	6.32622600	0.71681200
H	0.17660400	6.33050800	1.55678600
C	0.59702800	5.68372100	-1.11547200
H	1.39723600	6.32632300	-0.71669500
H	-0.17690900	6.33063200	-1.55660300
H	1.02150800	5.05851100	-1.91364500
C	-0.63576700	2.50419100	-0.33068900
C	-1.05142000	3.83334800	-0.55825100
C	-2.26161200	4.07018500	-1.19463400
H	-2.62318200	5.08184300	-1.39507700
C	-3.02342800	2.94683900	-1.60014700
H	-3.96116300	3.11110500	-2.13973300
C	-2.60536600	1.64206800	-1.33953200
H	-3.22990900	0.81616100	-1.68584800
C	-1.38603800	1.34955300	-0.66437800
C	-4.41185100	-0.78634300	0.19014200
C	-5.81035000	-0.30664900	0.10445700
C	-6.07586200	0.99810000	-0.33660900

H	-5.24529900	1.64428200	-0.62530100
C	-7.39424000	1.44888900	-0.41390000
H	-7.59638100	2.46343200	-0.76142700
C	-8.45011900	0.60814700	-0.04780300
H	-9.47967300	0.96496700	-0.10812100
C	-8.18485600	-0.69006900	0.39881800
H	-9.00478600	-1.34704900	0.69372800
C	-6.86872500	-1.14905600	0.47310200
H	-6.65529400	-2.15481800	0.83785900
C	-4.20865200	-2.15475700	-1.98479000
C	-5.11243600	-3.37396600	-1.72368600
H	-4.59835900	-4.10648200	-1.08565000
H	-5.37136700	-3.86477300	-2.67381100
H	-6.04762700	-3.07166700	-1.23273100
C	-4.94569800	-1.14211400	-2.87711400
H	-5.89514800	-0.81854500	-2.43274400
H	-5.16785400	-1.60919900	-3.84801500
H	-4.32378800	-0.25509600	-3.06030900
C	-2.92360100	-2.61366400	-2.69054400
H	-2.25451300	-1.76002600	-2.87124800
H	-3.16752400	-3.08346300	-3.65381200
H	-2.38557000	-3.34700000	-2.07173700
C	-3.61270800	0.30898700	2.37669100
C	-4.86236000	-0.06685900	3.18820600
H	-4.83293300	0.44379800	4.16179200
H	-4.89565800	-1.15192500	3.36806000
H	-5.78663300	0.23252200	2.67710900
C	-3.60972800	1.81264800	2.05173100
H	-4.51463400	2.10200700	1.50188500
H	-2.73485100	2.07634000	1.44410400
H	-3.57940500	2.39042300	2.98761000
C	-2.34796900	-0.03771800	3.18006600
H	-2.32068100	0.55108700	4.10765600
H	-1.44383300	0.18805100	2.59279700
H	-2.33395600	-1.10600200	3.44362500
C	-2.90144500	-3.69075800	1.49190200
H	-3.00549100	-3.61022400	2.59289200
H	-2.70971200	-4.75399700	1.25500600
H	-3.85981100	-3.40976100	1.03681200
C	-0.54039800	-3.15025700	1.58843700
H	0.17711600	-2.35359800	1.34862900
H	-0.13870900	-4.11375800	1.22351800
H	-0.61884500	-3.21193600	2.69203300
C	4.41192300	-0.78628200	-0.19006100

C	5.81045700	-0.30679400	-0.10394900
C	6.86888100	-1.14937700	-0.47205300
H	6.65547900	-2.15513700	-0.83682200
C	8.18504000	-0.69055100	-0.39728200
H	9.00500400	-1.34767700	-0.69177700
C	8.45029500	0.60767300	0.04932300
H	9.47987300	0.96436100	0.11002700
C	7.39437600	1.44859600	0.41488000
H	7.59650100	2.46314700	0.76239000
C	6.07597300	0.99797300	0.33707300
H	5.24538700	1.64429500	0.62536900
C	3.61316900	0.30988000	-2.37636200
C	2.34843600	-0.03650000	-3.17989900
H	2.33423100	-1.10475700	-3.44354800
H	2.32134700	0.55239200	-4.10744100
H	1.44429800	0.18938000	-2.59267900
C	4.86282800	-0.06620800	-3.18774200
H	5.78711500	0.23276800	-2.67643700
H	4.83373100	0.44465500	-4.16123000
H	4.89579600	-1.15125000	-3.36781100
C	3.61042700	1.81353500	-2.05136500
H	2.73559700	2.07734100	-1.44372700
H	3.58016900	2.39131800	-2.98724100
H	4.51537300	2.10277600	-1.50153000
C	4.20821900	-2.15529100	1.98443900
C	4.94484200	-1.14264600	2.87708800
H	5.89448100	-0.81903600	2.43315200
H	5.16659700	-1.60977000	3.84806100
H	4.32281300	-0.25566900	3.06007000
C	5.11218300	-3.37444500	1.72360100
H	4.59841700	-4.10687900	1.08521600
H	5.37070300	-3.86538200	2.67377100
H	6.04757800	-3.07205200	1.23309600
C	2.92295900	-2.61437100	2.68969700
H	2.25367200	-1.76085000	2.87019800
H	3.16659400	-3.08421700	3.65302000
H	2.38525000	-3.34774600	2.07065700
C	0.54049100	-3.14922300	-1.59015100
H	-0.17692600	-2.35241400	-1.35051900
H	0.13850800	-4.11263400	-1.22531600
H	0.61922000	-3.21094400	-2.69372400
C	2.90138000	-3.69021900	-1.49300300
H	3.00580600	-3.60963800	-2.59395300
H	2.70927200	-4.75342600	-1.25626200

H	3.85966600	-3.40951600	-1.03756400
B	0.77048600	-0.03041800	0.27091400
B	-0.77051700	-0.03044600	-0.27084600

E = -2131.994559 Hartree

Compound **8**

0 1

Si	-0.58460500	-1.18219800	0.70429800
N	-1.96086900	-1.81849300	-0.42738000
N	-2.13063600	-0.09122400	0.85811600
N	-0.54688400	2.44608400	-1.05718400
N	0.51207500	3.38960800	0.57610700
N	-0.61964800	-2.31872600	2.01440100
C	-2.79640000	-0.84032700	-0.03903400
C	3.60992600	-1.00416800	-0.09240800
C	-4.20224300	-0.65230300	-0.47984400
C	0.49940200	2.25431800	-0.19436400
C	3.92505900	0.37672400	-0.32524100
C	2.31195600	-1.53338400	0.12871800
C	2.99234200	1.43999100	-0.37788100
C	2.30144700	-2.94484300	0.32961400
H	1.35149300	-3.45128800	0.52158900
C	-1.98875300	-2.68557200	-1.62554900
C	-6.53313900	-1.31457500	-0.32542400
H	-7.30196500	-1.95948600	0.10301500
C	-2.65390900	0.76848600	1.94178100
C	-4.55666000	0.33417000	-1.41038300
H	-3.78845200	0.98642000	-1.82600300
C	-5.20175200	-1.47776300	0.05851300
H	-4.92665600	-2.25088300	0.77710900
C	-0.50280400	4.27209100	0.20044200
C	4.79826800	-1.76617000	-0.12409700
C	-3.38782500	1.99534300	1.38074500
H	-2.72096400	2.57150800	0.72577800
H	-3.70145100	2.64809200	2.20912300
H	-4.28591200	1.70924000	0.81845100
C	-1.17050300	3.67749100	-0.83973400
C	-6.87877800	-0.33432100	-1.26072900
H	-7.91912100	-0.21163700	-1.56593300
B	1.13322500	-0.50403300	0.11189300
C	5.31524900	0.52126100	-0.51938100
C	3.46409600	-3.71427500	0.30178000
H	3.39243600	-4.79365200	0.46211800
C	4.73629800	-3.13729300	0.07340500

H	5.63054800	-3.76577400	0.05654500
C	-3.59174400	-0.03191900	2.86300800
H	-4.50759100	-0.33320100	2.33666500
H	-3.88525800	0.58402900	3.72603600
H	-3.08599100	-0.93206200	3.23747600
C	-0.86381200	1.52129700	-2.12763300
H	-0.07688700	0.75195200	-2.11762500
H	-0.89104000	2.04247100	-3.09539300
H	-1.82887600	1.03114000	-1.94840000
C	3.56967000	2.70636100	-0.68253000
H	2.92306700	3.58393700	-0.79293100
C	5.99814200	-0.84751400	-0.39587700
C	1.42586000	3.58603600	1.69103400
H	1.92592700	2.62682400	1.87624600
H	0.86705500	3.89966900	2.58382600
H	2.19000300	4.33756700	1.44936700
C	6.99415000	-0.86867200	0.78001500
H	7.42793100	-1.87320200	0.90298800
H	6.49031300	-0.59309600	1.71727100
H	7.81636100	-0.15705200	0.60620400
C	-1.42671400	1.22640100	2.74242300
H	-0.91833600	0.36772300	3.20386900
H	-1.73488300	1.91802300	3.53974400
H	-0.70433700	1.72829900	2.08696000
C	-5.88720900	0.48673600	-1.80446600
H	-6.14945900	1.25252400	-2.53633500
B	1.49632600	1.06073800	-0.15838300
C	-1.70811000	-3.21289300	2.35509100
H	-2.15654800	-2.96751100	3.33906400
H	-1.35524900	-4.26075900	2.41494800
H	-2.49903300	-3.16520400	1.59521700
C	0.46504000	-2.33867900	2.98813600
H	1.26251700	-1.65354700	2.67098500
H	0.90075600	-3.35015300	3.07807600
H	0.10717400	-2.03196000	3.99117100
C	4.94169600	2.86978000	-0.87340200
H	5.33664900	3.86304700	-1.10402200
C	5.83924100	1.77730500	-0.78770700
H	6.90960100	1.93471300	-0.94467000
C	-1.92055700	-1.83625500	-2.90508800
H	-2.79239200	-1.17112500	-2.98762600
H	-1.90682400	-2.48798700	-3.79117200
H	-1.00298800	-1.23267500	-2.90717400
C	-0.73540000	-3.56620700	-1.53094600

H	0.17613500	-2.95118200	-1.50600000
H	-0.67925800	-4.23616700	-2.40024900
H	-0.76009400	-4.18165400	-0.62017300
C	-2.33452800	4.13768700	-1.64328800
H	-2.09046000	4.21642500	-2.71468100
H	-2.66333400	5.12769000	-1.30263100
H	-3.19212200	3.45091900	-1.54890700
C	6.71044700	-1.23499900	-1.70615500
H	7.52811200	-0.53135400	-1.92730000
H	6.00411200	-1.22017100	-2.54814200
H	7.13779000	-2.24695400	-1.63070600
C	-3.24175700	-3.57637000	-1.64054200
H	-3.33785300	-4.12468600	-0.69209600
H	-3.15500500	-4.31263500	-2.45288800
H	-4.15839800	-2.99634600	-1.80579400
C	-0.73748200	5.56529200	0.89647300
H	-1.00318900	5.41720200	1.95590800
H	-1.56269200	6.10915400	0.41972600
H	0.15382600	6.21172500	0.86838900

E = -1125.882500 Hartree

Compound **9**

0 1

B	-0.70973100	0.24236700	0.07435300
B	-0.04703200	1.72496100	-0.18191100
C	0.29077500	-0.93015200	0.25266000
C	0.01194200	-2.29678800	0.53065800
H	-1.02612500	-2.62341600	0.65113100
C	1.03061100	-3.23912800	0.67041400
H	0.77418500	-4.27960100	0.88433300
C	2.39407800	-2.87900700	0.54872700
H	3.16958500	-3.64073600	0.66350900
C	2.70906800	-1.55218600	0.29396600
C	1.66585100	-0.61255500	0.15591100
C	4.06527400	-0.85149000	0.12750500
C	4.83477200	-1.41106700	-1.08509700
H	4.23261200	-1.32478000	-2.00048500
H	5.08371900	-2.47239900	-0.93151500
H	5.77339700	-0.85641400	-1.23565300
C	4.91671700	-0.97654100	1.40605900
H	5.85584800	-0.41235700	1.30070100
H	5.16910800	-2.02971000	1.60380200
H	4.37223200	-0.58126600	2.27502700

C	2.22488000	0.69135700	-0.08440900
C	3.63166700	0.60213300	-0.11108400
C	4.37431400	1.75372400	-0.32887200
H	5.46670200	1.73887600	-0.35856700
C	3.67900700	2.97104100	-0.51411400
H	4.25210800	3.88537300	-0.68675600
C	2.28410300	3.04070600	-0.48172800
H	1.79920600	4.00943800	-0.62943700
C	1.49453100	1.88535800	-0.26019400
C	-1.09383000	2.71570400	-0.38096400
C	-2.24597300	-0.05231100	0.05146200
C	-2.98061700	1.37635200	1.96178400
H	-1.89545000	1.51327900	2.04794800
H	-3.38109300	0.94640700	2.89023600
H	-3.44960000	2.35076200	1.77189500
C	-2.25873500	-1.58946500	-1.90978300
H	-2.73031400	-1.31606800	-2.86368700
H	-2.33153100	-2.67557500	-1.76239800
H	-1.20187400	-1.30200200	-1.92529700
C	-4.27881400	-0.86584900	-0.57413500
C	-4.48088200	-0.01342000	0.48149200
C	-5.72820000	0.39753100	1.17985800
H	-5.71449500	0.11748200	2.24519200
H	-6.59731400	-0.08776200	0.71871700
H	-5.88289100	1.48664400	1.12602900
C	-5.23952400	-1.65912300	-1.38689700
H	-5.22774900	-1.35635600	-2.44615800
H	-6.26035500	-1.51658800	-1.01142200
H	-5.01453900	-2.73664300	-1.34885400
N	-3.22301100	0.47120200	0.84597300
N	-2.90394600	-0.87714600	-0.81460400
O	-2.04611800	3.39242900	-0.48992300

E = -656.549130 Hartree

Compound **9-C**

0 1

C	1.46103200	1.60901700	-0.00002400
C	3.38907500	0.12588100	0.00006200
H	4.47395100	0.00092900	-0.00002500
C	2.57732300	-1.04290300	0.00007600
H	3.05375000	-2.02594200	-0.00006000
C	1.20003800	-0.88736700	0.00020400
C	0.70450600	0.42702900	0.00022700

C	-0.00002300	-1.86447800	-0.00004800
C	-0.00005700	-2.74212700	1.26611400
H	-0.00003800	-2.12098700	2.17261500
H	0.89111600	-3.38719100	1.28783900
H	-0.89128400	-3.38711900	1.28782900
C	-0.00000600	-2.74176200	-1.26644700
H	-0.89123900	-3.38674000	-1.28843400
H	0.89113900	-3.38686400	-1.28831700
H	0.00009500	-2.12037900	-2.17278100
C	-0.70449800	0.42704500	0.00027100
C	-1.20006000	-0.88733900	0.00013800
C	-2.57734200	-1.04285800	-0.00004100
H	-3.05377100	-2.02589500	-0.00023200
C	-3.38906900	0.12594700	-0.00006100
H	-4.47395000	0.00102700	-0.00020400
C	-2.86669400	1.42014600	0.00003400
H	-3.54289400	2.27804100	-0.00005500
C	-1.46100600	1.60904400	0.00016900
C	2.86671600	1.42009100	0.00004500
H	3.54294300	2.27794000	-0.00006100
C	0.69151600	2.83137600	-0.00048900
C	-0.69142200	2.83137200	0.00005000
H	1.22101200	3.78728200	-0.00050800
H	-1.22101900	3.78720900	0.00071800

REFERENCES

1 (a) Liu, T.-T.; Chen, J.; Chen, X.-L.; Ma, L.; Guan, B.-T.; Lin, Z.; Shi, Z.-J. Neutral Boryl Radicals in Mixed-Valent B(III)Br-B(II) Adducts. *Chem.–Eur. J.* **2023,** *29*, e202202634. (b) So, C. W.; Roesky, H. W.; Gurubasavaraj, P. M.; Oswald, R. B.; Gamer, M. T.; Jones, P. G.; Blaurock, S. Synthesis and Structures of Heteroleptic Silylenes. *J. Am. Chem. Soc.* **2007**, *129*, 12049-12054.

2 Sheldrick, G. Crystal Structure Refinement with SHELXL. Acta Crystallogr. Sect. C 2015, 71, 3–8.

3 Dolomanov, O. V; Bourhis, L. J.; Gildea, R. J.; Howard, J. A. K.; Puschmann, H. OLEX2: A Complete Structure Solution, Refinement and Analysis Program. J. Appl. Crystallogr. 2009, 42, 339–341.

4 Frisch, M. J.; Trucks, G. W.; Schlegel, H. B.; Scuseria, G. E.; Robb, M. A.; Cheeseman, J. R.; Scalmani, G.; Barone, V.; Petersson, G. A.; Nakatsuji, H.; Li, X.; Caricato, M.; Marenich, A. V.; Bloino, J.; Janesko, B. G.; Gomperts, R.; Mennucci, B.; Hratchian, H. P.; Ortiz, J. V.; Izmaylov, A. F.; Sonnenberg, J. L.; Williams-Young, D.; Ding, F.; Lipparini, F.; Egidi, F.; Goings, J.; Peng, B.; Petrone, A.; Henderson, T.; Ranasinghe, D.; Zakrzewski, V. G.; Gao, J.; Rega, N.; Zheng, G.; Liang, W.; Hada, M.; Ehara, M.; Toyota, K.; Fukuda, R.; Hasegawa, J.; Ishida, M.; Nakajima, T.; Honda, Y.; Kitao, O.; Nakai, H.; Vreven, T.; Throssell, K.; Montgomery, J. A.; Jr.,; Peralta, J. E.; Ogliaro, F.; Bearpark, M. J.; Heyd, J. J.; Brothers, E. N.; Kudin, K. N.; Staroverov, V. N.; Keith, T. A.; Kobayashi, R.; Normand, J.; Raghavachari, K.; Rendell, A. P.; Burant, J. C.; Iyengar, S. S.; Tomasi, J.; Cossi, M.; Millam, J. M.; Klene, M.; Adamo, C.; Cammi, R.; Ochterski, J. W.; Martin, R. L.; Morokuma, K.; Farkas, O.; Foresman, J. B.; Ortiz, J.V.; Cioslowski, J.; Fox, D. J., *Gaussian 16*, *Revision A.03*, *Gaussian, Inc, Wallingford CT* . **2016**.

5 Becke, A. D., Density-functional exchange-energy approximation with correct asymptotic behavior. *Phys. Rev.* **1988**, *A38*, 3098-3100.

6 Perdew, J. P., Density-functional approximation for the correlation energy of the inhomogeneous electron gas. *Phys. Rev.* **1986**, *B33*, 8822-8824.

7 Zhao, Y.; Truhlar, D. G., The M06 suite of density functionals for main group thermochemistry, thermochemical kinetics, noncovalent interactions, excited states, and transition elements: Two new functionals and systematic testing of four M06-class functionals and 12 other functionals. *Theor. Chem. Acc*. **2008**, *120*, 215-241.

8 Weigend, F., Accurate coulomb-fitting basis sets for H to Rn. *Phys. Chem. Chem. Phys.* **2006**, *8*, 1057-1065.

9 Weigend, F.; Ahlrichs, R., Balanced basis sets of split valence, triple zeta valence and quadruple zeta valence quality for H to Rn: Design and assessment of accuracy. *Phys. Chem. Chem. Phys.* **2005**, *7*, 3297-3305.

10 Grimme, S.; Antony, J.; Ehrlich, S.; Krieg, H., A consistent and accurate ab initio parametrization of density functional dispersion correction (DFT-D) for the 94 elements H-Pu. *J. Chem. Phys.* **2010**, *132,* 154104.

11 Reed, A. E.; Curtiss, L. A.; Weinhold, F., Intermolecular interactions from a natural bond orbital, donor-acceptor viewpoint. *Chem. Rev.* **1988**, *88*, 899-926.

12 Glendening, E. D.; Landis, C. R.; and Weinhold, F. NBO 7.0: New Vistas in Localized and Delocalized Chemical Bonding Theory. *J. Comput. Chem.* **2019**, *40*, 2234-2241.

13 Mayer, I., Charge, bond order and valence in the AB initio SCF theory. *Chem. Phys. Lett.* **1983**, *97*, 270 .

14 Mayer, I., Bond order and valence: Relations to Mulliken's population analysis. *Int. J. Quantum Chem.* **1984**, *26*, 151.

15 Mayer, I., Bond order and valence indices: a personal account. *J. Comput. Chem.,* **2006**, *28,* 204.

16 Lu, T.; Chen, F., Multiwfn: A multifunctional wavefunction analyzer. *J. Comput. Chem.* **2012**, *33*, 580-592.

17 Legault, C. Y. CYLview, 1.0b; Université de Sherbrooke, Sherbrooke (Québec) Canada, **2009**; http://www.cylview.org

18 Ziegler, T.; Rauk, A., On the calculation of bonding energies by the Hartree Fock slater method. *Theor. Chim. Acta.* **1977**, *46*, 1-10.

19 Zhao, L.; Hopffgarten, M. V.; Andrada, D. M.; Frenking, G., Energy decomposition analysis. *WIREs Comput. Mol. Sci.* **2018**, *8*, e1345.

20 Mitoraj, M.; Michalak, A., Donor–acceptor properties of ligands from the natural orbitals for chemical valence. *Organometallics*. **2007**, *26*, 6576-6580.

21 Mitoraj, M.; Michalak, A., Applications of natural orbitals for chemical valence in a description of bonding in conjugated molecules. *J. Mol. Model.* **2008**, *14*, 681-687.

22 Amsterdam, S., Density Functional 2022 (ADF2022), Theoretical Chemistry, Vrije Universiteit, Amsterdam, Netherlands, http://www.scm.com.

23 Velde, G. T.; Bickelhaupt, F. M.; Baerends, E. J.; Guerra, C. F.; Gisbergen, S. J. A. V.; Snijders, J. G.; Ziegler, T., Chemistry with ADF. *J. Comput. Chem.* **2001**, *22*, 931-967.

24 Lenthe, E. V.; Baerends, E. J., Optimized slater-type basis sets for the elements 1–118. *J. Comput. Chem.* **2003**, *24*, 1142-1156.

25 Bickelhaupt, F. M.; Nibbering, N. M. M.; Wezenbeek, E. M. V.; Baerends, E. J., Central bond in the three CN.cntdot.dimers NC-CN, CN-CN and CN-NC: Electron pair bonding and Pauli repulsion effects. *J. Phys. Chem.* **1992**, *96*, 4864-4873.

26 Krapp, A.; Bickelhaupt, F. M.; Frenking, G., Orbital overlap and chemical bonding. *Chem. Eur. J.* **2006**, *12*, 9196-9216.

27 Zhao, L.; Pan, S.; Holzmann, N.; Schwerdtfeger, P.; Frenking, G., Chemical Bonding and Bonding Models of Main-Group Compounds. *Chem. Rev.* **2019**, *119*, 14, 8781–8845.

28 Zhao, L.; Hermann, M.; Schwarz, W. H. E.; Frenking, G., The Lewis electron-pair bonding model: Modern energy decomposition analysis. *Nat. Rev. Chem*. **2019**, *3*, 48-63.

29 Zhao, L.; Hermann, M.; Holzmann, N.; Frenking, G., Dative bonding in main group compounds. *Coord. Chem. Rev*. **2017**, *344*, 163-204.
